# A systematic review of clinical practice guidelines on the use of low molecular weight heparin and fondaparinux for the treatment and prevention of venous thromboembolism: Implications for research and policy decision-making

**DOI:** 10.1371/journal.pone.0207410

**Published:** 2018-11-09

**Authors:** Amy Johnston, Shu-Ching Hsieh, Marc Carrier, Shannon E. Kelly, Zemin Bai, Becky Skidmore, George A. Wells

**Affiliations:** 1 Cardiovascular Research Methods Centre, University of Ottawa Heart Institute, Ottawa, Ontario, Canada; 2 Department of Medicine, Division of Hematology, The Ottawa Hospital, Ottawa, Ontario, Canada; 3 Independent Information Specialist, Ottawa, Ontario, Canada; 4 School of Epidemiology and Public Health, University of Ottawa, Ottawa, Ontario, Canada; University of Glasgow, UNITED KINGDOM

## Abstract

**Background:**

Venous thromboembolism (VTE) is a major global cause of morbidity and mortality. Low molecular weight heparin (LMWH) and fondaparinux (FDP) are frequently used to treat and prevent VTE and have a variety of safety and practical advantages over other anticoagulants, including use in outpatient settings. These medications are commonly listed on drug formularies, which act as a gateway for health plan prescription coverage by outlining the circumstances under which patients will be covered for specific drugs and drug products. Because patient access to medications is impacted by the nature of their listing on formularies, they must be rigorously reviewed and modernized as new evidence emerges.

**Methods:**

As part of a broader drug class review team, we completed a systematic review of clinical practice guidelines to determine whether the recommendations they reported aligned with the indications listed for the coverage of LMWH and FDP in an outpatient drug formulary. Guideline quality was assessed using the Appraisal of Guidelines for Research & Evaluation (AGREE) II tool. Recommendation matrices were used to systematically compare, categorize, and summarize included recommendations.

**Results:**

Twenty-seven guidelines were included from which 168 eligible recommendations were identified. Generally, AGREE II domains were adequately addressed; however, domain five (*applicability*) was poorly addressed. Most recommendations were based on moderate- to low-quality/limited evidence and reported on the use of LMWHs generally; few reported on specific agents.

**Conclusions:**

Our findings contributed to the recommendation that the formulary listing for LMWH and FDP be streamlined to include coverage for specific outpatient indications. The paucity of available evidence on the comparative efficacy of specific LMWH agents against each other and FDP limited agent-specific listing recommendations, highlighting the need for high-quality comparative studies on this topic.

## Introduction

Venous thromboembolism (VTE) is a major global cause of morbidity and mortality [1–3]. No single class of antithrombotic agents is appropriate for use in all clinical situations. Low molecular weight heparins (LMWHs) are, however, often among the preferred medications used to treat and prevent this health issue because they have a variety of efficacy, safety, and practical advantages compared to other anticoagulants [4–7]. For example, these medications have a more predictable and reliable anticoagulant response compared to warfarin, and do not require frequent laboratory monitoring, which is often inconvenient for patients [6, 8]. Furthermore, compared to unfractionated heparin (UFH), LMWHs have longer half-lives and more predictable dose-responses, requiring only one or two administrations per day. LMWHs are also associated with a lower incidence of heparin induced thrombocytopenia (HIT) compared to UFHs and, since these medications can be self-administered via subcutaneous injection, outpatient use is possible [6]. In 2001, the emergence of FDP, a highly bioavailable heparin-derived antithrombotic agent, provided prescribers and patients with a new alternative to warfarin, UFH, and LMWH in specific circumstances [9–11]. For example, several clinical reports have suggested that FDP may be a safe alternative to LMWH for patients with a history of HIT [12].

Their applicability across a broad spectrum of clinical scenarios makes LMWH and FDP appealing for prescribers; however, research has shown that prescribing decisions are not based solely on clinical indication and medical knowledge [13–16]. Indeed, a range of direct and indirect factors are known to influence the prescription decision-making process, such as: patient demographics, health needs, preferences, and values; financing; practice guideline recommendations, as well as coverage on prescription drug plans through public and private formulary listings [14–16]. As they act as a gateway for health plan prescription coverage by listing medications that insurers will either provide or reimburse, drug formularies play a particularly integral role in prescribing decisions and, thus, in promoting patient access to anticoagulants such as LMWH and FDP [15].

While the exact nature of formularies can vary across jurisdictions, they often employ a tiered reimbursement scheme that includes: coverage for listed medications for all patients with no restrictions (general benefit), coverage for listed medications for patients meeting specific clinical criteria (limited use), or no coverage offered (non-benefit) [17, 18]. Certainly, formularies with variable, non-inclusive, or out of date listings can be burdensome for prescribers and may impact patient outcomes, especially if the indications they cover do not align with the treatment recommendations reported in most evidence-based clinical practice guidelines (CPGs) [16].

To ensure that formularies are kept up to date, they must be rigorously reviewed as new evidence emerges [15, 19–21]. To this end, we completed a systematic review (SR) of evidence-based CPGs to determine whether the recommendations they reported aligned with the indications covered for the use of LMWH and FDP in an outpatient drug formulary [16].

## Methods

We completed a SR of CPGs that reported recommendations on the use of LMWH and FDP for ten specific outpatient indications (see Table 1) that evolved through consensus discussions between clinicians (primary care physicians, specialists [oncologists, hematologists, internists, emergency physicians], and pharmacists) who prescribe or dispense LMWH and FDP to patients. The study protocol was peer-reviewed and published online prior to the start of review [22] and this manuscript was prepared in accordance with the PRISMA checklist for systematic reviews and meta-analysis [23] (S1 Appendix). A patient representative served as a member of the drug class research team and, along with patient advocacy groups, was invited to provide feedback on the comprehensive research plan, draft review, and final recommendations made to the OPDP [16, 24].

**Table 1:** Eligibility criteria pertaining to the population & clinical areas, interventions, comparators, attributes of CPGs and recommendation characteristics (PICAR) statement.

PICAR Element	Study Specific Criteria
**P**opulation & Clinical area(s)	- Adult (>18 years) outpatients - Ten clinical indications: **Treatment of:** **1)** VTE* in patients without cancer; **2)** symptomatic, acute, VTE in patients with cancer; **3)** VTE* in patients in whom treatment with warfarin is either not tolerated or contraindicated; **4)** VTE* in patients who fail treatment with warfarin; **5)** VTE* in pregnant and/or lactating women **Prevention of****: 6)** VTE in patients undergoing non-orthopedic surgery; **7)** VTE in patients with cancer **Post-operative prophylaxis of****: 8)**VTE* in patients undergoing hip or knee surgery and cannot use warfarin; **9)** VTE in patients undergoing surgery of the lower limbs **Peri-operative bridging for****: 10)** patients who require long-term warfarin and must discontinue due to surgery
**I**nterventions	- LMWH as a drug class or any individual agent - FDP
**C**omparators	- No comparator - Any antithrombotic pharmaceutical agent - Individual LMWH agents (head-to-head comparisons) - FDP compared to LMWH (drug class or any individual agent)
**A**ttributes of CPGs	- **Language:** English language - **Publishing region:** North America, Europe, Japan, Australia, & New Zealand - **Version:** Only the latest version of CPGs is of interest - **Development process:** CPGs are explicitly evidence-based^†^ - **System of rating evidence:** CPGs use a system to rate the level of evidence behind recommendations - **Scope:** CPGs primarily focused on the management of VTE - **Recommendations:** CPGs will only be included if they report one or more eligible recommendations of interest
**R**ecommendation characteristics	- **Interventions:**: Recommendations must explicitly discuss at least one intervention of interest - **Comparator(s):** Recommendations are not required to compare an intervention of interest to another antithrombotic agent. If such a comparison is made, the comparator must also meet specific eligibly criteria (see *Comparators*) - **Duration of treatment:** Recommendations on the duration of treatment with LMWH/ FDP are of particular interest - **Levels of confidence:** Each recommendation must be accompanied by an explicit level of confidence (e.g., GRADE 2A)

**Abbreviations:** VTE = venous thromboembolism; LMWH = low molecular weight heparin; FDP = fondaparinux; CPG = clinical practice guideline; GRADE = Grading of Recommendations Assessment, Development and Evaluation

* For these five indications, the review sponsor was specifically interested in the treatment/prevention of DVT; however, all recommendations that met the eligibility criteria for the treatment or prevention of VTE in general were included for all indications.

^†^CPGs must show evidence that a literature search was performed

### Data sources and searches

A literature search strategy was developed and tested through an iterative process by an experienced medical information specialist in consultation with the review team. Using the Ovid platform, CPGs were identified from Ovid MEDLINE, Ovid MEDLINE In-Process & Other Non-Indexed Citations and Embase. All searches were performed on October 26, 2015 and updated in May 2017. The 2017 update also included searches for systematic reviews and health technology assessments using the databases mentioned previously in addition to the Cochrane Library (Wiley version). The search strategies used a combination of controlled vocabulary (e.g., “Heparin, Low-Molecular-Weight”, “Venous Thrombosis”, “Perioperative Period”) and keywords, (e.g., LMWH, deep vein thrombosis, bridging). Vocabulary and syntax were adjusted across the databases. Search results were limited to those published in English and with the publication dates 2005 to present. When possible, animal-only and opinion-pieces were removed from the results. Grey literature was searched using CADTH’s Grey Matters Light [25]. Specific details regarding the database strategies appear in S2 Appendix.

### Selection of guidelines and recommendations

Two reviewers (AJ & SH) independently screened the titles and abstracts of all records returned from the literature search against the eligibility criteria outlined in our ‘Population & Clinical Areas, Interventions, Comparators, Attributes of CPGs, and Recommendation characteristics’ (PICAR) statement (**Table 1**). The same two reviewers then independently screened the full text of potentially eligible records for inclusion in ‘blocks’ by publication year (e.g., all CPGs published in 2016), beginning with the most recently published records. If supplemental documents (e.g., methodology documents presented as appendices) were available and potentially important in making a decision about a record’s eligibility, they were also retrieved and screened along with the primary guideline document. When the two reviewers agreed that at least one CPG published in each publication year block was fully eligible for inclusion, the recommendations reported within each eligible guideline were screened for inclusion and simultaneously extracted. DistillerSR online systematic review software (Evidence Partners, Ottawa, Canada) was used to manage the CPG eligibility screening and selection process. Discrepancies about inclusion were resolved through consensus.

### Data extraction

A single reviewer extracted all relevant information from CPGs into predesigned extraction forms using DistillerSR. All extracted data were then exported into Microsoft Excel 2007 (Microsoft Corporation, Redmond, WA) and independently verified for accuracy and completeness by a second reviewer. Discrepancies were resolved through consensus. General guideline information, including year of publication, developing organization and/or authors and country or countries of publication, was extracted from all included CPGs. Two reviewers (AJ & SH) independently screened each recommendation reported by included CPGs for eligibility against the PICAR statement (especially the ‘R’ element). As soon as a recommendation was deemed eligible, it was extracted by the reviewer. To facilitate data synthesis (see further details below), reviewers assigned a thematic code (e.g., ‘lactating women’) to each recommendation during data extraction. The general characteristics of each code (e.g., succinctness) were agreed upon before data extraction began. Details on the level of evidence (and strength, if provided) associated with each recommendation were also extracted.

As we did not set any *a priori* limits on the number of CPGs that were eligible for inclusion (e.g., no maximum publication year), a process was required to judge whether or not the recommendations extracted from guidelines published in (reverse) consecutive years continued to provide unique information, or they were becoming repetitive and, thus, were no longer informative. As such, we implemented a process to assess whether data saturation [26] had been met that involved regular discussion with members of the internal review team and final consultation with the larger stakeholder group.

Briefly, after a full set of recommendations were extracted (and independently verified for accuracy) from all included CPGs in each publication year, two reviewers (AJ & SH) reviewed all data collected to that point and made independent judgements about whether or not data saturation had been met for each indication. That is, each reviewer made a judgement as to whether the recommendations reported across CPGs were similar over time, or they were mostly different and, therefore, it was possible that further recommendations from additional CPGs could report unique information. When it became apparent to both reviewers that the data had become saturated for most indications, members of the internal review team provided a full set of recommendations to the broader stakeholder group for review. After consulting with the broader stakeholder group, a decision was made to stop screening CPGs for inclusion if they were published prior to 2011. Although we did not identify eligible recommendations for all indications of interest up to that point, all team members recognized that CPGs published prior to 2011 would be based on dated information and would, therefore, be of limited value.

### Guideline quality assessment

The Appraisal of Guidelines for Research and Evaluation II (AGREE II) [27] tool was used to assess the quality of included guidelines. AGREE II has been found to have high construct validity [28] and consists of 23 items arranged into six domains: scope and purpose (three items), stakeholder involvement (three items), rigour of development (eight items), clarity of presentation (three items), applicability (four items), and editorial independence (two items). Each item is scored between strongly agree (seven points) and strongly disagree (one point).

All guidelines were assessed a score for each AGREE II item on a seven-point scale by two independent reviewers (SH, AB) using a standardized form designed for use in DistillerSR. For each item assessed, reviewers provided comments justifying their decision. Scores were exported to Microsoft Excel where the percentage for each domain was calculated using the formula provided in the AGREE II user’s manual [27]:

$$\frac{\left(Obtained\;score-minimum\;possible\;score\right)}{\left(Maximum\;possible\;score-minimum\;possible\;score\right)}\;x\;100\%$$

The Seven-point AGREE II Score Calculator [29] was used to assess inter-rater reliability. The level of agreement between the two scores was assessed as ‘medium discrepancy’ if the individual appraisers’ scores for a domain were between 1.5 and 2 standard deviations (SD) from the mean domain score. A ‘high’ discrepancy was assessed when the appraisers’ scores were greater than 2 SD from the mean domain score. The reviewers independently reviewed all items for a second time if there were medium or high discrepancies within a single domain and/or when an individual item within a domain differed by three or more points. If discrepancies remained after a second review, they were resolved by a third reviewer. All AGREE II domains assessed a standardized score ≥ 60% were a considered effectively addressed, a criterion that has been applied in previously published appraisals of guidelines [30–32].

Judgements on each guideline’s overall quality were made by employing a standardized scoring rubric. Guidelines were considered to be of ‘high quality’ if they adequately addressed at least three of the six AGREE II domains, including domain three (*rigour of development*). If three or more domains were adequately addressed, except for domain three, CPGs were considered to be of ‘moderate quality’. CPGs were also assessed as being ‘moderate’ in overall quality if they adequately addressed at least two AGREE II domains, except domain three, but were assessed a score of at least 50% in that domain. All other guidelines not meeting the criteria for ‘high’ or ‘moderate’ quality were considered to be of ‘low’ overall quality.

### Data synthesis

Once all data were extracted and verified, we developed recommendation matrices in Microsoft Excel to assist us in systematically comparing, categorizing, and summarizing the nature of recommendations reported across, and within, indications. Each matrix (one for each indication) was composed of eight columns and each row contained one recommendation. The first column contained identifying information about the CPG from which the recommendation was extracted (first author/publishing organization and review identification number) and the second column contained the CPG’s publication year. In the third column, we listed the CPG’s quality (e.g. ‘high’ or ‘moderate’) as assessed using the AGREE II tool and, in the forth column, we listed the recommendation. The subsequent four columns contained 1) the recommendation’s standardized level of evidence (e.g., ‘A’), 2) its thematic code, 3) whether or not the intervention or comparator was recommended over another (‘comparative preference’), and 4) what medications were mentioned in the recommendation.

We employed thematic analysis [33] to help identify common themes across recommendations. Each recommendation matrix was used to facilitate the process. First, we isolated recommendations and their associated thematic codes so they appeared side by side. Next, one reviewer (AJ) read and reread each recommendation and its associated code to become more familiarized with the data. The reviewer then revisited and refined codes (if necessary) using an iterative process as new ideas emerged. Once completed, the revised set of codes was examined and broader patterns (i.e., themes) in the data were identified. After a final set of thematic codes was developed, they were independently reviewed and challenged by another reviewer (SH). The final set of themes was agreed upon through consensus.

Across included CPGs, a variety of systems were used to assess the quality of evidence underpinning recommendations (S1 Table). To facilitate straightforward comparisons of evidence quality between recommendations reported by different CPGs, the rating systems of all guidelines were reviewed and compared against each other. Through an iterative process of constant comparison, four standardized levels of evidence were developed and subsequently applied to each recommendation. These included: Level A: high quality evidence, level B: moderate quality evidence, level C: low quality or limited evidence, and level D: expert opinion and/or consensus panel decision. A full description of specific types of evidence (e.g., eligible study types within each level) meeting each level of evidence is provided in Table 2.

**Table 2:** Standardized levels of evidence used to inform recommendations reported by CPGs.

Level	Quality of Evidence	Criteria
**A**	**High**	Meta-analysis of multiple, well-designed, controlled trials; at least one systematic review of RCTs; at least one well-conducted randomized controlled trial
**B**	**Moderate**	Systematic review of cohort studies; at least one well conducted cohort study; at least one lower quality randomized controlled trial
**C**	**Low/limited**	Case series; poor quality cohort studies; a systematic review of case-control studies; other type of experimental study
**D**	**Expert opinion/ consensus**	No relevant evidence is available; recommendations based solely on expert opinion and/or consensus panel activities

## Results

### Literature search

**Fig 1:**
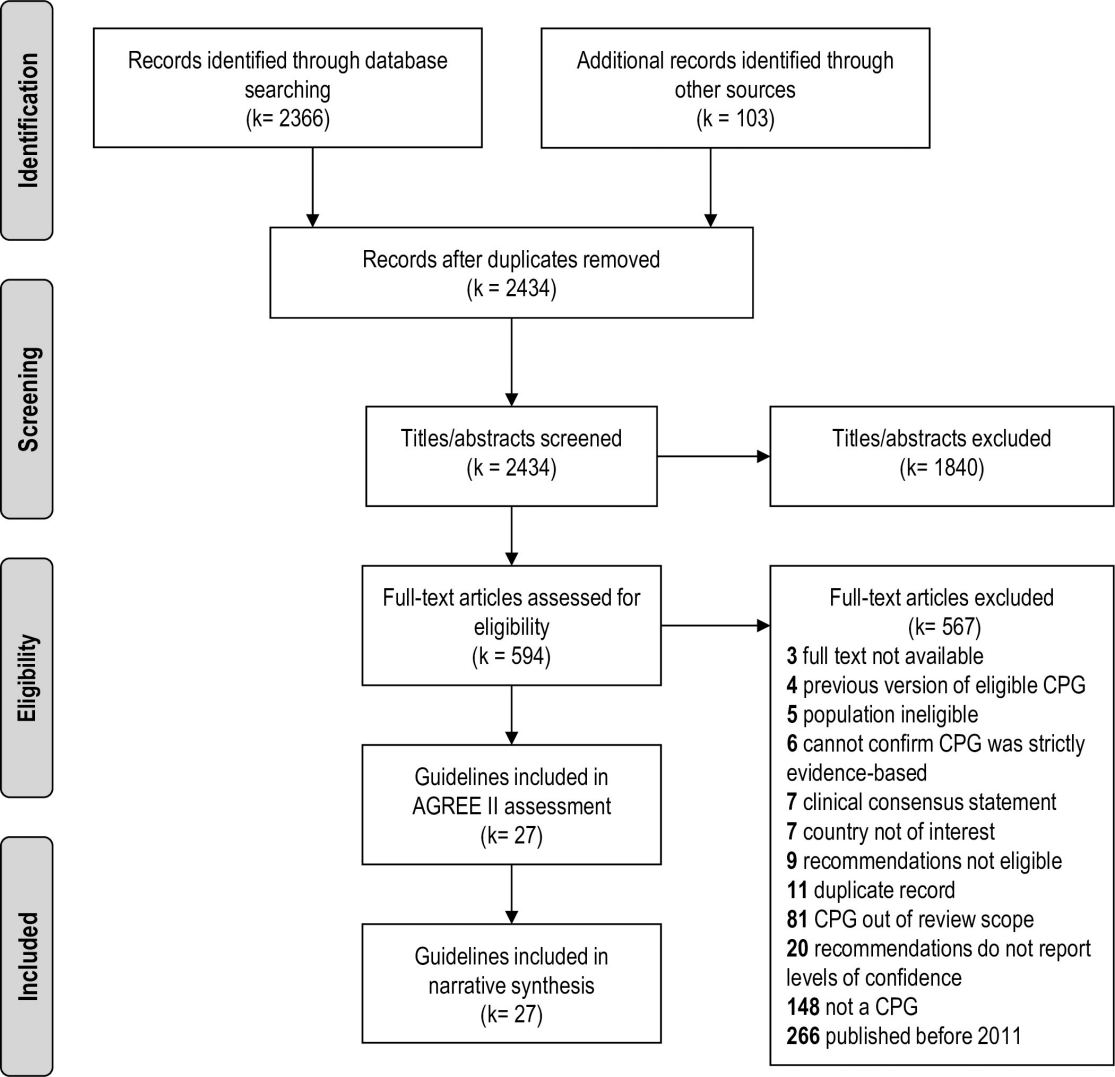
PRISMA flow diagram. A flow chart summarizing the results of the literature screening and selection process.

The results of the screening and guideline selection process have been summarized in a Preferred Reporting Items for Systematic Reviews (PRISMA) flow diagram (Fig 1). A total of 2469 records were identified from database and grey literature sources. After de-duplication and screening of titles and abstracts, 594 records were deemed potentially eligible for inclusion. During full-text screening, 567 records were excluded from further review, including 266 records published prior to 2011, leaving 27 fully eligible CPGs, which were included in the review.

### Characteristics of included guidelines

General characteristics of the 27 included CPGs are available in S2 Table. In summary, two guidelines were published in 2011 [34, 35], nine in 2012 [36–44] and 16 (59%) [5, 45–59] were published, updated, or re-affirmed between 2013 and 2017, inclusive. Just under half (k = 13 [5, 36–38, 40–44, 53, 55, 56, 58]) were developed by groups located in the United States, and an additional 13 CPGs were developed by groups based exclusively in Europe (k = 8 [34, 35, 39, 45, 50–52, 59]) and Canada (k = 5 [46–49, 57]). Additionally, one CPG [54] was developed by an international group of experts across Europe (Czech Republic, France, and the United Kingdom) and North America. None of the included CPGs originated from Japan or Australasia. Four CPGs were broad in scope [5, 37, 45, 54], addressing the management of VTE in general, whereas the others were oriented toward specific indications of interest. Thirty-three percent of included CPGs (k = 9 [34, 35, 48, 50, 52, 54–56, 58]) reported recommendations for between three and six indications of interest.

Most guidelines (63%) used the Grading of Recommendations Assessment, Development and Evaluation (GRADE) Working Group approach (k = 6 [34, 50–52, 57, 59]), or various adaptations of thereof (k = 11 [5, 36–38, 40–44, 49, 53]), to assess the quality of evidence upon which their recommendations were informed. Three CPGs [35, 39, 57] adapted assessment systems used by other groups (e.g. modified SIGN 50 and NICE rating systems [39]) and four guidelines [48, 54, 56, 58] did not identify their evidence assessment system by name. All CPGs reported the level of evidence associated with each recommendation made (e.g. high-quality systematic reviews of clinical trials). Some were explicit in reporting the strength of each recommendation (e.g. strong or weak), while in others [35, 39, 45–48, 53, 55–57], the strength of recommendations was not as well defined (e.g. “good evidence to recommend …” and “generally should be performed” [56]).

### Quality of included guidelines: AGREE II assessments

**Fig 2:**
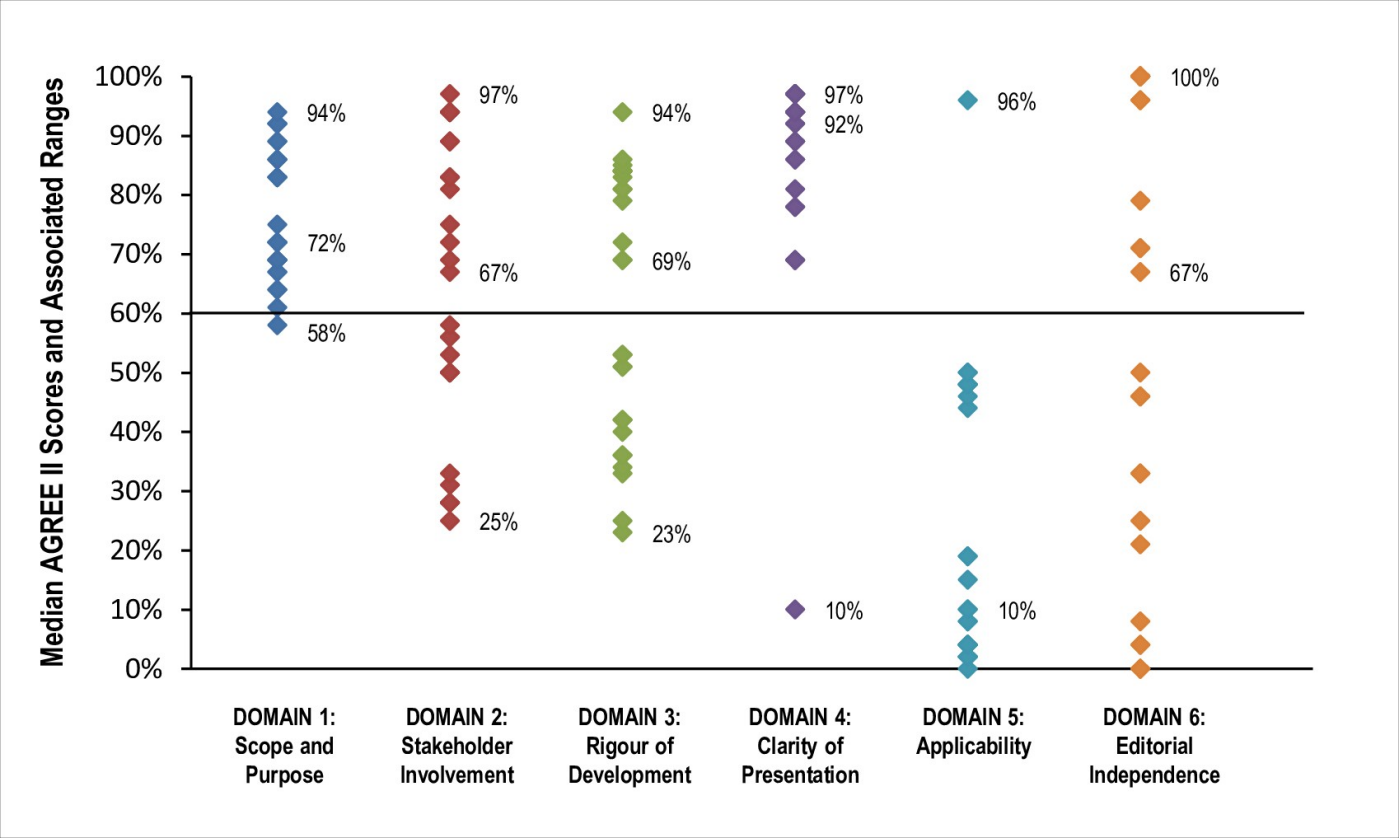
Median AGREE II domain Scores and associated ranges for all included guidelines. The horizontal line at 60% represents the cut-off score at or above which each domain was considered ‘adequately addressed’.

As shown in Fig 2, the median scores of most AGREE II domains were greater than 60 percent meaning the majority were adequately addressed by most CPGs. Domain four, *clarity of presentation*, was the best-scoring domain with all CPGs having been assessed scores greater than 60 percent (range 69 to 100 percent). Domain five (*applicability*) was the worst scoring domain, with a median score of 10 percent (range 0 to 96 percent). Nine CPGs each scored 100 percent in a single domain and eight [36–38, 40–43, 57] scored 100 percent in *editorial independence*. The median score for AGREE II domain five (*applicability*) was 10 percent; however, one CPG [44] was assessed a score of 96 percent.

Specific AGREE II scores for each domain (by CPG) are provided in S3 Table. More than half of included CPGs (k = 14) [5, 36–45, 51, 52, 58] adequately addressed at least three of six domains (including domain three, *methodological rigour*) and were judged to be of high overall quality. An additional five CPGs [47–49, 54, 55] were judged to be of moderate overall quality. Two of these [49, 55] were assessed scores of greater than 60 percent in four domains other than domain three. The remaining three CPGs [47, 48, 54] were assessed scores of greater than 60 percent in only two domains, but scored at least 50 percent in domain three. Eight CPGs [34, 35, 46, 50, 53, 56, 57, 59] judged to be of low quality were assessed scores of less than 60 percent in two or more domains and were not assessed a score at least 50 percent in domain three.

### Recommendations

#### General recommendation characteristics

We included a total of 168 recommendations on the use of LMWH (as a drug class or any specific agent) or FDP addressing nine of our ten indications of interest. We did not identify any recommendations on the use of LMWH or FDP for the post-operative prophylaxis of VTE in patients undergoing hip or knee surgery who cannot use warfarin. Across all indications, LMWH was mentioned 154 times, while specific LMWH agents (dalteparin, enoxaparin, tinzaparin, bemiparin, and nadroparin) were mentioned only 19 times and only in recommendations for patients with cancer and the prevention of VTE in patients undergoing non-orthopedic surgery. Fondaparinux was only mentioned on 35 occasions and not at all in any recommendation on the treatment of VTE in patients who cannot tolerate warfarin or in whom it is contraindicated and only once in a single recommendation on the prevention of VTE in patients with cancer. A detailed breakdown of the frequency with which each drug class and individual LMWH agents were discussed by indication is provided in S4 Table.

We identified 81 recommendations for the use of LMWH and FDP in the treatment of VTE and 87 for the prevention of VTE (including peri-operative bridging). The greatest proportion of included recommendations were related to the use of LMWH or FDP for the prevention of VTE in patients undergoing non-orthopedic surgery (r = 48), while only one recommendation addressed the treatment of VTE in patients who cannot tolerate warfarin or in whom the drug is contraindicated. A summary of recommendations for treatment-related indications is provided in Table 3, and those related to prevention have been summarized in Table 4. A full set of all included recommendations can be found in S3 Appendix.

**Table 3:** Summary of recommendations for the treatment of VTE with LMWH and FDP for five outpatient indications.

General treatment practices	Recommended pharmacologic intervention	Treatment duration and timing	Treatment cautions
**Treatment of VTE for patients without cancer: 15 Recommendations, 7 CPGs**			
- When treated with warfarin, continue LMWH for at least 5d or until optimal INR achieved (**Level C** [56]) - Start VKA therapy on d1 or d2 of treatment with LMWH (**Level A** [54]**; Level C** [37]) - Start DOACs after 1wk of anticoagulation with LMWH or FDP (**Level A** [54]; **Level B** [49])	- LMWHs for stable, low-risk, outpatients (**Level B** [56]) - Initial treatment with LMWH over FDP due to better safety outcomes (**Level B** [56]) - For patients not taking DOACs: VKAs over LMWH for long-term treatment of acute DVT of leg (**Level C** [5]) - FDP acceptable as 2^nd^ line treatment in patients with suspected HIT (**Level B** [54]); transition to VKAs in specific clinical scenarios (**Level C** [43])	- Treatment with LMWH is an acceptable alternative to VKA therapy for 3 to 6mo (**Level A** [54])	- In patients who are obese, and those with acute kidney injury or stage chronic kidney disease (stage 4 to 5), use LMWH with caution (**Level D** [45])
**Treatment of symptomatic, acute, VTE in patients with cancer: 40 Recommendations, 12 CPGs**			
- Treat with LMWH (monotherapy) over UFH for an initial 5 to 10d (**Level A** [58]); FDP is acceptable in certain clinical scenarios (**Level C** [52])	- LMWH is treatment of choice (**Level A** [47]**; Level B** [49, 52]); however, FDP may also be acceptable as an initial treatment (**Level C** [52]) - LMWHs preferred over VKAs and DOACs (**Level A** [34, 47, 58]; **Level C** [5]**; Level D** [48]) - FDP acceptable for patients with HIT (**Level A** [47]) - LMWH over VKA for patients with significant thrombocytopenia (**Level D** [47]**)**	- Treat with LMWH over VKAs for 10d to 3mo and beyond (**Level C** [52]) - Treat with LMWHs for a minimum of 3 to 6mo (**Level A** [50, 52, 54, 58]; **Level D** [48, 51]), depending on clinical scenario - LMWH acceptable for 6mo or longer in certain clinical scenarios (**Level A** [50]**; Level C** [58])	- FDP is contraindicated in patients with renal insufficiency (**Level C** [55]) - For VTE in patients with cancer and severe renal failure: treat with UFH or LMWH and monitor for anti-Xa activity (**Level A** [35]) - Treat with VKAs if LMWH is contraindicated/refused (**Level A** [47])
**Treatment of VTE for patients in whom treatment with warfarin is either not tolerated or contraindicated: 1 Recommendation, 1 CPG**			
- Treat with full-dose heparin (**Level B** [56])	- LMWH is the preferred heparin (**Level B** [56])	None reported	None reported
**Treatment of VTE for patients who have failed treatment with warfarin: 6 Recommendations, 4 CPGs**			
- Switch to LMWH (**Level C** [5, 52])	- Preferred treatment is LMWH (**Level C** [55]) - Switch to FDP in some clinical scenarios (**Level C** [48, 55])	- For cancer patients, treat with LMWH for a minimum of 4wk (**Level B** [48])	- Patients with active cancer and a history of VTE should not be switched to FDP (**Level C** [48])
**Treatment of VTE for pregnant and/or lactating females: 19 Recommendations, 6 CPGs**			
- Treat with LMWH (**Level B** [44, 56, 57])	- LMWH over VKAs and UFH (**Level B** [44, 56, 57]; **Level A** [44]) - FDP is not recommended for breastfeeding women (**Level C** [44])	- Discontinue LMWHs at least 24h prior to induction/C-section; resume no sooner than 4h after neuraxial catheter removed (**Level D** [53, 57]**; Level B** [44]) - LMWH recommended throughout pregnancy; for first 6wk postpartum (**Level B** [54])	- LMWH should be used with caution due to potentially unreliable standard or weight-adjusted dosing (**Level D** [45])

**Abbreviations:** d = day; wk = week; mo = month; DOAC = direct-acting oral anticoagulant; FDP = fondaparinux; HIT = heparin-induced thrombocytopenia; INR = international normalized ratio; LMWH = low molecular weight heparin; VKA = vitamin K antagonist; VTE = venous thromboembolism

**Levels of Evidence:** Level A: high-quality evidence, level B: moderate-quality evidence, level C: low/limited-quality evidence, level D: Expert opinion/consensus

**Table 4:** Summary of recommendations related to the prevention of VTE with LMWH and FDP for four outpatient indications.

General prophylactic approaches	Recommended pharmacologic intervention	Duration and timing of prophylaxis	Treatment cautions
**Post-operative prophylaxis of VTE for patients undergoing orthopedic surgery of the lower limbs: 12 Recommendations, 2 CPGs**			
- Begin LMWH ≥ 12hr preoperatively, or ≥ 12hr postoperatively **(Level B** [42]) - Begin FDP at least 6 to 8hr after surgery **(Level B** [54])	- LMWH or FDP acceptable (**Level A** [54]; **Level B** [42]) - LMWH over FDP for patients undergoing HFS, THA, or TKA (**Level B** [42])	- Continue LMWH or FDP for a minimum of 10 to 14d **(Level B** [42]), but preferably 4 to 6wk **(Level A** [54]**; Level B** [54]) - LMWH either pre or post operatively (**Level A** [54]) and until full ambulation in specific clinical circumstances (**Level B** [54])	None reported
**Prevention of VTE for non-orthopedic surgical patients: 48 Recommendations; 8 CPGs**			
- High-risk plastic surgery: start LMWH or FDP 24hr post-operatively **(Level B** [54]) - Patients with cancer undergoing ‘other surgeries’ (and are not at high risk for serious bleeding) should receive prophylactic LMWH or UFH (**Level A** [59])	- Insufficient evidence to support post-operative FDP over LMWH in cancer surgery **(Level C** [52]) - FDP for patients undergoing abdominal-pelvic surgery and LMWH/UFH is contraindicated and not at high risk for major bleeding **(Level C** [40]) - LMWH or FDP over UFH in high-risk patients undergoing major surgery for benign disease **(Level A** [54])	- Cancer patients: post-operative LMWH or FDP for at least 7 to 10d **(Level A** [39]; **Level A** [52]**),** but preferably extended to 4 wk **(Level A** [35, 54, 58], **Level B** [40]**; Level C** [52]; **Level B** [54]) - Patients undergoing major gynecological cancer surgery with other risk factors: continue prophylaxis with LMWH for 4wk (**Level C** [59])	- No data to support any one LMWH over another for post-operative prophylaxis in cancer patients **(Level C** [52]) - After bypass surgery, further treatment with LMWH is not recommended (**Level B** [45])
**Prevention of VTE for patients with cancer: 19 Recommendations; 9 CPGs**			
- LMWH should be used selectively for outpatients with solid tumors who received chemotherapy (**Level B** [58]) -High risk patients receiving highly thrombotic antiangiogenic therapy should receive LMWH (**Level B** [35, 58]**; Level C** [39, 55, 59]) - Cancer patients with liver disease can receive LMWH (**Level C** [47])	- No single LMWH preferred over another for elderly patients or those with solid tumors and additional risk factors for VTE (**Level B** [41, 46]) - For patients with impaired renal function, enoxaparin might be less preferable to tinzaparin and dalteparin **(Level B** [46]) - Cancer patients requiring therapy can be prophylaxed with LDUH or LMWH (enoxaparin or dalteparin); FDP can be used as an alternative (**Level A** [54])	- LMWH for 6mo for patients receiving chemotherapy in an adjuvant setting **(Level A** [35]) - LMWH (enoxaparin or dalteparin) for 6 to 14d for patients with cancer that require therapy (**Level A** [54])	- Prophylaxis with LMWH not recommended for patients with no additional risk factors, including patients with CVCs **(Level D** [46]; **Level B** [41]) - Nadroparin should not be taken for >4mo in patients with lung or gastrointestinal cancer **(Level A** [39])
**Peri-operative bridging for patients who require long-term warfarin and must discontinue due to surgery: 8 Recommendations; 4 CPGs**			
- To avoid hospitalization, bridging therapy with LMWH should be done in an outpatient setting (**Level B** [54]**)** - Until stable on VKA therapy, patients with mechanical heart valves should receive bridging therapy (prophylactic dose) with UFH or LMWH instead of therapeutic dose UFH (**Level C** [36]**)**	- LMWH over UFH, especially to avoid hospitalization **(Level B** [54]) - Patients with VTE >3 mo earlier: LMWH (prophylactic dose) instead of bridging therapy **(Level C**[34]) - Patients with a mechanical heart valve should receive bridging therapy with LMWH over UFH in certain clinical scenarios (**Level B** [54]**)**	- Stop LMWH ~24hr prior to surgery (**Level C** [38]) - For high-bleeding risk surgery, resume LMWH 48 to 72hr after surgery **(Level C** [38]; **Level B** [54]**)** - For minor surgery, resume LMWH within 24hr after surgery if adequate haemostasis achieved **(Level B** [54])	None reported

**NB:** We did not identify recommendations for the post-operative prophylaxis of VTE for patients undergoing hip or knee surgery who cannot use warfarin

**Abbreviations:** d = day; hr = hour; wk = week; mo = month; CVCs = central venous catheters; FDP = fondaparinux; HFS = hip fracture surgery; LDUH = low dose unfractionated heparin; LMWH = low molecular weight heparin; THA = total hip arthroplasty; TKA = total knee arthroplasty; UFH = unfractionated heparin; VTE = venous thromboembolism

**Levels of Evidence:** Level A: high-quality evidence, level B: moderate-quality evidence, level C: low/limited-quality evidence, level D: Expert opinion/consensus

As summarized in Tables 3 and 4, across all indications, LMWH was often recommended as the preferred anticoagulant for treating and preventing VTE in the outpatient setting. For the treatment of VTE in pregnant and/or lactating women, three CPGs recommended the use of LMWH over other heparins [44, 56, 57] or VKAs [44] in eight recommendations. For the treatment of VTE in patients with cancer, some CPGs mentioned the use of specific LMWH agents. For example, one CPG [47] advised that, for patients with impaired renal function/insufficiency, tinzaparin and dalteparin might be better treatment choices than enoxaparin (evidence Level B). Another CPG [55] recommended that dalteparin be considered for the treatment of VTE in patients with cancer because “it is the only LMWH approved by the FDA for this indication” (evidence level C). For the prevention of VTE in patients with cancer, one CPG [46] advised that, for patients with impaired renal function, tinzaparin and dalteparin may be more preferable to enoxaparin (evidence Level B) and another [54] recommended that patients with active cancer requiring therapy should be prophylaxed with enoxaparin or dalteparin for six to 14 days (evidence Level A).

While FDP was recommended as a potential treatment option for six indications, it was never recommended as a first line of treatment, and its use was discouraged or cautioned in a variety of specific clinical scenarios (Tables 3 and 4). For example, one CPG [44] recommended that health practitioners use alternatives to FDP for the treatment of VTE in breastfeeding women (evidence level C). For patients with active cancer who fail treatment with warfarin, another CPG [55] recommended that LMWHs should be used over FDP (evidence level C), and yet another [48] explicitly recommended against a switch to FDP in patients with active cancer who fail treatment with either LMWH or warfarin (also evidence level C). For the treatment of VTE in patients without cancer, DOACs were recommended over FDP (evidence level B) by one CPG [54], which also recommended that FDP be used only as an alternative to LMWHs for the prevention of VTE in patients with cancer (evidence level A).

As summarized in Table 5, the levels of evidence underpinning recommendations varied widely within, and across, indications. Overall, more than half of included recommendations (64%) were based on moderate-to low- quality/limited evidence (level C) and only eight percent of recommendations were based solely on expert consensus/expert opinion (level D). The remaining 29% of recommendations were based on high-quality evidence (level A). More specifically, the only included recommendations based solely on evidence Level D were for the use of LMWH or FDP in 1) the treatment of VTE in pregnant or lactating women, 2) symptomatic, acute, VTE in patients with cancer, and 3) the prevention of VTE in patients with cancer. One third of included recommendations for the treatment of symptomatic, acute, VTE in patients with cancer were based on evidence Level A; however, more than half (53%) were based on the lowest levels of evidence (Level C, 30% or Level D, 23%). All recommendations for the use of LMWH or FDP in the setting of peri-operative bridging were informed by evidence level B (moderate-quality).

**Table 5:** Number of clinical practice guidelines reporting recommendations by quality type recommendations reported by indication.

Indication	# RECs reported by level of evidence*	# of CPGs reporting any REC by quality^¥^
**Treatment of:**		
VTE in patients in whom treatment with warfarin is either not tolerated or contraindicated	**Level A:** 0 | **Level B:** 1 | **Level C:** 0 | **Level D:** 0	**High:** 0 | **Moderate:** 0 | **Low:** 1
VTE in patients who fail treatment with warfarin	**Level A:** 0 | **Level B:** 1 | **Level C:** 5 | **Level D:** 0	**High:** 2 | **Moderate:** 2 | **Low:** 0
VTE in patients without cancer	**Level A:** 3 | **Level B:** 5 | **Level C:** 7 | **Level D:** 0	**High:** 4 | **Moderate:** 2 | **Low:** 1
VTE in pregnant or lactating women	**Level A:** 3 | **Level B:** 9 | **Level C:** 4 | **Level D:** 3	**High:** 3 | **Moderate:** 1 | **Low:** 3
Symptomatic, acute, VTE in patients with cancer	**Level A:** 13 | **Level B:** 6 | **Level C:** 12 | **Level D:** 9	**High:** 4 | **Moderate:** 5 | **Low:** 3
**Prevention of:**		
VTE in patients with cancer	**Level A**: 4 | **Level B:** 9 | **Level C**: 5 | **Level D:** 1	**High:** 3 | **Moderate:** 3 | **Low:** 3
VTE in non-orthopedic surgical patients	**Level A:** 20 | **Level B:** 19 | **Level C:** 9 | **Level D:** 0	**High:** 5 | **Moderate:** 1 | **Low:** 2
**Post-operative prevention of:**		
VTE in patients undergoing orthopedic surgery of the lower limbs	**Level A**: 5 | **Level B**: 7 | **Level C**: 0 | **Level D**: 0	**High:** 1 | **Moderate:** 1 | **Low:** 0
VTE in patients undergoing hip or knee surgery who cannot use warfarin	No recommendations identified	No recommendations identified
**Peri-operative bridging in:**		
patients who require long-term warfarin and must discontinue due to surgery	**Level A**: 4 | **Level B**: 4 | **Level C**: 0 | **Level D**: 0	**High:** 2 | **Moderate:** 1 | **Low:** 1

**Abbreviations:** CPG = clinical practice guideline; REC = recommendation; VTE = venous thromboembolism

*Levels of Evidence: Level A: High-quality evidence, Level B: Moderate-quality evidence, Level C: Low/limited-quality evidence, Level D: Expert opinion/consensus

^**¥**^**Overall Guideline Quality: High** = at least three of six AGREE II domains (including domain 3) scored earned scores of >60%; **Moderate** = three or more AGREE II domains earned scores of >60%, except for domain 3 OR at least two AGREE II domains earned scores of >60%, except for domain 3, which earned a score of at least 50%; **Low** = any guideline not meeting the criteria for high or moderate quality.

As shown in Table 5, the proportion of high-, moderate-, and low-quality CPGs reporting recommendations of interest varied across clinical scenarios. Specifically, more than half (63%) of the CPGs reporting recommendations on the use of LMWH or FDP for the prevention of VTE in patients undergoing non-orthopedic surgery were judged to be of high overall quality [39, 40, 45, 52, 58]. In comparison, no high-quality CPGs reported recommendations for the use of LMWH or FDP in the treatment of VTE in patients unable to tolerate warfarin or in whom it is contraindicated. Finally, just under half of the CPGs reporting recommendations on the treatment of symptomatic, acute, VTE in patients with cancer (41%) [47–49, 54, 55] were judged to be of moderate overall quality.

There was no apparent correlation between the quality of evidence underpinning recommendations on the use of LMWH or FDP in treating or preventing VTE and the quality of CPGs reporting them (see S4 Appendix). For example, while no recommendations for the treatment of VTE in patients without cancer were based on evidence Level D, high-quality CPGs reported the largest proportion of Level C recommendations for this indication. For the treatment of VTE in patients with cancer, all recommendations reported by low-quality CPGs were based on evidence level A or B. In comparison, all recommendations for the post-operative prophylaxis of VTE in patients undergoing hip or knee surgery that were based on evidence level A were reported by moderate-quality CPGs. Additionally, all high-quality CPGs reporting recommendations for this indication were based on evidence Level C.

#### Emergent themes

During data collection and synthesis, we noted a variety of recurring themes in the content of included recommendations. A word cloud (www.wordle.com/create) showing the relative frequencies of key recommendation themes is provided in S1 Fig. To briefly summarize, the most prolific theme was the use of LMWH or FDP to treat or prevent VTE within the clinical context of cancer. While two indications were specifically focused on patients with malignancy, more than two-thirds (70%) of recommendations for the prevention of VTE in non-orthopedic surgical patients were focused on cancer surgery. One additional recommendation for the treatment of VTE in patients who fail warfarin also focused on patients with cancer. Another frequently noted theme was the use of LMWH or FDP for the prevention of VTE in patients undergoing abdominal and/or pelvic surgeries. With regard to recommendations for patients undergoing orthopedic surgery, surgeries involving the hip (e.g., total hip replacement and hip fracture surgery) were discussed the most. Additional themes included a focus on high-risk patients/clinical situations, recommended duration of anticoagulation (e.g., anticoagulate for three to six months), as well as the importance of considering patients’ renal function, age, and weight when making decisions on anticoagulant use.

## Discussion

This is the first SR of CPGs to comprehensively summarize recommendations on the outpatient use of LMWH and FDP in the treatment and prevention of VTE to inform clinical prescribing. The largest proportion of recommendations identified were for the prevention of VTE in patients undergoing non-orthopedic surgery and, across indications, ‘cancer’ was the most widely discussed clinical scenario. More than half were informed by moderate- to low-quality/limited evidence. We did not identify any recommendations for the use of LMWH or FDP in the post-operative prophylaxis of VTE in patients undergoing hip or knee surgery who cannot use warfarin. The majority of included CPGs were judged to be of moderate to high overall quality.

### Quality of CPGs informing the review

We used a widely accepted and validated tool (AGREE II [27]) to assess the overall quality of included CPGs [60]. While we were able to broadly distinguish CPGs by quality (high, moderate, and low) using AGREE II, we are cognizant that this tool was designed to assess the quality of guideline reporting and process, not their clinical content or the quality of evidence underpinning the recommendations they report [60]. Thus, while individual domain scores were useful for comparing individual CPGs against each other, we hesitate to interpret CPGs assessed as being of ‘low’ overall quality as ‘bad’ and those assessed as being of ‘high’ overall quality CPGs as ‘good’. Indeed, CPGs reporting little detail about their development processes should not automatically be considered of limited clinical use because AGREE II is not useful in informing on neither the clinical credibility, nor the implementability, of included recommendations [61]. To address these shortcomings, members of the AGREE working group have developed AGREE-REX (Recommendations Excellence) [62], a recommendation evaluation tool that is currently in the late stages of validation. This new tool is designed to guide researchers in developing, reporting, and evaluating the clinical relevance and implementability of guideline recommendations. As such, future SRs of CPGs that aim to evaluate the quality of CPG recommendations should consider using this tool as a complement to AGREE II.

The majority of included CPGs earned moderate to high-quality overall ratings according to our assessment rubric; however, only one [45] was judged to have adequately addressed every domain. Across other included CPGs, while domains one (*scope and purpose*) and four (*clarity of presentation*) were particularly well-addressed, domain five (*applicability*), which addresses factors that may impact a guideline’s implementation, potential impact on resources, and strategies to improve uptake, was very poorly addressed. This finding is not unique to this review, rather, it appears to be a general criticism of many reviews of CPGs published on a variety of topics (e.g., breast cancer [63], osteoarthritis [64], and childhood fever [65]). Given that many factors influence the contextualization of scientific evidence for real-world application (i.e., available resources, cost-effectiveness, and availability of services [66]), future CPGs (and CPG updates) should consider using a validated framework, such as ADAPTE [67], to systematically integrate important cultural and organizational contexts during development. Finally, while just under one third of CPGs were judged to be of ‘low-quality’, all included guidelines were explicitly evidence-based and these rating should not be interpreted as a direct reflection of their clinical usefulness.

A key element of evidence-based CPGs is the systematic identification, and formal appraisal, of research evidence [68]. Indeed, recommendations based on low-quality/limited evidence or expert opinion are more susceptible to bias than those built on higher-quality evidence. Thus, CPG users should be aware of the quality of evidence informing them [68, 69]. A variety of assessment frameworks can be used to evaluate evidence quality, many of which use various combinations of numbers and letters to report their findings [68, 69]. If developers are transparent about what these numbers and letters signify, the exact type of validated evaluation framework used will likely not matter to the end user. For those who wish to compare recommendations reported across CPGs, such as in this review, different appraisal systems can lead to confusion as the same evidence can be rated differently depending on which assessment system is adopted [68–70].

### Quality of evidence underpinning CPG recommendations

We created a standardized evidence quality rubric to compare and contrast the quality of evidence underpinning recommendations reported across CPGs; however, this system still presented us with certain challenges. First, while many included CPGs used the GRADE system (or various adaptations thereof) to assess evidence quality, others used systems that were quite different in terms of process and reporting. Thus, standardization required some subjectivity on our part and our rubric was heavily influenced by the GRADE approach. Second, while many CPGs presented the same general recommendation for a specific clinical scenario (e.g., LMWH should be used to treat VTE in patients with cancer), they often provided a variety of additional details (e.g., a specific duration of treatment) and the strength of evidence provided was particularly influenced by presence of the additional details. Encouragingly, guideline development organizations and academic journals have started to adopt and promote the use of a single transparent framework (GRADE) [68, 71], which will facilitate direct comparisons of evidence quality across CPGs without the need to standardize.

When reported, we collected information on the strength of recommendations (i.e., “the extent to which one can be confident that adherence to the recommendation will do more good than harm”[68]); however, this information was not considered during data synthesis. Even though we chose to interpret and report our findings strictly on the basis of evidence quality, we do understand the value that this type of clinical input brings to recommendations. Indeed, recommendations based on the highest-quality evidence are not guaranteed to result in successful outcomes in all cases. It is also quite possible that only low-quality or limited evidence is available to inform recommendations in certain clinical situations. Ultimately, while the level of evidence upon which recommendations are developed is important, decisions on, and judgements about, patient care must ultimately be made by the most appropriate health care professional in consultation with patients among whose values and treatment preferences may differ [72].

### Recommendations on the use of specific LMWH agents

Across recommendations, LMWH was most frequently discussed in terms of a drug class, although comparatively few mentioned specific uses for individual LMWH agents. When specific LMWHs were discussed in recommendations, they were based on very low-quality/limited evidence. These findings highlight substantial limitations on the availability of evidence for the use of LMWHs as a whole. As noted by Merli et al. [73], although LMWHs have similar class effects, each agent has a distinct pharmacologic and biochemical profile. Indeed, therapeutically equivalent doses of LMWHs have not yet been established making product substitution difficult, especially within the context of renal impairment [73]. Furthermore, although FDP is sometimes referred to as an “ultra” LMWH, it has a distinct chemical structure and should also not be considered interchangeable with LMWHs [73]. Several CPGs recommended LMWH as the preferred anticoagulant class over alternative medications (e.g., LMWHs are favored over UFHs because their pharmacokinetic profile is more predictable, they have improved bioavailability, and are easier to use [73, 74]); however, broad generalizations such as these provide somewhat limited guidance as specific LMWHs may in fact be more preferable to others in specific situations. Owing to this scarcity of evidence, further studies should be conducted to establish the comparative efficacy and safety of specific LMWHs against each other and FDP across clinical scenarios.

### Implications for formulary listing and clinical practice

These findings contributed to the recommendation that an outpatient formulary listing for LMWH and FDP become streamlined to include coverage for the following indications: acute treatment of VTE in patients without cancer; acute treatment, and secondary prophylaxis, of VTE in patients with cancer; treatment and prophylaxis of VTE in pregnant or lactating women; post-operative prophylaxis of VTE in patients a) undergoing surgery of the lower limbs and b) patients undergoing non-orthopedic surgery who are at high risk for thromboembolic complications; and peri-operative bridging in patients who require long-term therapy with warfarin [16]. The paucity of evidence on the comparative efficacy and safety of individual LMWH agents precluded our ability to make recommendations for the use of specific medications across indications. Although coverage for LMWHs and FDP was only recommended for patients meeting these specific clinical circumstances, the available evidence from CPGs suggests that indications outside these (e.g., FDP for pregnant patients with a history of HIT who require anticoagulation) would be uncommon. For these types of exceptional circumstances, coverage could be sought using the appropriate channels external to the outpatient formulary [16].

A streamlined formulary listing for LMWH and FDP is expected to reduce clinician burden in prescribing, and improve patient access to, these medications. As noted; however, a variety of factors influence prescriber decision-making, many of which fall beyond the scope of a single outpatient formulary listing [13–16]. For example, dosing LMWHs can be complicated, especially for treatment-related indications as they are dependent upon patient weight, which can sometimes be difficult to estimate correctly (e.g., in patients who are morbidly obese) [75]. Furthermore, because every LMWH has a distinct pharmacologic and biochemical profile, dosing is not interchangeable across agents [73]. As such, clinicians may choose to prescribe an agent with which they are more familiar, regardless of the indication for treatment. Although there is a paucity of clear evidence about the comparative efficacy of LMWH agents and FDP across specific clinical scenarios, evidence suggests that LMWHs are not necessarily clinically equivalent [76]. Thus, if clinicians make prescribing decisions based on the assumption that LMWH agents are equivalent, patients may not be prescribed the ‘best’ treatment for their clinical situation.

Although this review focused on outpatient indications for LMWH and FDP, it should be noted that most patients begin treatment with these medications in-hospital and continue after discharge [77]. As such, hospital-based prescribers often attempt to align inpatient prescriptions so that coverage for the same agent will apply upon discharge [76]. This can be time consuming; however, as it requires that prescribers become familiar with coverage offered across a variety of prescription drug plans that may have variable listings [78]. Likewise, community physicians have cited attempts to align prescriptions for specific LMWH agents with those of hospitals in close proximity to “minimize confusion” if a patient is admitted [76]. Prescribers may also choose to prescribe the lowest cost LMWH, or substitute a higher cost agent for one that costs less. These practices also fall beyond the scope of influence of a single formulary listing and, for the reasons previously described, may result in a less-than-optimal anticoagulant being prescribed to patients [73, 76].

Ultimately, decisions about patient care must be made by competent health care providers in consultation with patients whose values and treatment preferences may differ [72]. For many patients, the route of administration of LMWH and FDP, subcutaneous injection, is burdensome; a fact that will not change with an improved outpatient formulary listing. As such, regardless of their clinical advantages over other anticoagulants, some patients will refuse to be treated with them. For patients who agree to self-injection, but do not have access to pre-filled syringes, unnecessary burden may result from difficulties in drawing up correct doses from multi-dose vials [76]. It is clear, therefore, that patient-specific factors, such as access to easy-to-use supplies and appropriate support and education around self-injection also need to be addressed to ensure that optimal outpatient patient care with LMWH and FDP is achieved.

### Strengths and limitations

We conducted a rigorous SR of CPGs that enabled the development of a robust evidence base on the recommended uses of LMWHs and FPD across several outpatient indications of specific interest to clinicians and decision-makers. Because this study was specifically designed to produce evidence for policy decision-making, it was carried out in a hastened timeframe, and certain constraints were placed on inclusion criteria and methodological processes. These limits were, however, balanced against the broad inclusion of explicitly evidence-based CPGs developed by organizations located in a broad range of geographical locations. We evaluated the quality of CPGs using AGREE II, a validated international CPG quality assessment tool, and our use of the Seven-point AGREE II Score Calculator (with the built-in concordance calculator) facilitated efficient inter-rater comparisons and helped us quickly identify important discrepancies. Although AGREE II assessments were completed by two raters instead of the preferred four, even when inter-rater discrepancies were ‘low’ (as judged by Seven-point Score Calculator), both raters independently re-evaluated all domain item scores that differed by three points or greater. In doing so, we feel that additional reviewers would not likely impact overall scores. Importantly, while we made our best efforts to identify supplemental documents that could possibly have been useful during AGREE II assessments, we cannot rule out the possibility that some may have been missed. Furthermore, while our standardized evidence quality rubric allowed us to compare and contrast the quality of evidence underpinning recommendations reported across CPGs, we were unable to formally evaluate the clinical credibility and implementability of included recommendations.

## Conclusion

Drug formularies play an important role in allowing patients access to required medications; however, they need to be updated as new evidence emerges. We conducted a SR of CPGs to determine whether the recommendations they reported aligned with the indications for the use of LMWH and FDP in an outpatient drug formulary. As a drug class, LMWH was widely recommended as safe and efficacious for the treatment and prevention of VTE in outpatients. These findings contributed to the recommendation that the outpatient formulary listing for LMWH and FDP be modernized and streamlined to include coverage for specific outpatient indications. A notable paucity of evidence; however, precluded our ability to make recommendations on the use of individual agents. Given the distinct pharmacologic and biochemical profiles of each LMWH and FDP, further studies should be conducted to establish the comparative efficacy and safety of individual agents against each other and FDP across clinical scenarios. Once available, this information should be incorporated into CPGs to ensure that the most up to date evidence is considered by clinicians during the prescription decision-making process. This information will also be informative for researchers who wish to contribute to drug class reviews on these medications as new evidence emerges, and for decision-makers who will need to consider new evidence during future formulary reviews and updates on this drug class.

## Supporting information

S1 Appendix PRISMA 2009 checklist. (PDF)

S2 Appendix Initial & updated search strategies. (PDF)

S3 Appendix Recommendations matrices reporting all included recommendations. (PDF)

S4 Appendix Summary of treatment and prevention-related recommendations by the level of evidence underpinning them and quality of CPG from which they originated. (PDF)

S1 Table Evidence assessment scales used by all included clinical practice guidelines. (PDF)

S2 Table General characteristics of included clinical practice guidelines. (PDF)

S3 Table Mean (SD) AGREE II scores by domain and overall guideline quality assessment results. (PDF)

S4 Table Number of recommendations, the quality of CPGs reporting them, and the frequency with which each drug class and individual LMWH agents were discussed, by indication. (PDF)

S1 Fig A word cloud showing the most common themes associated with included recommendations. (EMF)
