# Supplementary material for: A systematic review of clinical practice guidelines on the use of low molecular weight heparin and fondaparinux for the treatment and prevention of venous thromboembolism: Implications for research and policy decision-making
Source: PLoS One. 2018 Nov 9;13(11):e0207410. doi: 10.1371/journal.pone.0207410 (PMC6226206; doi:10.1371/journal.pone.0207410)
Supplement: S2 Appendix — (PDF) [file pone.0207410.s002.pdf]

LWMH Indications – Guidelines

2015 Oct 26

OVID

Database: Ovid MEDLINE(R) In-Process & Other Non-Indexed Citations and Ovid MEDLINE(R) <1946 to Present>, Embase <1980 to 2015 Week 43>

Search Strategy:
----
1 exp Heparin, Low-Molecular-Weight/ (57421)
2 LMWH.tw,kw. (10261)
3 ((low molecular weight or LMW) adj1 heparin).tw,kw. (21122)
4 Heparin, Low-Molecular-Weight.rn. (7336)
5 (Dalteparin* or FR-860 or Fragmin or Fragmine or Kabi-2165 or "K 2165'" or K2165 or Tedelparin or low liquemin).tw,kw. (4352)
6 dalteparin.rn. (818)
7 (Enoxaparin* or Clexan* or EMT-966 or EMT-967 or HSDB 7846 or Klexane or Lovenox or PK10169 or PK 10169 or "PK-10,169" or RP 54563 or UNII-8NZ41MIK1O).tw,kw. (11474)
8 enoxaparin.rn. (19090)
9 (nadroparin* or CY 216 or CY 216d or CY216 or CY216d or Fraxiparin* or LMF CY-216 or Nadroparin Calcium or Nadroparine or Nadrohep or Fraxodi or Seleparin* or Tedegliparin*).tw,kw. (2786)
10 nadroparin.rn. (4305)
11 (tinzaparin* or Innohep or UNII-7UQ7X4Y489).tw,kw. (1357)
12 tinzaparin.rn. (271)
13 (bemiparin* or hibor or phivor or ardeparin* or UNII-N3927D01PB).tw,kw. (343)
14 (certoparin* or Alphaparin* or Alpha-parin* or Mono-Embolex or Monoembolex).tw,kw. (370)
15 (Reviparin* or Clivarin* or LU 47311 or LU47311 or lomorin).tw,kw. (510)
16 reviparin.rn. (87)
17 (parnaparin* or parvoparin* or fluxum or lohepa or lowhepa or minidalton or op 2123 or CB-01-05-MMX).tw,kw. (258)
18 Parnaparin.rn. (35)
19 (semuloparin* or mulsevo or visamerin or AVE 5026 or AVE5026 or UNII-4QW4AN84NQ).tw,kw. (117)
20 semuloparin.rn. (11)
21 sevuparin*.tw,kw. (8)
22 sevuparin.rn. (0)
23 (ardeparin* or normifio or normiflo or rd heparin or wy 90493 or wy90493).tw,kw. (203)
24 ardeparin.rn. (22)
25 (adomiparin* or "m 118" or m118).tw,kw. (164)
26 adomiparin.rn. (0)
27 ("cy 222" or cy222).tw,kw. (224)
28 cy 222.rn. (167)
29 (danaproid or "kb 101" or kb101 or lomoparan or lomoparin or mucoglucuronan or org 10172 or org10172 or organan).tw,kw. (1751)
30 danaproid.rn. (420)
31 deligoparin*.tw,kw. (3)
32 deligoparin.rn. (0)
33 ((heparin adj1 dihydergot) or (dihydroergotamine adj1 heparin) or Embolex or (heparin adj1 DHE)).tw,kw. (433)
34 heparin-dihydergot.rn. (75)
35 idrabiotaparinux.tw,kw. (80)

36 idrabiotaparinux.rn. (109)
37 idraparinux.tw,kw. (316)
38 idraparinux.rn. (690)
39 livaraparin calcium.tw,kw. (0)
40 livaraparin calcium.rn. (0)
41 minolteparin*.tw,kw. (0)
42 minolteparin.rn. (0)
43 rd 11885.tw,kw. (13)
44 rd 11885.rn. (0)
45 tafoxiparin*.tw,kw. (3)
46 tafoxiparin.rn. (1)
47 (fondaparinux or arixtra or quixidar or xantidar or "Org 31540" or "SR 90107" or "SR 90107A" or "UNII-X0Q6N9USOZ" or "UNII-J177FOW5JL").tw,kw. (4212)
48 (fondaparinux or fondaparinux sodium).rn. (6004)
49 or/1-48 (66190)
50 Venous Thrombosis/ (44375)
51 Upper Extremity Deep Vein Thrombosis/ (788)
52 (deep adj (venous or vein$1 or vena) adj2 thrombos*).tw,kw. (50364)
53 (("deep venous" or "deep vein") adj2 (thrombus or thrombophlebitis or "thrombo-phlebitis")).tw,kw. (571)
54 (DVT or DVTs).tw,kw. (20654)
55 Venous Thromboembolism/ (29282)
56 ((venous or vein$1 or vena) adj2 (thromboemboli* or thrombo-emboli*)).tw,kw. (39299)
57 (VTE or VTEs).tw,kw. (18180)
58 exp Perioperative Care/ (162408)
59 exp Perioperative Period/ (90231)
60 (peri-operative or perioperative).tw,kw. (157153)
61 (bridge or bridging).tw,kw. (139904)
62 (bridg* adj3 (anti-coagulation or anticoagulation)).tw,kw. (371)
63 (interrupt* adj3 warfarin).tw,kw. (277)
64 exp Venous Thromboembolism/pc (16994)
65 ((prevent* or prophyla* or chemoprophyla* or chemo-prophyla*) adj3 (thromboemboli* or thrombo-embolic* or thrombos#s or VTE or VTEs)).tw,kw. (28336)
66 (thromboprophyla* or thrombo-prophyla*).tw,kw. (8927)
67 Postoperative Complications/pc (56418)
68 or/50-67 (646872)
69 49 and 68 (28651)
70 exp Animals/ not (exp Animals/ and Humans/) (9247035)
71 69 not 70 (27049)
72 (comment or editorial or interview or letter or news).pt. (3098954)
73 71 not 72 (25112)
74 (guideline or practice guideline or consensus development conference or "consensus development conference, NIH").pt. (35271)
75 exp Clinical Protocol/ (209994)
76 Critical Pathways/ (11835)
77 (guideline* or standards or consensus* or recommendat* or practice parameter* or position statement* or policy statement* or CPG or CPGs or best practice*).ti. (276656)
78 (care adj3 (path or paths or pathway or pathways or map or maps or plan or plans or standard or standards)).ti. (14909)
79 ((critical or clinical or practice) adj3 (path or paths or pathway or pathways or protocol)).ti. (6518)
80 (algorithm* adj3 (pharmacotherap* or pharmaco-therap* or chemotherap* or chemo-therap* or chemotreatment* or chemo-treatment* or therap* or treatment* or intervention*)).ti. (2478)

81 clinical algorithm*.ti. (385)
82 or/74-81 (522059)
83 73 and 82 (1237)
84 limit 83 to yr="2005-current" (977)
85 limit 84 to english language (865)
86 85 use prmz (223)
87 exp low molecular weight heparin/ (57421)
88 LMWH.tw,kw. (10261)
89 ((low molecular weight or LMW) adj1 heparin).tw,kw. (21122)
90 (Dalteparin* or FR-860 or Fragmin or Fragmine or Kabi-2165 or "K 2165'" or K2165 or Tedelparin or low liquemin).tw,kw. (4352)
91 (Enoxaparin* or Clexan* or EMT-966 or EMT-967 or HSDB 7846 or Klexane or Lovenox or PK10169 or PK 10169 or "PK-10,169" or RP 54563 or UNII-8NZ41MIK1O).tw,kw. (11474)
92 679809-58-6.rn. (8044)
93 (nadroparin* or CY 216 or CY 216d or CY216 or CY216d or Fraxiparin* or LMF CY-216 or Nadroparin Calcium or Nadroparine or Nadrohep or Fraxodi or Seleparin* or Tedegliparin*).tw,kw. (2786)
94 (tinzaparin* or Innohep or UNII-7UQ7X4Y489).tw,kw. (1357)
95 (bemiparin* or hibor or phivor or ardeparin* or UNII-N3927D01PB).tw,kw. (343)
96 (certoparin* or Alphaparin* or Alpha-parin* or Mono-Embolex or Monoembolex).tw,kw. (370)
97 (Reviparin* or Clivarin* or LU 47311 or LU47311 or lomorin).tw,kw. (510)
98 (parnaparin* or parvoparin* or fluxum or lohepa or lowhepa or minidalton or op 2123 or CB-01-05-MMX).tw,kw. (258)
99 (semuloparin* or mulsevo or visamerin or AVE 5026 or AVE5026 or UNII-4QW4AN84NQ).tw,kw. (117)
100 sevuparin*.tw,kw. (8)
101 (ardeparin* or normifio or normiflo or rd heparin or wy 90493 or wy90493).tw,kw. (203)
102 (adomiparin* or "m 118" or m118).tw,kw. (164)
103 antixarin*.tw. (4)
104 ("cy 222" or cy222).tw,kw. (224)
105 (danaproid or "kb 101" or kb101 or lomoparan or lomoparin or mucoglucuronan or org 10172 or org10172 or orgaran).tw,kw. (1751)
106 308068-55-5.rn. (0)
107 deligoparin*.tw,kw. (3)
108 ((heparin adj1 dihydergot) or (dihydroergotamine adj1 heparin) or Embolex or (heparin adj1 DHE)).tw,kw. (433)
109 idrabiotaparinux.tw,kw. (80)
110 idraparinux.tw,kw. (316)
111 162610-17-5.rn. (615)
112 livaraparin calcium.tw,kw. (0)
113 minolteparin*.tw,kw. (0)
114 rd 11885.tw,kw. (13)
115 tafoxiparin*.tw,kw. (3)
116 (fondaparinux or arixtra or quixidar or xantidar or "Org 31540" or "SR 90107" or "SR 90107A" or "UNII-X0Q6N9USOZ" or "UNII-J177FOW5JL").tw,kw. (4212)
117 114870-03-0.rn. (5086)
118 or/87-117 (65921)
119 deep vein thrombosis/ (61884)
120 upper extremity deep vein thrombosis/ or lower extremity deep vein thrombosis/ (1235)
121 (deep adj (venous or vein$1 or vena) adj2 thrombos*).tw,kw. (50364)
122 (("deep venous" or "deep vein") adj2 (thrombus or thrombophlebitis or "thrombo-phlebitis")).tw,kw. (571)
123 (DVT or DVTs).tw,kw. (20654)
124 venous thromboembolism/ (29282)

125 ((venous or vein$1 or vena) adj2 (thromboemboli* or thrombo-emboli*)).tw,kw. (39299)
126 (VTE or VTEs).tw,kw. (18180)
127 perioperative period/ (33277)
128 (peri-operative or perioperative).tw,kw. (157153)
129 (bridge or bridging).tw,kw. (139904)
130 (bridg* adj3 (anti-coagulation or anticoagulation)).tw,kw. (371)
131 (interrupt* adj3 warfarin).tw,kw. (277)
132 exp venous thromboembolism/pc [Prevention] (16994)
133 ((prevent* or prophyla* or chemoprophyla* or chemo-prophyla*) adj3 (thromboemboli* or thrombo-embolic* or thrombos#s or VTE or VTEs)).tw,kw. (28336)
134 (thromboprophyla* or thrombo-prophyla*).tw,kw. (8927)
135 postoperative complication/pc [Prevention] (56397)
136 or/119-135 (483430)
137 118 and 136 (28472)
138 exp animal experimentation/ or exp models animal/ or exp animal experiment/ or nonhuman/ or exp vertebrate/ (39552135)
139 exp humans/ or exp human experimentation/ or exp human experiment/ (30856297)
140 138 not 139 (8697447)
141 137 not 140 (28128)
142 (editorial or letter).pt. (2747543)
143 141 not 142 (26280)
144 exp practice guideline/ (372173)
145 (guideline* or standards or consensus* or recommendat* or practice parameter* or position statement* or policy statement* or CPG or CPGs or best practice*).ti. (276656)
146 (care adj3 (path or paths or pathway or pathways or map or maps or plan or plans or standard or standards)).ti. (14909)
147 ((critical or clinical or practice) adj3 (path or paths or pathway or pathways or protocol)).ti. (6518)
148 (algorithm* adj3 (pharmacotherap* or pharmaco-therap* or chemotherap* or chemo-therap* or chemotreatment* or chemo-treatment* or therap* or treatment* or intervention*)).ti. (2478)
149 clinical algorithm*.ti. (385)
150 or/144-149 (585199)
151 143 and 150 (2414)
152 limit 151 to yr="2005-current" (1951)
153 limit 152 to english language (1765)
154 153 use emez (1625)
155 86 or 154 (1848)
156 remove duplicates from 155 (1667) [TOTAL UNIQUE RECORDS]
157 156 use prmz (213) [MEDLINE UNIQUE RECORDS]
158 156 use emez (1454) [EMBASE UNIQUE RECORDS]

LWMH Indications – Guidelines (update)

2017 May 12

OVID

Embase 1980 to 2017 Week 19, Ovid MEDLINE(R) Epub Ahead of Print, In-Process & Other Non-Indexed Citations, Ovid MEDLINE(R) Daily and Ovid MEDLINE(R) 1946 to Present

Search Strategy:
----
1 exp Heparin, Low-Molecular-Weight/ (63319)
2 LMWH.tw,kw. (11730)
3 ((low molecular weight or LMW) adj1 heparin).tw,kw. (23455)
4 Heparin, Low-Molecular-Weight.rn. (7852)
5 (Dalteparin* or FR-860 or Fragmin or Fragmine or Kabi-2165 or "K 2165'" or K2165 or Tedelparin or low liquemin).tw,kw. (4579)
6 dalteparin.rn. (848)
7 (Enoxaparin* or Clexan* or EMT-966 or EMT-967 or HSDB 7846 or Klexane or Lovenox or PK10169 or PK 10169 or "PK-10,169" or RP 54563 or UNII-8NZ41MIK1O).tw,kw. (12907)
8 enoxaparin.rn. (21035)
9 (nadroparin* or CY 216 or CY 216d or CY216 or CY216d or Fraxiparin* or LMF CY-216 or Nadroparin Calcium or Nadroparine or Nadrohep or Fraxodi or Seleparin* or Tedegliparin*).tw,kw. (2925)
10 nadroparin.rn. (4535)
11 (tinzaparin* or Innohep or UNII-7UQ7X4Y489).tw,kw. (1487)
12 tinzaparin.rn. (289)
13 (bemiparin* or hibor or phivor or ardeparin* or UNII-N3927D01PB).tw,kw. (379)
14 (certoparin* or Alphaparin* or Alpha-parin* or Mono-Embolex or Monoembolex).tw,kw. (389)
15 (Reviparin* or Clivarin* or LU 47311 or LU47311 or lomorin).tw,kw. (511)
16 reviparin.rn. (86)
17 (parnaparin* or parvoparin* or fluxum or lohepa or lowhepa or minidalton or op 2123 or CB-01-05-MMX).tw,kw. (265)
18 Parnaparin.rn. (36)
19 (semuloparin* or mulsevo or visamerin or AVE 5026 or AVE5026 or UNII-4QW4AN84NQ).tw,kw. (126)
20 semuloparin.rn. (11)
21 sevuparin*.tw,kw. (14)
22 sevuparin.rn. (0)
23 (ardeparin* or normifio or normiflo or rd heparin or wy 90493 or wy90493).tw,kw. (205)
24 ardeparin.rn. (22)
25 (adomiparin* or "m 118" or m118).tw,kw. (188)
26 adomiparin.rn. (0)
27 ("cy 222" or cy222).tw,kw. (227)
28 cy 222.rn. (168)
29 (danaproid or "kb 101" or kb101 or lomoparan or lomoparin or mucoglucuronan or org 10172 or org10172 or orgaran).tw,kw. (1903)
30 danaproid.rn. (434)
31 deligoparin*.tw,kw. (3)
32 deligoparin.rn. (1)
33 ((heparin adj1 dihydergot) or (dihydroergotamine adj1 heparin) or Embolex or (heparin adj1 DHE)).tw,kw. (427)
34 heparin-dihydergot.rn. (73)

35 idrabiotaparinux.tw,kw. (87)
36 idrabiotaparinux.rn. (105)
37 idraparinux.tw,kw. (334)
38 idraparinux.rn. (707)
39 livaraparin calcium.tw,kw. (0)
40 livaraparin calcium.rn. (0)
41 minolteparin*.tw,kw. (0)
42 minolteparin.rn. (0)
43 rd 11885.tw,kw. (13)
44 rd 11885.rn. (0)
45 tafoxiparin*.tw,kw. (4)
46 tafoxiparin.rn. (1)
47 (fondaparinux or arixtra or quixidar or xantidar or "Org 31540" or "SR 90107" or "SR 90107A" or "UNII-X0Q6N9USOZ" or "UNII-J177FOW5JL").tw,kw. (4691)
48 (fondaparinux or fondaparinux sodium).rn. (6568)
49 or/1-48 (73514)
50 Venous Thrombosis/ (29881)
51 Upper Extremity Deep Vein Thrombosis/ (1088)
52 (deep adj (venous or vein$1 or vena) adj2 thrombos*).tw,kw. (56319)
53 (("deep venous" or "deep vein") adj2 (thrombus or thrombophlebitis or "thrombo-phlebitis")).tw,kw. (627)
54 (DVT or DVTs).tw,kw. (23996)
55 Venous Thromboembolism/ (35066)
56 ((venous or vein$1 or vena) adj2 (thromboemboli* or thrombo-emboli*)).tw,kw. (46445)
57 (VTE or VTEs).tw,kw. (22671)
58 exp Perioperative Care/(174459)
59 exp Perioperative Period/ (107437)
60 (peri-operative or perioperative).tw,kw. (184310)
61 (bridge or bridging).tw,kw. (157953)
62 (bridg* adj3 (anti-coagulation or anticoagulation)).tw,kw. (512)
63 (interrupt* adj3 warfarin).tw,kw. (336)
64 exp Venous Thromboembolism/pc(18496)
65 ((prevent* or prophyla* or chemoprophyla* or chemo-prophyla*) adj3 (thromboemboli* or thrombo-embolic* or thrombos#s or VTE or VTEs)).tw,kw. (31426)
66 (thromboprophyla* or thrombo-prophyla*).tw,kw. (10344)
67 Postoperative Complications/pc(46416)
68 or/ 50-67(703599)
69 49 and 68(30537)
70 exp Animals/ not (exp Animals/ and Humans/)(14420969)
71 69 not 70(21009)
72 (comment or editorial or interview or letter or news).pt. (3261749)
73 71 not 72(19457)
74 (guideline or practice guideline or consensus development conference or "consensus development conference, NIH").pt. (38394)
75 exp Clinical Protocol/ (232887)
76 Critical Pathways/ (13060)
77 (guideline* or standards or consensus* or recommendat* or practice parameter* or position statement* or policy statement* or CPG or CPGs or best practice*).ti. (309759)
78 (care adj3 (path or paths or pathway or pathways or map or maps or plan or plans or standard or standards)).ti. (17017)
79 ((critical or clinical or practice) adj3 (path or paths or pathway or pathways or protocol)).ti. (7397)
80 (algorithm* adj3 (pharmacotherap* or pharmaco-therap* or chemotherap* or chemo-therap* or chemotreatment* or

chemo-treatment* or therap* or treatment* or intervention*)).ti. (2959)
81 clinical algorithm*.ti. (417)
82 or/ 74-81(582520)
83 73 and 82(938)
84 limit 83 to yr="2015-current"(100)
85 limit 84 to english language(87)
86 85 use ppez(54)
87 exp low molecular weight heparin/ (63319)
88 LMWH.tw,kw. (11730)
89 ((low molecular weight or LMW) adj1 heparin).tw,kw. (23455)
90 (Dalteparin* or FR-860 or Fragmin or Fragmine or Kabi-2165 or "K 2165'" or K2165 or Tedelparin or low liquemin).tw,kw. (4579)
91 (Enoxaparin* or Clexan* or EMT-966 or EMT-967 or HSDB 7846 or Klexane or Lovenox or PK10169 or PK 10169 or "PK-10,169" or RP 54563 or UNII-8NZ41MIK1O).tw,kw. (12907)
92 679809-58-6.rn. (9548)
93 (nadroparin* or CY 216 or CY 216d or CY216 or CY216d or Fraxiparin* or LMF CY-216 or Nadroparin Calcium or Nadroparine or Nadrohep or Fraxodi or Seleparin* or Tedegliparin*).tw,kw. (2925)
94 (tinzaparin* or Innohep or UNII-7UQ7X4Y489).tw,kw. (1487)
95 (bemiparin* or hibor or phivor or ardeparin* or UNII-N3927D01PB).tw,kw. (379)
96 (certoparin* or Alphaparin* or Alpha-parin* or Mono-Embolex or Monoembolex).tw,kw. (389)
97 (Reviparin* or Clivarin* or LU 47311 or LU47311 or lomorin).tw,kw. (511)
98 (parnaparin* or parvoparin* or fluxum or lohepa or lowhepa or minidalton or op 2123 or CB-01-05-MMX).tw,kw. (265)
99 (semuloparin* or mulsevo or visamerin or AVE 5026 or AVE5026 or UNII-4QW4AN84NQ).tw,kw. (126)
100 sevuparin*.tw,kw. (14)
101 (ardeparin* or normifio or normiflo or rd heparin or wy 90493 or wy90493).tw,kw. (205)
102 (adomiparin* or "m 118" or m118).tw,kw. (188)
103 antixarin*.tw. (4)
104 ("cy 222" or cy222).tw,kw. (227)
105 (danaproid or "kb 101" or kb101 or lomoparan or lomoparin or mucoglucuronan or org 10172 or org10172 or orgaran).tw,kw. (1903)
106 308068-55-5.rn. (0)
107 deligoparin*.tw,kw. (3)
108 ((heparin adj1 dihydergot) or (dihydroergotamine adj1 heparin) or Embolex or (heparin adj1 DHE)).tw,kw. (427)
109 idrabiotaparinux.tw,kw. (87)
110 idraparinux.tw,kw. (334)
111 162610-17-5.rn. (628)
112 livaraparin calcium.tw,kw. (0)
113 minolteparin*.tw,kw. (0)
114 rd 11885.tw,kw. (13)
115 tafoxiparin*.tw,kw. (4)
116 (fondaparinux or arixtra or quixidar or xantidar or "Org 31540" or "SR 90107" or "SR 90107A" or "UNII-X0Q6N9USOZ" or "UNII-J177FOW5JL").tw,kw. (4691)
117 114870-03-0.rn. (5566)
118 or/ 87-117(73238)
119 deep vein thrombosis/ (70379)
120 upper extremity deep vein thrombosis/ or lower extremity deep vein thrombosis/ (1796)
121 (deep adj (venous or vein$1 or vena) adj2 thrombos*).tw,kw. (56319)
122 (("deep venous" or "deep vein") adj2 (thrombus or thrombophlebitis or "thrombo-phlebitis")).tw,kw. (627)
123 (DVT or DVTs).tw,kw. (23996)
124 venous thromboembolism/ (35066)

125 ((venous or vein$1 or vena) adj2 (thromboemboli* or thrombo-emboli*)).tw,kw. (46445)

126 (VTE or VTEs).tw,kw. (22671)

127 perioperative period/ (38453)

128 (peri-operative or perioperative).tw,kw. (184310)

129 (bridge or bridging).tw,kw. (157953)

130 (bridg* adj3 (anti-coagulation or anticoagulation)).tw,kw. (512)

131 (interrupt* adj3 warfarin).tw,kw. (336)

132 exp venous thromboembolism/pc [Prevention] (18496)

133 ((prevent* or prophyla* or chemoprophyla* or chemo-prophyla*) adj3 (thromboemboli* or thrombo-embolic* or thrombos#s or VTE or VTEs)).tw,kw. (31426)

134 (thromboprophyla* or thrombo-prophyla*).tw,kw. (10344)

135 postoperative complication/pc [Prevention] (58994)

136 or/119-135(549491)

137 118 and 136(31825)

138 exp animal experimentation/ or exp models animal/ or exp animal experiment/ or nonhuman/ or exp vertebrate/ (44062939)

139 exp humans/ or exp human experimentation/ or exp human experiment/ (34950094)

140 138 not 139(9114466)

141 137 not 140(31464)

142 (editorial or letter).pt. (2891858)

143 141 not 142(29465)

144 exp practice guideline/ (423414)

145 (guideline* or standards or consensus* or recommendat* or practice parameter* or position statement* or policy statement* or CPG or CPGs or best practice*).ti. (309759)

146 (care adj3 (path or paths or pathway or pathways or map or maps or plan or plans or standard or standards)).ti. (17017)

147 ((critical or clinical or practice) adj3 (path or paths or pathway or pathways or protocol)).ti. (7397)

148 (algorithm* adj3 (pharmacotherap* or pharmaco-therap* or chemotherap* or chemo-therap* or chemotreatment* or chemo-treatment* or therap* or treatment* or intervention*)).ti. (2959)

149 clinical algorithm*.ti. (417)

150 or/144-149 (662776)

151 143 and 150 (2692)

152 limit 151 to yr="2015-current"(403)

153 limit 152 to english language(380)

154 153 use emez(346)

155 86 or 154(400)

156 remove duplicates from 155 (362)

157 156 use ppez (24)

158 156 use emez (338)

156 remove duplicates from 155 (362)

157 156 use ppez (24)

158 156 use emez (338)

LWMH – Indications – Reviews

2017 May 13

OVID

Embase 1980 to 2017 Week 20, Ovid MEDLINE(R) Epub Ahead of Print, In-Process & Other Non-Indexed Citations, Ovid MEDLINE(R) Daily and Ovid MEDLINE(R) 1946 to Present

Search Strategy:
----

1 exp Heparin, Low-Molecular-Weight/ (63535)
2 LMWH.tw,kw. (11748)
3 ((low molecular weight or LMW) adj1 heparin).tw,kw. (23496)
4 Heparin, Low-Molecular-Weight.rn. (7852)
5 (Dalteparin* or FR-860 or Fragmin or Fragmine or Kabi-2165 or "K 2165'" or K2165 or Tedelparin or low liquemin).tw,kw. (4583)
6 dalteparin.rn. (848)
7 (Enoxaparin* or Clexan* or EMT-966 or EMT-967 or HSDB 7846 or Klexane or Lovenox or PK10169 or PK 10169 or "PK-10,169" or RP 54563 or UNII-8NZ41MIK1O).tw,kw. (12935)
8 enoxaparin.rn. (21126)
9 (nadroparin* or CY 216 or CY 216d or CY216 or CY216d or Fraxiparin* or LMF CY-216 or Nadroparin Calcium or Nadroparine or Nadrohep or Fraxodi or Seleparin* or Tedegliparin*).tw,kw. (2926)
10 nadroparin.rn. (4546)
11 (tinzaparin* or Innohep or UNII-7UQ7X4Y489).tw,kw. (1490)
12 tinzaparin.rn. (289)
13 (bemiparin* or hibor or phivor or ardeparin* or UNII-N3927D01PB).tw,kw. (380)
14(certoparin* or Alphaparin* or Alpha-parin* or Mono-Embolex or Monoembolex).tw,kw. (389)
15 (Reviparin* or Clivarin* or LU 47311 or LU47311 or lomorin).tw,kw. (512)
16 reviparin.rn. (86)
17 (parnaparin* or parvoparin* or fluxum or lohepa or lowhepa or minidalton or op 2123 or CB-01-05-MMX).tw,kw. (266)
18 Parnaparin.rn. (36)
19 (semuloparin* or mulsevo or visamerin or AVE 5026 or AVE5026 or UNII-4QW4AN84NQ).tw,kw. (126)
20 semuloparin.rn. (11)
21 sevuparin*.tw,kw. (14)
22 sevuparin.rn. (0)
23 (ardeparin* or normifio or normiflo or rd heparin or wy 90493 or wy90493).tw,kw. (206)
24 ardeparin.rn. (22)
25 (adomiparin* or "m 118" or m118).tw,kw. (188)
26 adomiparin.rn. (0)
27 ("cy 222" or cy222).tw,kw. (227)
28 cy 222.rn. (168)
29 (danaproid or "kb 101" or kb101 or lomoparan or lomoparin or mucoglucuronan or org 10172 or org10172 or orgaran).tw,kw. (1909)
30 danaproid.rn. (434)
31 deligoparin*.tw,kw. (3)
32 deligoparin.rn(1)
33 ((heparin adj1 dihydergot) or (dihydroergotamine adj1 heparin) or Embolex or (heparin adj1 DHE)).tw,kw. (428)
34 heparin-dihydergot.rn. (73)

35 idrabiotaparinux.tw,kw. (87)
36 idrabiotaparinux.rn. (105)
37 idraparinux.tw,kw. (334)
38 idraparinux.rn. (708)
39 livaraparin calcium.tw,kw. (0)
40 livaraparin calcium.rn. (0)
41 minolteparin*.tw,kw. (0)
42 minolteparin.rn. (0)
43 rd 11885.tw,kw. (13)
44 rd 11885.rn. (0)
45 tafoxiparin*.tw,kw. (4)
46 tafoxiparin.rn. (1)
47 (fondaparinux or arixtra or quixidar or xantidar or "Org 31540" or "SR 90107" or "SR 90107A" or "UNII-X0Q6N9USOZ" or "UNII-J177FOW5JL").tw,kw. (4702)
48 (fondaparinux or fondaparinux sodium).rn. (6596)
49 or/1-48 (73737)
50 Venous Thrombosis/ (29890)
51 Upper Extremity Deep Vein Thrombosis(1095)
52 (deep adj (venous or vein$1 or vena) adj2 thrombos*).tw,kw. / (56445)
53 (("deep venous" or "deep vein") adj2 (thrombus or thrombophlebitis or "thrombo-phlebitis")).tw,kw. (628)
54 (DVT or DVTs).tw,kw. (24063)
55 Venous Thromboembolism (35232)
56 ((venous or vein$1 or vena) adj2 (thromboemboli* or thrombo-emboli*)).tw,kw. / (46605)
57 (VTE or VTEs).tw,kw. (22750)
58 exp Perioperative Care (174719)
59 exp Perioperative Period/ (107697)
60 (peri-operative or perioperative).tw,kw. / (184868)
61 (bridge or bridging).tw,kw. (158440)
62 (bridg* adj3 (anti-coagulation or anticoagulation)).tw,kw. (514)
63 (interrupt* adj3 warfarin).tw,kw. (338)
64 exp Venous Thromboembolism/pc(18567)
65 ((prevent* or prophyla* or chemoprophyla* or chemo-prophyla*) adj3 (thromboemboli* or thrombo-embolic* or thrombos#s or VTE or VTEs)).tw,kw. (31503)
66 (thromboprophyla* or thrombo-prophyla*).tw,kw. (10372)
67 Postoperative Complications/pc(46447)
68 or/50-67(705063)
69 49 and 68(30613)
70 exp Animals/ not (exp Animals/ and Humans/)(14439620)
71 69 not 70(21084)
72 (comment or editorial or interview or letter or news).pt. (3265957)
73 71 not 72(19528)
74 (guideline or practice guideline or consensus development conference or "consensus development conference, NIH").pt. (38406)
75 exp Clinical Protocol/(233323)
76 Critical Pathways/(13084)
77 (guideline* or standards or consensus* or recommendat* or practice parameter* or position statement* or policy statement* or CPG or CPGs or best practice*).ti. (310589)
78 (care adj3 (path or paths or pathway or pathways or map or maps or plan or plans or standard or standards)).ti. (17066)

79 ((critical or clinical or practice) adj3 (path or paths or pathway or pathways or protocol)).ti. (7409)
80 (algorithm* adj3 (pharmacotherap* or pharmaco-therap* or chemotherap* or chemo-therap* or chemotreatment* or chemo-treatment* or therap* or treatment* or intervention*)).ti. (2963)
81 clinical algorithm*.ti. (419)
82 or/74-81(583850)
83 73 and 82(942)
84 limit 83 to yr="2015-current"(104)
85 limit 84 to english language(91)
86 limit 73 to systematic reviews [Limit not valid in Embase; records were retained] (12479)
87 meta analysis.pt. (80082)
88 exp meta-analysis as topic/ (49506)
89 (meta-analy* or metanaly* or metaanaly* or met analy* or integrative research or integrative review* or integrative overview* or research integration or research overview* or collaborative review*).tw. (262555)
90 (systematic review* or systematic overview* or evidence-based review* or evidence-based overview* or (evidence adj3 (review* or overview*)) or meta-review* or meta-overview* or meta-synthes* or rapid review* or "review of reviews" or technology assessment* or HTA or HTAs).tw. (307305)
91 exp Technology assessment, biomedical/ (22060)
92 (cochrane or health technology assessment or evidence report).jw. (32589)
93 or/87-92(553494)
94 73 and 93(1256)
95 86 or 94(12629)
96 limit 95 to yr="2015-current"(1137)
97 limit 96 to english language(1094)
98 97 not 85(1041)
99 98 use ppez(127)
100 exp low molecular weight heparin/ (63535)
101 LMWH.tw,kw. (11748)
102 ((low molecular weight or LMW) adj1 heparin).tw,kw. (23496)
103 (Dalteparin* or FR-860 or Fragmin or Fragmine or Kabi-2165 or "K 2165'" or K2165 or Tedelparin or low liquemin).tw,kw. (4583)
104 (Enoxaparin* or Clexan* or EMT-966 or EMT-967 or HSDB 7846 or Klexane or Lovenox or PK10169 or PK 10169 or "PK-10,169" or RP 54563 or UNII-8NZ41MIK1O).tw,kw. (12935)
105 679809-58-6.rn. (9638)
106 (nadroparin* or CY 216 or CY 216d or CY216 or CY216d or Fraxiparin* or LMF CY-216 or Nadroparin Calcium or Nadroparine or Nadrohep or Fraxodi or Seleparin* or Tedegliparin*).tw,kw. (2926)
107 (tinzaparin* or Innohep or UNII-7UQ7X4Y489).tw,kw. (1490)
108 (bemiparin* or hibor or phivor or ardeparin* or UNII-N3927D01PB).tw,kw. (380)
109 (certoparin* or Alphaparin* or Alpha-parin* or Mono-Embolex or Monoembolex).tw,kw. (389)
110 (Reviparin* or Clivarin* or LU 47311 or LU47311 or lomorin).tw,kw. (512)
111 (parnaparin* or parvoparin* or fluxum or lohepa or lowhepa or minidalton or op 2123 or CB-01-05-MMX).tw,kw. (266)
112 (semuloparin* or mulsevo or visamerin or AVE 5026 or AVE5026 or UNII-4QW4AN84NQ).tw,kw. (126)
113 sevuparin*.tw,kw. (14)
114 (ardeparin* or normifio or normiflo or rd heparin or wy 90493 or wy90493).tw,kw. (206)
115 (adomiparin* or "m 118" or m118).tw,kw. (188)
116 antixarin*.tw. (4)
117 ("cy 222" or cy222).tw,kw. (227)
118 (danaproid or "kb 101" or kb101 or lomoparan or lomoparin or mucoglucuronan or org 10172 or org10172 or orgaran).tw,kw. (1909)

119 308068-55-5.rn. (0)
120 deligoparin*.tw,kw. (3)
121 ((heparin adj1 dihydergot) or (dihydroergotamine adj1 heparin) or Embolex or (heparin adj1 DHE)).tw,kw. (428)
122 idrabiotaparinux.tw,kw. (87)
123 idraparinux.tw,kw. (334)
124 162610-17-5.rn. (629)
125 livaraparin calcium.tw,kw. (0)
126 minolteparin*.tw,kw. (0)
127 rd 11885.tw,kw. (13)
128 tafoxiparin*.tw,kw. (4)
129 (fondaparinux or arixtra or quixidar or xantidar or "Org 31540" or "SR 90107" or "SR 90107A" or "UNII-X0Q6N9USOZ" or "UNII-J177FOW5JL").tw,kw. (4702)
130 114870-03-0.rn. (5594)
131 or/100-130/ (73461)
132 deep vein thrombosis/ (70617)
133 upper extremity deep vein thrombosis/ or lower extremity deep vein thrombosis(1814)
134 (deep adj (venous or vein$1 or vena) adj2 thrombos*).tw,kw. (56445)
135 (("deep venous" or "deep vein") adj2 (thrombus or thrombophlebitis or "thrombo-phlebitis")).tw,kw. (628)
136 (DVT or DVTs).tw,kw. (24063)
137 venous thromboembolism/ (35232)
138 ((venous or vein$1 or vena) adj2 (thromboemboli* or thrombo-emboli*)).tw,kw. (46605)
139 (VTE or VTEs).tw,kw. (22750)
140 perioperative period/ (38705)
141 (peri-operative or perioperative).tw,kw. (184868)
142 (bridge or bridging).tw,kw. (158440)
143 (bridg* adj3 (anti-coagulation or anticoagulation)).tw,kw. (514)
144 (interrupt* adj3 warfarin).tw,kw. (338)
145 exp venous thromboembolism/pc [Prevention] (18567)
146 ((prevent* or prophyla* or chemoprophyla* or chemo-prophyla*) adj3 (thromboemboli* or thrombo-embolic* or thrombos#s or VTE or VTEs)).tw,kw. (31503)
147 (thromboprophyla* or thrombo-prophyla*).tw,kw. (10372)
148 postoperative complication/pc [Prevention] (59023)
149 or/132-148(551039)
150 131 and 149(31908)
151 exp animal experimentation/ or exp models animal/ or exp animal experiment/ or nonhuman/ or exp vertebrate/ (44162680)
152 exp humans/ or exp human experimentation/ or exp human experiment/ (35024400)
153 151 not 152 (9139902)
154 150 not 153 (31546)
155 (editorial or letter).pt. (2896032)
156 154 not 155 (29543)
157 exp practice guideline/ (425582)
158 (guideline* or standards or consensus* or recommendat* or practice parameter* or position statement* or policy statement* or CPG or CPGs or best practice*).ti. (310589)
159 (care adj3 (path or paths or pathway or pathways or map or maps or plan or plans or standard or standards)).ti. (17066)
160 ((critical or clinical or practice) adj3 (path or paths or pathway or pathways or protocol)).ti. (7409)
161 (algorithm* adj3 (pharmacotherap* or pharmaco-therap* or chemotherap* or chemo-therap* or chemotreatment* or chemo-treatment* or therap* or treatment* or intervention*)).ti (2963).

162 clinical algorithm*.ti. (419)
163 or/157-162 (665409)
164 156 and 163(2707)
165 limit 164 to yr="2015-current"(418)
166 limit 165 to english language(395)
167 meta-analysis/ (205005)
168 "systematic review"/ (135081)
169 "meta analysis (topic)"/ (33423)
170 (meta-analy* or metanaly* or metaanaly* or met analy* or integrative research or integrative review* or integrative overview* or research integration or research overview* or collaborative review*).tw. (262555)
171 (systematic review* or systematic overview* or evidence-based review* or evidence-based overview* or (evidence adj3 (review* or overview*)) or meta-review* or meta-overview* or meta-synthes* or "review of reviews" or technology assessment* or HTA or HTAs).tw. (306845)
172 biomedical technology assessment/(20939)
173 (cochrane or health technology assessment or evidence report).jw. (32589)
174 or/167-173(596923)
175 156 and 174(2549)
176 limit 175 to yr="2015-current"(466)
177 limit 176 to english language(460)
178 177 not 166(400)
179 178 use emez(294)
180 99 or 179(421)
181 remove duplicates from 180(333)
182 181 use ppez(49)
183 181 use emez(284)

Cochrane Library

Search Name:

Date Run: 13/05/17 17:05:28.764

Search Strategy:

----

1 [mh "Heparin, Low-Molecular-Weight"] 1896
2 LMWH:ti,ab,kw 929
3 (("low molecular weight" or LMW) near/1 heparin):ti,ab,kw 3013
4 (Dalteparin* or "FR-860" or FR860 or Fragmin or Fragmine or "Kabi-2165" or "K-2165" or K2165 or Tedelparin or "low liquemin"):ti,ab,kw 652
5 (Enoxaparin* or Clexan* or "EMT-966" or EMT966 or "EMT-967" or EMT967 or "HSDB 7846" or Klexane or Lovenox or "PK 10169" or "PK-10,169" or PK10169 or "RP 54563" or "UNII-8NZ41MIK1O"):ti,ab,kw 1648
6 (nadroparin* or "CY 216" or "CY 216d" or CY216 or CY216d or Fraxiparin* or "LMF CY-216" or Nadroparin Calcium or Nadroparine or Nadrohep or Fraxodi or Seleparin* or Tedegliparin*):ti,ab,kw 359
7 (tinzaparin* or Innohep or logiparin* or lhn1 or "UNII-7UQ7X4Y489"):ti,ab,kw 203
8 (bemiparin* or hibor or phivor or ardeparin* or "UNII-N3927D01PB"):ti,ab,kw 49
9 (certoparin* or Alphaparin* or Alpha-parin* or Mono-Embolex or Monoembolex):ti,ab,kw 77
10 (Reviparin* or Clivarin* or LU47311 or "LU 47311" or lomorin):ti,ab,kw 68
11 (parnaparin* or parvoparin* or fluxum or lohepa or lowhepa or minidalton or "op 2123" or "CB-01-05 MMX"):ti,ab,kw 36
12 (semuloparin* or mulsevo or visamerin or "AVE 5026" or AVE5026 or "UNII-4QW4AN84NQ"):ti,ab,kw 24
13 sevuparin*:ti,ab,kw 4
14 (ardeparin* or normifio or normiflo or "rd heparin" or "wy 90493" or wy90493):ti,ab,kw 17
15 (adomiparin* or "m 118" or m118):ti,ab,kw 6
16 antixarin*:ti,ab,kw 2
17 ("cy 222" or cy222):ti,ab,kw 16
18 (danaproid or "kb 101" or kb101 or lomoparan or lomoparin or mucoglucuronan or "org 10172" or org10172 or orgaran):ti,ab,kw 101
19 deligoparin*:ti,ab,kw 0
20 ((heparin near/1 dihydergot) or (dihydroergotamine near/1 heparin) or Embolex or (heparin near/1 DHE)):ti,ab,kw 114
21 idrabiotaparinux:ti,ab,kw 15
22 idraparinux:ti,ab,kw 37
23 "livaraparin calcium":ti,ab,kw 0
24 minolteparin*:ti,ab,kw 0
25 "rd 11885":ti,ab,kw 0
26 tafoxiparin*:ti,ab,kw 0
27 (fondaparinux or arixtra or quixidar or xantidar or "Org 31540" or "SR 90107" or "SR 90107A" or "UNII X0Q6N9USOZ" or "UNII-J177FOW5JL"):ti,ab,kw 343
28 {or #1-#27} 4992
29 [mh ^"Venous Thrombosis"] 1186
30 [mh "Upper Extremity Deep Vein Thrombosis"] 13
31 (deep next (venous or vein or veins or vena) near/2 thrombos*):ti,ab,kw 4207
32 (("deep venous" or "deep vein") near/2 (thrombus or thrombophlebitis or "thrombo-phlebitis")):ti,ab,kw 16
33 (DVT or DVTs):ti,ab,kw 1348
34 [mh "Venous Thromboembolism"] 560

35 ((venous or vein or veins or vena) near/2 (thromboemboli* or (thrombo next emboli*))):ti,ab,kw 2700
36 (VTE or VTEs):ti,ab,kw 917
37 [mh "Perioperative Care"] 11730
38 [mh "Perioperative Period"] 7342
39 ("peri-operative" or perioperative):ti,ab,kw 10853
40 (bridge or bridging):ti,ab,kw 1455
41 (bridg* near/3 (anti-coagulation or anticoagulation)):ti,ab,kw 35
42 (interrupt* adj3 warfarin):ti,ab,kw 0
43 [mh "Venous Thromboembolism"/PC] 276
44 ((prevent* or prophyla* or chemoprophyla* or (chemo next prophyla*)) near/3 (thromboemboli* or (thrombo next embolic*) or thrombosis or thromboses or VTE or VTEs)):ti,ab,kw 3509
45 (thromboprophyla* or (thrombo next prophyla*)):ti,ab,kw 779
46 [mh ^"Postoperative Complications"/PC] 5804
47 {or #29-#46} 39291
48 #28 and #47 Publication Year from 2015 to 2017 4
