# Supplementary material for: A systematic review of clinical practice guidelines on the use of low molecular weight heparin and fondaparinux for the treatment and prevention of venous thromboembolism: Implications for research and policy decision-making
Source: PLoS One. 2018 Nov 9;13(11):e0207410. doi: 10.1371/journal.pone.0207410 (PMC6226206; doi:10.1371/journal.pone.0207410)
Supplement: S3 Appendix — (PDF) [file pone.0207410.s003.pdf]

Table of Contents:

Table 1: Recommendations for the treatment of VTE in pregnant and/or lactating women 2
Table 2: Recommendations for the treatment of VTE in patients who fail treatment with warfarin 5
Table 3: Recommendations for the treatment of VTE in patients in whom treatment with warfarin is either not tolerated or contraindicated 7
Table 4: Treatment of symptomatic, acute, VTE in patients with cancer 8
Table 5: Recommendations for the treatment of VTE in patients without cancer 14
Table 6: Recommendations for peri-operative bridging for patients who require long-term warfarin and must discontinue due to surgery 16
Table 7: Recommendations for the prevention of VTE in patients with cancer 18
Table 8: Recommendations for the prevention of VTE in patients undergoing non-orthopedic surgery 21
Table 9: Recommendations for the post-operative prophylaxis of VTE in patients undergoing surgery of the lower limbs 31
References 34

Table 1: Recommendations for the treatment of VTE in pregnant and/or lactating women

Guideline Identification	Publication Year	CPG Quality	Recommendation	Standardized Level of Evidence	Thematic Code	Comparative Preference	Medications Discussed
Scottish Intercollegiate Guidelines Network (SIGN) (1)	**2013**	**High**	LMWH should be used with caution for those in whom standard or weight- adjusted dosing is likely to be unreliable, especially in…pregnant women	**C**	Pregnancy	None	LMWH
Chan et al. (2)	**2014**	**Low**	Low molecular weight heparin is the preferred pharmacologic agent over unfractionated heparin for the treatment of venous thromboembolism in pregnancy. (II-2A)	**B**	Pregnancy	LMWH over UFH	LMWH
			Therapeutic low molecular weight heparin may be started or restarted at least 24 hours after a single injection neuraxial block and a minimum of 4 hours after neuraxial catheter removal, providing there is full neurological recovery and no evidence of active bleeding or coagulopathy. (III-B)	**D**	Pregnancy; Neuraxial block; Treatment_duration	None	LMWH
Venous Thromboembolism Guideline Team (University of Michigan) (3)	**2014**	**Low**	Patients who can receive heparin but cannot take warfarin (e.g., during pregnancy) may be anticoagulated with full-dose subcutaneous heparin [IA], preferably LMWH.	**B**	Pregnancy; Contraindicated	LMWH is the preferred heparin	LMWH
Cardiovascular Disease Educational and Research Trust et al. (4)	**2013**	**Moderate**	Breast feeding is not contraindicated with either LMWH, LDUH or warfarin (level of evidence: low)	**B**	Breastfeeding	None	LMWH
			During pregnancy LMWH is the treatment of choice throughout pregnancy and for the first six weeks after delivery (level of evidence: low)	**B**	Pregnancy; Delivery; Treatment_duration	None	LMWH

Bates et al. (5)	**2012**	**High**	For pregnant patients, we recommend LMWH for the prevention and treatment of VTE, instead of UFH (Grade 1B)	**B**	Pregnancy	LMWH over UFH	LMWH
			For pregnant women with acute VTE, we recommend therapy with adjusted-dose subcutaneous LMWH over adjusted-dose UFH (Grade 1B).	**B**	Pregnancy	LMWH over UFH	LMWH
			For pregnant women with acute VTE, we recommend LMWH over vitamin K antagonist treatment antenatally (Grade 1A).	**A**	Pregnancy	LMWH over VKA	LMWH
			For pregnant women, we suggest limiting the use of fondaparinux and parenteral direct thrombin inhibitors to those with severe allergic reactions to heparin (eg, HIT) who cannot receive danaparoid (Grade 2C).	**C**	Pregnancy; contraindicated	None	FDP
			For women receiving anticoagulation for the treatment of VTE who become pregnant, we recommend LMWH over vitamin K antagonists during the first trimester (Grade 1A)	**A**	Pregnancy	LMWH over VKA	LMWH
			For women receiving anticoagulation for the treatment of VTE who become pregnant, we recommend LMWH over vitamin K antagonists…in the second and third trimesters (Grade 1B)	**B**	Pregnancy	LMWH over VKA	LMWH
			For women receiving anticoagulation for the treatment of VTE who become pregnant, we recommend LMWH over vitamin K antagonists… when delivery is imminent (Grade 1A).	**A**	Pregnancy	LMWH over VKA	LMWH

			For lactating women using LMWH, danaparoid, or r-hirudin who wish to breast-feed, we recommend continuing the use of LMWH, danaparoid, or r-hirudin (Grade 1B).	**B**	Breastfeeding	None	LMWH
			For breast-feeding women, we suggest alternative anticoagulants rather than fondaparinux (Grade 2C).	**C**	Breastfeeding	Alternative anticoagulants over FDP	FDP
			For pregnant women receiving adjusted dose LMWH therapy and where delivery is planned, we recommend discontinuation of LMWH at least 24 h prior to induction of labor or cesarean section (or expected time of neuraxial anesthesia) rather than continuing LMWH up until the time of delivery (Grade 1B).	**B**	Pregnancy; Delivery; Neuraxial block	None	LMWH
James et al. (6)	**2014**	**Low**	Because warfarin, LMWH, and unfractionated heparin do not accumulate in breast milk and do not induce an anticoagulant effect in the infant, these anticoagulants are compatible with breastfeeding (Level B).	**C**	Breastfeeding	None	LMWH
			It is recommended to withhold neuraxial blockade for 10–12 hours after the last prophylactic dose of LMWH or 24 hours after the last therapeutic dose of LMWH (Level C).	**D**	Pregnancy; Delivery	None	LMWH
			Women receiving either therapeutic or prophylactic anticoagulation may be converted from LMWH to the shorter half-life unfractionated heparin in the last month of pregnancy or sooner if delivery appears imminent (Level C).	**D**	Pregnancy; Delivery	None	LMWH

Table 2: Recommendations for the treatment of VTE in patients who fail treatment with warfarin

Guideline Identification	Publication Year	CPG Quality	Recommendation	Standardized Level of Evidence	Thematic Code	Comparative Preference	Medications Discussed
Farge et al. (7)	**2016**	**High**	In the event of VTE recurrence, three options can be considered: (1) increase in LMWH dose (by 20–25%) in patients treated with LMWH; (2) switch from VKA to LMWH in patients treated with VKA; and (3) inferior vena cava filter insertion—with continued anticoagulant therapy, unless contraindicated (guidance, based on evidence of very low quality and an unknown balance between desirable and undesirable effects).	**C**	Switch; Failed_treatment; Recurrence	None	LMWH
Carrier et al. (8)	**2015**	**Moderate**	In patients with active cancer and a history of vte who develop an objectively confirmed vte recurrence during active anticoagulation with either lmwh or warfarin, we recommend against switching to doacs or fondaparinux. [Level of evidence: iv; Level of agreement: 76% (n = 16) strongly agreed, 10% (n = 2) somewhat agreed, 10% (n = 2) neutral, 5% (n = 1) somewhat disagreed]	**C**	Cancer; Failed_treatment; Recurrence	LMWH or warfarin over FDP	FDP, LMWH
			In patients with active cancer and a history of vte who develop an objectively confirmed vte recurrence during active anticoagulation with warfarin, we recommend switching to a lmwh at full therapeutic dose for a minimum of 4 weeks; expert consensus would recommend long-term therapy. [Level of evidence: iia; Level of agreement: 95% (n = 20) strongly agreed, 5% (n = 1) somewhat agreed]	**B**	Cancer; Failed_treatment; Switch; Recurrence; Treatment_duration	None	LMWH
Kearon et al. (9)	**2016**	**High**	In patients who have recurrent VTE on VKA therapy (in the therapeutic range) or on dabigatran, rivaroxaban, apixaban, or edoxaban (and are believed to be compliant), we suggest switching to treatment with LMWH at least temporarily (Grade 2C).	**C**	Switch; Failed_treatment; Recurrence; Treatment_duration	None	LMWH

Streiff et al. (10)	**2011**	**Moderate**	Patients with recurrent VTE and a therapeutic INR while on warfarin therapy can be switched to heparin (LMWH preferred) or fondaparinux.	**C**	Switch; Failed_treatment; Recurrence	LMWH over FDP	LMWH, FDP
			For patients with recurrent VTE and a subtherapeutic INR while on warfarin therapy, increase warfarin dose and treat with parenteral agent until INR target achieved or consider switching to heparin (LMWH preferred) or fondaparinux	**C**	Switch; Failed_treatment; Recurrence	LMWH over FDP	LMWH, FDP

Table 3: Recommendations for the treatment of VTE in patients in whom treatment with warfarin is either not tolerated or contraindicated

Guideline Identification	Publication Year	CPG Quality	Recommendation	Standardized Level of Evidence	Thematic Code	Comparative Preference	Medications Discussed
Venous Thromboembolism Guideline Team (University of Michigan) (3)	**2014**	**Low**	If warfarin contraindicated. Patients who can receive heparin but cannot take warfarin (e.g., during pregnancy) may be anticoagulated with full-dose subcutaneous heparin [IA], preferably LMWH.	B	Pregnancy; Contraindicated	LMWH is the preferred heparin	LMWH

Table 4: Treatment of symptomatic, acute, VTE in patients with cancer

Guideline Identification	Publication Year	CPG Quality	Recommendation	Standardized Level of Evidence	Thematic Code	Comparative Preference	Medications Discussed
Cardiovascular Disease Educational and Research Trust et al. (4)	**2013**	Moderate	In patients with a history of cancer LMWH for 3-6 months is the initial treatment (level of evidence: high)	**A**	Cancer; Treatment_duration	None	LMWH
			The initial and long term treatment of DVT and PE in patients with cancer is LMWH administered for 3-6 months (level of evidence: high). If the health care economics of a system do not allow for use of long term LMWH, it is acceptable to treat initially with UFH or LMWH followed by long-term VKA therapy (level of evidence: high).	**A**	Cancer; Resources; Treatment_duration	None	LMWH
Farge et al. (7)	**2016**	High	Low-molecular-weight heparin (LMWH) is recommended for the initial treatment of established VTE in patients with cancer (grade 1B).	**B**	Cancer	None	LMWH
			Fondaparinux and unfractionated heparin can also be used for the initial treatment of established VTE in patients with cancer (grade 2D).	**C**	Cancer	None	FDP
			For the treatment of symptomatic catheter-related thrombosis in patients with cancer, anticoagulant treatment is recommended for a minimum of 3 months; in this setting, LMWHs are suggested.	**C**	Cancer; Catheter_related_ thrombosis; Treatment_duration; CVC	None	LMWH
			For the treatment of symptomatic catheter-related thrombosis in patients with cancer, anticoagulant treatment is recommended for a minimum of 3 months; in this setting, LMWHs are suggested. Direct comparisons between LMWHs and VKAs have not been made in this setting (guidance).	**C**	Cancer; Catheter_related_ thrombosis; Treatment_duration; CVC	LMWH over VKA	LMWH
			For the treatment of established VTE in patients with cancer with a brain tumour, we prefer low-molecular-weight heparin (LMWH; guidance).	**C**	Cancer; Brain_tumour	None	LMWH

			LMWHs are preferred over vitamin K antagonists (VKAs) for the treatment of VTE in patients with cancer (grade 1A).	**A**	Cancer	LMWH over VKA	LMWH
			LMWH should be used for a minimum of 3 months to treat established VTE in patients with cancer (grade 1A).	**A**	Cancer; Treatment_duration	None	LMWH
			After 3–6 months, termination or continuation of anticoagulation (LMWH, VKA, or direct oral anticoagulants) should be based on individual assessment of the benefit-to-risk ratio, tolerability, drug availability, patient preference, and cancer activity (guidance, in the absence of data).	**D**	Cancer; Treatment_duration	None	LMWH
Carrier et al. (8)	**2015**	**Moderate**	In patients with advanced cancer in complete remission with a low or moderate risk of cancer recurrence, we suggest these options: Treatment discontinuation, therapy with a lmwh until the risk of cancer or vte recurrence is felt to be low, substitution therapy with warfarin, therapy with a doac	**D**	Cancer; Remission; Cancer_recurrence; Low_risk_VTE_recurrence	None	LMWH
			In patients with advanced cancer in complete remission for whom the short-term risk of cancer recurrence is high, or in the presence of other ongoing major risk factors for thrombosis, we recommend continuation of anticoagulant therapy as a reasonable option. In such situations, the continuation of lmwh could be preferable to other alternatives. [Level of evidence: v; Level of agreement: 62% (n = 13) strongly agreed, 24% (n = 5) somewhat agreed, 14% (n = 3) somewhat disagreed]	**D**	Cancer; Remission; Cancer_recurrence; High_risk_VTE	LMWH over 'other alternatives'	LMWH
			We recommend that continuation of anticoagulation with the most appropriate agent is required in most circumstances if an indication for anticoagulation was present before the incident cancer (for example, in cases of atrial fibrillation or previous vte not felt to be related to malignancy). Reasonable options for therapy include well controlled warfarin, lmwh, and doacs.	**D**	Cancer	None	LMWH
			Appropriate anticoagulation therapy options for catheter-related cancer-associated thrombosis include lmwh monotherapy and lmwh overlapped with warfarin. Most experts favour the use of lmwh monotherapy	**B**	Cancer; Cancer_related_thrombosis; CVC	LMWH monotherapy over Warfarin	LMWH

			In patients with active cancer and a history of VTE who develop an objectively confirmed VTE recurrence during active anticoagulation with a DOAC (for example, apixaban, dabigatran, rivaroxaban, edoxaban), we recommend switching to full-dose LMWH for a minimum of 4 weeks; expert consensus would recommend long-term therapy. [Level of evidence: v]	**D**	Cancer; Failed_treatment; VTE_recurrence; Treatment_duration	None	LMWH
			We suggest that lmwh should be used with extreme care in patients with end-stage renal disease requiring dialysis. Use should ideally be confined to research studies in the setting of anti– factor Xa monitoring. [Level of evidence: v; Level of agreement: 52% (n = 11) strongly agreed, 38% (n = 8) somewhat agreed, 10% (n = 2) strongly disagreed]	**D**	Cancer; Renal_function	None	LMWH
			In the absence of a contraindication to anticoagulation, we suggest continuation of anticoagulant therapy beyond 6 months as the preferred option in patients with active advanced cancer. Although no data are available to guide selection of therapy, continuation of lmwh at the established dose is the preferred option for most situations.	**D**	Cancer; Advanced_cancer; Treatment_duration	None	LMWH
Kearon et al. (9)	**2016**	**High**	In patients with DVT of the leg or PE and cancer ("cancer-associated thrombosis"), as long-term (first 3 months) anticoagulant therapy, we suggest LMWH over VKA therapy (Grade 2C), dabigatran (Grade 2C), rivaroxaban (Grade 2C), apixaban (Grade 2C), or edoxaban (Grade 2C).	**C**	Cancer; Cancer_associated_thrombosis; Treatment_duration	LMWH over VKAs and dabigatran, rivaroxaban, apixaban, or edoxaban	LMWH

Streiff et al. (10)	**2011**	**Moderate**	Therapeutic anticoagulation treatment for venous thromboembolism: Acute Management (at Diagnosis or During Diagnostic Evaluation)<br><br>Dalteparin (200 units/kg subcutaneous daily); Enoxaparin (1 mg/kg subcutaneous every 12 hours); Tinzaparin (175 units/kg subcutaneous daily); Fondaparinux (5 mg [< 50 kg]; 7.5 mg [50-100 kg]; 10 mg [> 100 kg] subcutaneous daily); Unfractionated heparin (IV) (80 units/kg load, then 18 units/kg/h, target aPTT of 2-2.5 x control or per hospital SOP)	**C**	Cancer	None	LMWH, FDP, Dalteparin… Enoxaparin…Tinzaparin…
			Although each of the LMWHs have been studied in randomized controlled trials in cancer patients, the efficacy of dalteparin in this population is supported by the highest quality evidence and it is the only LMWH approved by the FDA for this indication	**C**	Cancer	Dalteparin preferred over other LMWH	LMWH, dalteparin
			Tinzaparin should be avoided in patients > 70 y with renal insufficiency.	**C**	Cancer; Renal_function; Contraindicated	None	Tinzaparin
			Fondaparinux is contraindicated in patients with C_{cr} < 30 mL/min. It should be used with caution in patients with moderate renal insufficiency (30-50 mL/min), weight < 50 kg, or age > 75 y.	**C**	Cancer; Renal_function; Bodyweight; Older_adults	None	FDP
			LMWHs should be used with caution in patients with renal dysfunction	**C**	Cancer; Renal_function	None	LMWH
Keeling et al. (11)	**2011**	**Low**	Patients with cancer-associated VTE should initially be treated for 6 months with therapeutic dose LMWH rather than warfarin (1A).	**A**	Cancer; Cancer_associated_thrombosis; Treatment_duration	LMWH over Warfarin	LMWH

Lyman et al. (12)	**2015**	**High**	LMWH is preferred over UFH for the initial 5 to 10 days of anticoagulation for the cancer patient with newly diagnosed VTE who does not have severe renal impairment (defined as creatinine clearance < 30 mL/min).	**A**	Cancer; Renal_function; Treatment_duration	LMWH over UFH	LMWH
			For long term anticoagulation, LMWH for at least 6 months is preferred due to improved efficacy over Vitamin K antagonists. Vitamin K antagonists are an acceptable alternative for long-term therapy if LMWH is not available.	**A**	Cancer; Treatment_duration	LMWH over VKA	LMWH
			Anticoagulation with LMWH or Vitamin K antagonist beyond the initial 6 months may be considered for select patients with active cancer, such as those with metastatic disease or those receiving chemotherapy.	**C**	Cancer; Chemotherapy; Treatment_duration	None	LMWH
Liu et al. (13)	**2015**	**Moderate**	For patients with acute iliofemoral DVT and cancer, low-molecular-weight heparin is suggested (I, B, strong, moderate).	**B**	Cancer	None	LMWH
Watson et al. (14)	**2015**	**Low**	In the presence of active malignancy, anticoagulation should be continued, taking patient status and wishes and bleeding risk into consideration. There is a rationale but little direct evidence for preferring to continue to use LMWH (2B).	**B**	Cancer	None	LMWH
			Warfarin and other oral anticoagulants are acceptable alternatives if LMWH is impractical and anticoagulation is indicated (1A).	**A**	Cancer; Resources	None	LMWH
			In patients with cancer-associated thrombosis initial treatment should be with LMWH for 6 months, if tolerated (1A).	**A**	Cancer; Cancer_associated_thrombosis; Treatment_duration	None	LMWH

Easaw et al. (15)	**2015**	**Moderate**	In patients with established vte, low molecular weight heparin (lmwh) is the treatment of choice because of decreased recurrence rates on treatment.	**A**	Cancer	LMWH preferred	LMWH
			Warfarin (inr 2–3), although less favoured, can be used in situations in which lmwh is contraindicated or the patient refuses lmwh	**A**	Cancer; Contraindicated; Patient_refusal	LMWH over Warfarin	LMWH
			Strongly suspected or confirmed hit, whether complicated by thrombosis or not, should be treated with a non-heparin agent. Health Canada–approved agents include lepirudin, argatroban, and danaparoid. Off-label agents include bivalirudin and fondaparinux.	**A**	Cancer; HIT	None	FDP
			In patients with significant thrombocytopenia, lmwh or ufh is preferred over vitamin K agonist if anticoagulation is necessary	**D**	Cancer; Thrombocytopenia	LMWH or UFH over VKA	LMWH
			There is no high-level evidence to recommend one lmwh or ufh over another in patients with impaired renal function. Enoxaparin might have a less favourable biologic profile than tinzaparin and dalteparin in patients with impaired renal function.	**B**	Cancer; Renal_function	tinzaparin and dalteparin over enoxaparin	LMWH, enoxaparin, tinzaparin, dalteparin
			There is no high level evidence to recommend one lmwh or unfractionated heparin (ufh) over another in elderly patients with active malignancy. Tinzaparin might have a favourable biologic profile using therapeutic dosing in the setting of renal insufficiency.	**B**	Cancer; Renal_function; Older_adults	tinzaparin over other LMWH or UFH	LMWH, tinzaparin
			In patients with active malignancy who are undergoing hemodialysis, lmwh should not routinely be used and should be administered only after consultation with a nephrologist.	**C**	Cancer; Renal_function	None	LMWH
**Debourdeau *et al.*** (16)	**2013**	**High**	For the treatment of symptomatic CRT, anticoagulant treatment (AC) is recommended for a minimum of 3 months; in this setting, LMWHs are suggested.	**D**	Cancer; Catheter_related_thrombosis; Treatment_duration; CVC	None	LMWH
Mandala et al. (17)	**2011**	**Low**	In patients with severe renal failure (creatinine clearance <25-30 ml), UFH i.v. or LMWH with anti-Xa activity monitoring is recommended [I, A].	**A**	Cancer; Renal_function	None	LMWH

Table 5: Recommendations for the treatment of VTE in patients without cancer

Guideline Identification	Publication Year	CPG Quality	Recommendation	Standardized Level of Evidence	Thematic Code	Comparative Preference	Medications Discussed
Scottish Intercollegiate Guidelines Network (SIGN) (1)	**2013**	**High**	LMWH should be used with caution for those in whom standard or weight- adjusted dosing is likely to be unreliable, especially in….. patients with acute kidney injury or stage 4-5 chronic kidney disease.	**C**	Bodyweight; Renal_function	None	LMWH
			LMWH should be used with caution for those in whom standard or weight- adjusted dosing is likely to be unreliable, especially in…patients in extreme weight ranges.	**C**	Bodyweight	None	LMWH
Venous Thromboembolism Guideline Team (University of Michigan) (3)	**2014**	**Low**	LMWH is appropriate for most patients with DVT to use at home [IIA].	B	Outpatients	None	LMWH
			When warfarin is chosen as an oral agent, continue heparin (LMWH or UFH) until either INR is optimally > 2.0 or for at least five days to minimize risk of extension of thrombosis or occurrence/recurrence of embolism [IB].	C	Minimize_risk; Treatment_duration	None	LMWH
			LMWH is preferred for initial treatment over unfractionated heparin (UFH) or fondaparinux due to better safety and outcomes [IA].	**B**	Outpatients	LMWH over UFH or FDP	LMWH, FDP
Cardiovascular Disease Educational and Research Trust et al. (4)	**2013**	**Moderate**	LMWH for 3-6 months is an alternative to VKA therapy (level of evidence: high).	**A**	General_treatment; Treatment_duration	None	LMWH
			Rivaroxaban or dabigatran are an alternative therapy in countries where they have been approved (level of evidence: high). While the former can be used as a single therapy, the latter should be preceded by one week of parenteral anticoagulation with either LMWH or fondaparinux.	**A**	General_treatment	None	LMWH, FDP
			VKA therapy should be commenced on day one and continued according to the INR. Initial therapy with LMWH, IV UFH or Fondaparinux should be discontinued when the stable INR is in the therapeutic range (2.0-3.0) (level of evidence: high).	**A**	General_treatment	None	LMWH, FDP

			Due to the strong hypercoagulable state and high risk of thrombosis associated with HIT, it is recommended that all HIT patients be treated with a non-heparin anticoagulant such as argatroban, lepirudin, or danaparoid (level of evidence: moderate)…Fondaparinux may be considered as a second-line agent in the management of patients with suspected HIT (level of evidence: low).	**B**	HIT; High_risk_for_thrombosis	DOACs over FDP	FDP
			LMWH is contraindicated in patients with HIT (level of evidence: moderate).	**B**	HIT; Contraindicated	None	LMWH
Kearon et al. (9)	**2016**	**High**	For patients with DVT of the leg or PE and no cancer who are not treated with dabigatran, rivaroxaban, apixaban, or edoxaban, we suggest VKA therapy over low-molecular weight heparin (LMWH) (Grade 2C).	**C**	General_treatment	VKA over LMWH	LMWH
Liu et al. (13)	**2015**	**Moderate**	For patients with acute iliofemoral DVT and without cancer, treatment with the following alternative regimens may be initiated: low-molecular weight heparin, with switch after 1 week to dabigatran; rivaroxaban; or apixaban (I, B, strong, moderate).	**B**	Iliofemoral_DVT; General_treatment	None	LMWH
Linkins et al. (18)	**2012**	**High**	In patients with HITT, we recommend the use of nonheparin anticoagulants, in particular lepirudin, argatroban, and danaparoid, over the further use of heparin or LMWH or initiation/ continuation of VKA (Grade 1C)	**C**	HITT; General_treatment	Non-heparin anticoagulants over LMWH	LMWH
			In patients with a past history of HIT who have acute thrombosis (not related to HIT) and normal renal function, we suggest the use of fondaparinux at full therapeutic doses until transition to VKA can be achieved (Grade 2C)	**C**	HIT; Renal_function	None	FDP
Holbrook et al. (19)	**2012**	**High**	For patients with acute VTE, we suggest that VKA therapy be started on day 1 or 2 of LMWH or UFH therapy rather than waiting for several days to start (Grade 2C)	**C**	General_treatment	None	LMWH

Table 6: Recommendations for peri-operative bridging for patients who require long-term warfarin and must discontinue due to surgery

Guideline Identification	Publication Year	CPG Quality	Recommendation	Standardized Level of Evidence	Thematic Code	Comparative Preference	Medications Discussed
Cardiovascular Disease Educational and Research Trust et al. (4)	**2013**	**Moderate**	In patients with MHV and AF at high arterial thromboembolic risk or patients with VTE at high VTE risk, bridging therapy with LMWH or UFH in the periprocedural period during temporary interruption of VKA should be considered (level of evidence: low). LMWH should be preferred over UFH.	B	High_risk_for_thrombosis; Bridging; Surgery	LMWH over UFH	LMWH
			In patients undergoing a minor invasive or surgical procedure, bridging anticoagulation with LMWH should be resumed within 24 hours after the procedure if there is adequate hemostasis (level of evidence: low).	B	Minor_invasive_procedure; minor_surgery; Hemostasis	None	LMWH
			In patients undergoing major surgery or high-bleeding risk procedures, consider one of three options: 1) delay LMWH approximately 48-72 hours after surgery until hemostasis is achieved; 2) administer low-dose LMWH (usually within 24 h after a procedure); or 3) avoid post-procedural bridging therapy altogether (level of evidence: low).	B	Major_surgery; High_risk_for_bleeding; Bridging	None	LMWH
			LMWH should be used in the outpatient setting as bridging therapy over in-hospital UFH to avoid hospitalization (level of evidence: low).	B	Outpatients; Bridging; Surgery	None	LMWH
Keeling et al. (11)	**2011**	**Low**	Patients with VTE more than 3 months earlier can be given prophylactic dose LMWH (or a suitable alternative) rather than bridging therapy (2C).	C	Bridging; Surgery	None	LMWH
Whitlock et al. (20)	**2012**	**High**	In patients with mechanical heart valves, we suggest bridging with unfractionated heparin (UFH, prophylactic dose) or LMWH (prophylactic or therapeutic dose) over IV therapeutic UFH until stable on VKA therapy (Grade 2C).	C	Mechanical_heart_valves; Bridging; Surgery	LMWH or prophylactic dose UFH over IV therapeutic dose UFH	LMWH

Douketis et al. (21)	**2012**	**High**	In patients who are receiving bridging anticoagulation with therapeutic-dose SC LMWH, we suggest administering the last preoperative dose of LMWH approximately 24 h before surgery instead of 12 h before surgery (Grade 2C).	**C**	Bridging; Surgery; Pre_operative	None	LMWH
			In patients who are receiving bridging anticoagulation with therapeutic-dose SC LMWH and are undergoing high-bleeding-risk surgery, we suggest resuming therapeutic-dose LMWH 48 to 72 h after surgery instead of resuming LMWH within 24 h after surgery (Grade 2C).	**C**	Bridging; High_risk_for_ bleeding; Surgery	None	LMWH

Table 7: Recommendations for the prevention of VTE in patients with cancer

Guideline Identification	Publication Year	CPG Quality	Recommendation	Standardized Level of Evidence	Thematic Code	Comparative Preference	Medications Discussed
Cardiovascular Disease Educational and Research Trust et al. (4)	**2013**	**Moderate**	For acutely ill medical patients prophylaxis with LDUH 5000 IU b.d. or t.d.s. (Level of evidence: high) or LMWH (enoxaparin 40 mg o.d. or dalteparin 5000 U o.d.) (Level of evidence: high) for 6-14 days are recommended.	A	Cancer; Treatment_duration	LMWH (enoxaparin or dalteparin) over FDP (an alternative)	LMWH, enoxaparin, dalteparin
			For patients with active cancer requiring therapy (acutely ill medical patients), single daily doses of 2.5 mg of fondaparinux is an alternative (level of evidence: high).	A	Cancer	None	FDP
Streiff et al. (10)	**2011**	**Moderate**	For high-risk patients receiving highly thrombotic antiangiogenic therapy (i.e., multiple myeloma patients receiving thalidomide/lenalidomide in combination with high-dose dexamethasone [≥ 480 mg/mo] or doxorubicin or multiagent chemotherapy) or for myeloma patients with 2 or more individual or myeloma risk factors, recommended prophylaxis is LMWH (e.g., enoxaparin, 40 mg subcutaneous every 24 h) or warfarin (adjusted to INR 2-3). For low-risk myeloma patients with one or no individual or myeloma risk factors, aspirin, 81-325 mg daily, may be used. Aspirin should not be used in nonmyeloma patients for VTE prevention	C	Cancer; Antiangiogenic_therapy; Multiple_myeloma; Low_risk	None	LMWH, enoxaparin
Keeling et al. (11)	**2015**	**High**	Patients with multiple myeloma receiving thalidomide- or lenalidomide-based regimens with chemotherapy and/or dexamethasone should receive pharmacologic thromboprophylaxis with either aspirin or LMWH for lower-risk patients and LMWH for higher-risk patients.	B	Cancer; Chemotherapy; Low_risk; High_risk	None	LMWH
			Based on limited RCT data, clinicians may consider LMWH prophylaxis on a case-by-case basis in highly selected outpatients with solid tumors receiving chemotherapy.	B	Cancer; Outpatients; Solid_tumour	None	LMWH
Easaw et al. (15)	**2015**	**Moderate**	In patients with liver disease, lmwh can be used	**C**	Cancer; Liver_disease	None	LMWH

Mandala et al. (17)	**2011**	**Low**	Consider LMWH, aspirin or adjusted-dose warfarin (INR 1.5) in myeloma patients receiving thalidomide plus dexamethasone or thalidomide plus chemotherapy[II, B].	**B**	Cancer; Myeloma; Chemotherapy		
			In these patients a long-term treatment for 6 months with 75–80% (i.e. 150 U/kg once daily) of the initial dose of LMWH should be considered [II, B].	**B**	Cancer; Treatment_duration		
			For cancer patients receiving chemotherapy in the adjuvant setting, a long-term treatment for 6 months with 75–80% (i.e. 150 U/kg once daily) of the initial dose of LMWH should be adopted [II, A].	**A**	Cancer; Chemotherapy; Adjuvant_setting; Treatment_duration		
			For cancer patients achieving a complete remission of a potentially curative disease (i.e. germinal cancer) a longterm treatment for 6 months with 75–80% (i.e. 150 U/kg once daily) of the initial dose of LMWH may be considered [III, C].	C	Cancer; Remission; Treatment_duration		
Urbanek et al. (22)	**2016**	**Low**	In patients with multiple myeloma receiving thalidomide or lenalidomide along with chemotherapy and/or dexamethasone it is suggested to implement pharmacological thromboprophylaxis with aspirin or LMWH in low-risk patients or LMWH in high-risk patients [2C].	C	Cancer; Multiple_myeloma; Chemotherapy; High_risk; Low_risk		
Easaw et al. (23)	**2015**	**Low**	There is no preferred lmwh for vte prophylaxis in cancer outpatients; the choice of anticoagulant is at the discretion of the treating physician.	**D**	Cancer; Outpatients	None	LMWH
			There is no high level evidence to recommend one lmwh or ufh over another in patients with impaired renal function. Enoxaparin might have a less favourable biologic profile than tinzaparin and dalteparin in patients with impaired renal function.	**B**	Cancer; Renal_function	tinsaparin and dalteparin over enoxaparin	LMWH, enoxaparin, dalteparin, tinzaparin
			There is no high-level evidence to recommend one lmwh or unfractionated heparin (ufh) over another in elderly patients with active malignancy.	**B**	Cancer; Older_adults	None	LMWH

Siragusa et al. (24)	**2012**	**High**	Patients with lung or gastrointestinal cancer should receive nadroparin (3,800 U anti-FXa daily) for no more than 4 months (grade A)	**A**	Cancer; Lung_cancer; Gastrointestinal _cancer; Treatment_dur ation	None	nadroparin
			Patients with multiple myeloma treated with thalidomide or lenalidomide plus high-dose dexamethasone should receive LMWH or aspirin or warfarin (Grade C)	**C**	Cancer; Multiple_myelo ma; Chemotherapy	None	LMWH
Kahn et al. (25)	**2012**	**High**	In outpatients with cancer and indwelling CVCs, we suggest against routine prophylaxis with LMWH or LDUH (Grade 2B) and suggest against the prophylactic use of VKAs (Grade 2C)	**B**	Cancer; CVC	None	LMWH
			In outpatients with solid tumors who have additional risk factors for VTE and who are at low risk of bleeding, we suggest prophylactic dose LMWH or LDUH over no prophylaxis (Grade 2B)	**B**	Cancer; Solid_tumour; Low_risk	None	LMWH
			In outpatients with cancer who have no additional risk factors for VTE, we suggest against routine prophylaxis with LMWH or LDUH (Grade 2B) and recommend against the prophylactic use of vitamin K antagonists (Grade 1B)	**B**	Cancer; Outpatients	None	LMWH

Table 8: Recommendations for the prevention of VTE in patients undergoing non-orthopedic surgery

Guideline Identification	Publication Year	CPG Quality	Recommendation	Standardized Level of Evidence	Thematic Code	Comparative Preference	Medications Discussed
Scottish Intercollegiate Guidelines Network (SIGN) (1)	**2013**	**High**	Further treatment with LMWH after bypass surgery is not recommended	**B**	Bypass_surgery; Post_operative	None	LMWH
Cardiovascular Disease Educational and Research Trust et al. (4)	**2013**	**Moderate**	High- risk patients are those over the age of 60 undergoing major surgery for benign disease or any patient with additional risk factors: LMWH or fondaparinux initiated and dosed according to labelling is recommended (level of evidence: high).	**A**	High_risk; Major_surgery; Older_adults	None	LMWH, FDP
			High- risk patients are those over the age of 60 undergoing major surgery for benign disease or any patient with additional risk factors: In the absence of LMWH or fondaparinux, LDU H 5000 IU commenced preoperatively and continued twice or three times daily can be used (level of evidence: high).	**A**	High_risk; Older_adults; Major_surgery; Pre_operative	LMWH or FDP over LDUH	LMWH, FDP
			High- risk patients are those over the age of 60 undergoing major surgery for benign disease or any patient with additional risk factors: LMWH or fondaparinux initiated and dosed according to labelling is recommended (level of evidence: high).	**A**	High_risk; Major_surgery; Older_adults	None	LMWH, FDP
			Moderate-risk patients are those over the age of 40 years undergoing major surgery for benign disease in the absence of additional risk factors.<br><br>The use of LMWH (initiated and dosed according to labelling) or LDUH is recommended (level of evidence: high). However, LMWH is the preferred option because it is administered as one injection daily and is associated with a lower incidence of HIT.	**A**	Major_surgery; HIT; Middle_aged; Moderate_risk	LMWH over LDUH	**LMWH**

			In surgical patients with cancer, LDUH (5000 IU 8 h commenced prior to operation) (level of evidence: high) or LMWH (initiated and dosed according to manufacturer's recommendations) (level of evidence: high) should be used.	**A**	Cancer; Cancer_surgery	None	**LMWH**
			In the postdischarge period prolonged thromboprophylaxis with LMWH (enoxaparin, dalteparin or bemivarin) for up to four weeks after operation should be considered (level of evidence: moderate).	**B**	Surgery; Postdischarge; Post_operative; Treatment_duration	None	LMWH, enoxaparin, dalteparin or bemivarin
			Consideration should be given to continuing thromboprophylaxis after hospital discharge with LMWH for up to 28 days especially in patients with cancer (level of evidence: low) extrapolated from general surgery.	**B**	Cancer; Surgery; Postdischarge; Post_operative; Treatment_duration	None	LMWH
			Patients undergoing abdominal or pelvic major surgery for cancer and do not present contraindications to extended prophylaxis should receive LMWH up to one month after operation (level of evidence: high).	**A**	Cancer; Major_surgery; Abdominal_pelvic_surgery; Post_operative; Treatment_duration	None	LMWH
			Patients undergoing abdominal or pelvic major surgery for cancer and do not present contraindications to extended prophylaxis should receive LMWH up to one month after operation (level of evidence: high).	**A**	Cancer; Major_surgery; Cancer_surgery; Abdominal_pelvic_surgery; Post_operative; Treatment_duration	None	LMWH
			High-risk patients: Major gynecologic surgery, age >60 — Major gynecologic surgery, age 40-60 and cancer or history of DVT/PE or other risk factors including thrombophilia<br><br>LMWH (initiated and dosed according to labeling) (level of evidence: high)	**A**	Cancer; High_risk; Major_surgery; Gynecologic_surgery; Middle_aged; Older_adults	None	LMWH
			High-risk patients: Major gynecologic surgery, age >60 — Major gynecologic surgery, age 40-60 and cancer or history of DVT/PE or other risk factors including thrombophilia<br><br>Fondaparinux (level of evidence: low)	**B**	Cancer; High_risk; Major_surgery; Gynecologic_surgery; Thrombophilia; Middle_aged; Older_adults	None	FDP

			Moderate-risk patients: Major gynecologic surgery, age 40-60 and cancer or Major gynecologic surgery, age 40-60 without other risk factors — Major gynecologic surgery, age 40-60 and cancer or Minor gynecologic surgery, age 60<br><br>LDUH (5000 IU, 12 h), LMWH (initiated and dosed according to labeling) or IPC are recommended (level of evidence: high).	**A**	Cancer; Moderate_risk; Major_surgery; Gynecologic_surgery; Middle_aged; Older_adults; Minor_surgery	None	LMWH
			For patients undergoing urologic surgery: LDUH is recommended (level of evidence: high) or LMWH extrapolated from trials in patients having general surgery (level of evidence:low).	**B**	Urologic_surgery	LDUH over LMWH	LMWH
			Patients undergoing laparoscopic surgery…in the presence of additional risk factors they should receive LDUH, LMWH, fondaparinux or IPC with GEC (level of evidence: low)	**B**	Laparoscopic_surgery	None	LMWH, FDP
			Patients undergoing bariatric surgical procedures should receive LMWH (higher dosage) alone or in combination with GEC and IPC (level of evidence: moderate).	**B**	Bariatric_surgery	None	LMWH
			Patients undergoing major vascular procedures should receive LMWH or fondaparinux (level of evidence: low).	**B**	Surgery; Major_vascular_procedures	None	LMWH, FDP
			High risk patients having plastic surgery should receive LMWH, fondaparinux starting 24 hours after surgery or a combination of LMWH with IPC and GES (level of evidence: low).	**B**	High_risk; Plastic_surgery; Post_operative	None	LMWH, FDP

Farge et al. (7)	**2016**	**High**	For the prophylaxis of VTE in surgically treatment patients with cancer: Use of low-molecular-weight heparin (LMWH) once per day or low-dose unfractionated heparin (UFH) three times per day is recommended to prevent postoperative VTE in patients with cancer; pharmacological prophylaxis should be started 12–2 h preoperatively and continued for at least 7–10 days; no data are available to allow conclusions regarding the superiority of one type of LMWH over another (grade 1A).	**A**	Cancer; Cancer_surgery; Post_operative; Pre_operative; Treatment_duration	No superiority of one LMWH over another	LMWH
			For the prophylaxis of VTE in surgically treatment patients with cancer: Evidence to support fondaparinux as an alternative to LMWH for the prophylaxis of postoperative VTE in patients with cancer is insufficient (grade 2C).	**C**	Cancer; Cancer_surgery; Post_operative	None	LMWH, FDP
			For patients with brain tumors: We recommend the use of LMWH or unfractionated heparin (UFH) started postoperatively for the prevention of VTE in patients with cancer undergoing neurosurgery (grade 1A)	**A**	Cancer; Neurosurgery; Post_operative	None	LMWH
			For the prophylaxis of VTE in surgically treatment patients with cancer: Extended prophylaxis (4 weeks) with LMWH to prevent postoperative VTE after major laparotomy in patients with cancer is indicated in patients with a high VTE risk and low bleeding risk (grade 1B).	**B**	Cancer; Cancer_surgery; High_risk; Low_risk; Post_operative; Treatment_duration	None	LMWH
			For the prophylaxis of VTE in surgically treatment patients with cancer: Extended prophylaxis (4 weeks) with LMWH for the prevention of VTE in patients with cancer undergoing laparoscopic surgery is recommended in the same way as for laparotomy (grade 2C)	**C**	Cancer; Cancer_surgery; Laparotomy; Laparoscopic_surgery; Treatment_duration	None	LMWH

Keeling et al. (11)	**2015**	**High**	All patients with malignant disease undergoing major surgical intervention should be considered for pharmacologic thromboprophylaxis with either UFH or LMWH unless contraindicated because of active bleeding or a high bleeding risk.	**A**	Cancer; Major_surgery; Cancer_Surgery; Contraindicated; High_risk	None	LMWH
			Extended prophylaxis with LMWH for up to 4 weeks postoperatively should be considered for patients undergoing major abdominal or pelvic surgery for cancer who have high-risk features such as restricted mobility, obesity, history of VTE, or with additional risk factors. In lower risk surgical settings, the decision on appropriate duration of thromboprophylaxis should be made on a case-by-case basis considering the individual patient.	**A**	Cancer; Postoperative; Major_surgery; Cancer_surgery; Abdominal_pelvic_surgery; Bodyweight; High_risk; Low_risk; Restricted_mobility; Post_operative; Treatment_duration	None	LMWH
Mandala et al. (17)	**2011**	**Low**	In cancer patients undergoing major cancer surgery, prophylaxis with LMWHs or UFH is recommended. Mechanical methods such as pneumatic calf compression may be added to pharmacological prophylaxis but should not be used as monotherapy unless pharmacological prophylaxis is contraindicated because of active bleeding [I, A].	**A**	Cancer; Major_Surgery; Cancer_surgery	None	LMWH
			Cancer patients undergoing elective major abdominal or pelvic surgery should receive in hospital and post-discharge prophylaxis with s.c. LMWH for up to 1 month after surgery [I, A].	**A**	Cancer; Major_surgery; Abdominal_pelvic_surgery; Postdischarge; Post_operative; Treatment_duration	None	LMWH

Urbanek et al. (22)	**2016**	**Low**	Patients suffering from active cancer and at high risk of VTE undergoing other surgeries, who are not at high risk of serious bleeding complications, should receive pharmacological prophylaxis with low-molecular-weight heparin in the dose that is adequate to the VTE risk or low doses of unfractionated heparin (5000 U every 8 h s.c.) [1A].	**A**	Cancer; Surgery	None	LMWH
			If bleeding risk is not high in cancer patients with moderate VTE risk undergoing other surgical procedures than extensive surgery, it is recommended to administer thromboprophylaxis with low-molecular-weight heparin or low doses of unfractionated heparin according to the current risk assessment for VTE and up-to-date drug authorizations [1B].	**B**	Cancer; Surgery	None	LMWH
			In patients undergoing intracranial oncological neurosurgery related to high or very high risk of venous thromboembolism, who are not at high risk for bleeding, in the postoperative period the pharmacological thromboprophylaxis by the means of low doses of unfractionated heparin or low molecular heparin should be added to the mechanical methods of prophylaxis [2C].	**C**	Cancer; Cancer_surgery; Intracranial_surgery; Post_operative	None	LMWH
			All patients undergoing extensive oncological surgeries in the abdomen and pelvis should receive VTE prophylaxis with prophylactic doses of low-molecular-weight heparin or low doses of unfractionated heparin (3 × 5000 U), if there are no contraindications, including active bleeding or high risk of bleeding events [1A].	**A**	Cancer; Surgery; Cancer_surgery; Abdominal_pelvic_surgery; High_risk	None	LMWH
			Patients undergoing major cancer surgery in the abdominal cavity and/or pelvis, who are not at high risk of serious bleeding complications and have VTE risk factors such as prolonged immobilization, obesity, history of VTE, or other DVT risk factors, are recommended to receive prolonged pharmacological prophylaxis (4 weeks) with low-molecular-weight heparin [2B].	**B**	Cancer; Surgery; Major_surgery; Cancer_surgery; Bodyweight; Treatment_duration	None	LMWH

			In patients undergoing major urologic surgeries in the abdominal cavity and/or pelvis, who are not at high risk of serious bleeding complications it is suggested to prolong pharmacological prophylaxis (4 weeks) with low-molecular-weight heparin [2C].	**C**	Major_surgery; Urologic_surgery; Abdominal_pelvic_surgery; Treatment_duration	None	LMWH
			In cases of moderate risk of VTE and when there is no high risk of serious bleeding complications in cancer patients undergoing thoracic surgery, it is recommended to use LMWH [2B], LDUH [2B] or mechanical methods (most preferably intermittent pneumatic compression — IPC) [2C] more than not using any prophylaxis at all.	**B**	Cancer; Cancer_surgery; Thoracic_surgery	None	LMWH
			In the case of high risk of VTE and when there is low high risk of serious bleeding complications in patients undergoing thoracic cancer surgery, it is recommended to use LMWH [1B] or LDUH [1B] more than not using any pharmacological prophylaxis at all.	**C**	Cancer; Cancer_surgery; low_risk; high_risk	None	LMWH
			In cancer patients undergoing major oncological surgery due to gynecological indications in the area of the pelvis and abdominal cavity, VTE thromboprophylaxis with prophylactic doses of low molecular heparin or low doses of unfractionated heparin is recommended, as long as there are no contraindications which include active bleeding or a high risk of bleeding complications [1A].	**A**	Cancer; Cancer_surgery; Gynecologic_surgery; Abdominal_pelvic_surgery	None	LMWH
			VTE prophylaxis in gynecological cancer surgery: Unless there is a high risk of serious bleeding complications in patients with cancer and a moderate risk of venous thromboembolism undergoing surgery other than major surgery it is recommended to administer VTE prophylaxis by the means of low-molecular-weight heparin or low doses of unfractionated heparin according to the current VTE risk assessment evaluation [1B].	**B**	Cancer; Cancer_surgery; Gynecologic_surgery	None	LMWH

			In patients undergoing major gynecological cancer surgery, who are not at a high risk of serious bleeding complications with the presence of VTE risk factors such as prolonged immobilization, obesity, history of previous VTE or other thrombosis risk factors, it is suggested to use the prolonged pharmacological prophylaxis (4 weeks) with low molecular weight heparin. [2C]	**C**	Cancer; Major_surgery; Cancer_surgery; Gynecologic_surgery; Treatment_duration	None	LMWH
			In patients undergoing major urological surgery due to cancer and in those undergoing other urological procedures, who are at high risk of venous thromboembolism, it is recommended to consider the use of thromboprophylaxis based on LDUH [1B] or LMWH [1C], if the risk of bleeding do not significantly outstands potential benefits associated with the use of pharmacoprophylaxis.	**C**	Cancer; Major_surgery; Cancer_surgery; Urologic_surgery; High_risk	None	LMWH

Siragusa et al. (24)	**2012**	**High**	In patients undergoing surgery for cancer, pharmacological prophylaxis with UFH, LMWH or fondaparinux should be given for at least 7 days (grade A)	**A**	Cancer; Cancer_surgery; Treatment_duration	None	LMWH, FDP
			In cancer patients undergoing surgery, pharmacological prophylaxis with unfractionated heparin (UFH) or LMWH should be administered preoperatively (grade C)	**C**	Cancer; Cancer_surgery; Pre_operative	None	LMWH
			In cancer patients undergoing surgery with major abdominal or pelvic surgery, pharmacological prophylaxis with heparin or fondaparinux should be continued for 4 weeks (grade A)	**A**	Cancer; Major_surgery; Abdominal_pelvic_surgery; Treatment_duration	None	FDP
Gould et al. (26)	**2012**	**High**	For thoracic surgery patients at moderate risk for VTE who are not at high risk for perioperative bleeding, we suggest LDUH (Grade 2B) , LMWH (Grade 2B) , or mechanical prophylaxis with optimally applied IPC (Grade 2C) over no prophylaxis	**B**	Thoracic_surgery; Moderate_risk	None	LMWH
			For thoracic surgery patients at high risk for VTE who are not at high risk for perioperative bleeding, we suggest LDUH (Grade 1B) or LMWH (Grade 1B) over no prophylaxis.	**B**	Thoracic_surgery; High_risk	None	LMWH
			For general and abdominal-pelvic surgery patients at moderate risk for VTE (3.0%; Rogers score, . 10; Caprini score, 3-4) who are not at high risk for major bleeding complications, we suggest low-molecular-weight heparin (LMWH) (Grade 2B), low-dose unfractionated heparin (LDUH) (Grade 2B) , or mechanical prophylaxis, preferably with IPC (Grade 2C) , over no prophylaxis.	**B**	General_surgery; Abdominal_pelvic_surgery	None	LMWH
			For general and abdominal-pelvic surgery patients at high risk for VTE (6.0%; Caprini score, 5) who are not at high risk for major bleeding complications, we recommend pharmacologic prophylaxis with LMWH (Grade 1B) or LDUH (Grade 1B) over no prophylaxis. We	**B**	General_surgery; Abdominal_pelvic_surgery	None	LMWH

			For general and abdominal-pelvic surgery patients at high risk for VTE (6%; Caprini score, 5) in whom both LMWH and unfractionated heparin are contraindicated or unavailable and who are not at high risk for major bleeding complications, we suggest low-dose aspirin (Grade 2C) , fondaparinux (Grade 2C) , or mechanical prophylaxis, preferably with IPC (Grade 2C) , over no prophylaxis.	**C**	General_surgery; Abdominal_pelvic_surgery; Contraindicated; High_risk	None	LMWH, FDP
			For high-VTE-risk patients undergoing abdominal or pelvic surgery for cancer who are not otherwise at high risk for major bleeding complications, we recommend extended-duration pharmacologic prophylaxis (4 weeks) with LMWH over limited-duration prophylaxis (Grade 1B) .	**B**	Cancer; Abdominal_pelvic_surgery; Cancer_surgery; Treatment_duration	None	LMWH

Table 9: Recommendations for the post-operative prophylaxis of VTE in patients undergoing surgery of the lower limbs

Guideline Identification	Publication Year	CPG Quality	Recommendation	Standardized Level of Evidence	Thematic Code	Comparative Preference	Medications Discussed
Cardiovascular Disease Educational and Research Trust et al. (4)	**2013**	**Moderate**	Patients undergoing elective hip replacement: LMWH initiated and dosed according to the manufacturer's recommendations (level of evidence: high), fondaparinux (level of evidence: high), vitamin K antagonists (VKA) (level of evidence: high), rivaroxaban (level of evidence: high), apixaban (level of evidence: high), dabigatran (level of evidence: high).	**A**	Hip_surgery	None	LMWH, FDP
			Patients undergoing elective hip surgery:<br><br>Prophylaxis with LMWH should be initiated either before or after operation depending on the adopted regimen (level of evidence: high).	**A**	Hip_surgery; Post_operative; Pre_operative	None	LMWH
			Patients undergoing elective hip surgery:<br><br>Prophylaxis should be continued for 4-6 weeks with LM WH (level of evidence: high) or fondaparinux (level of evidence: low).	**A**	Hip_surgery; Treatment_duration	None	LMWH
			Patients undergoing elective hip surgery:<br><br>Fondaparinux should be started at least 6-8 hours after surgery. Prophylaxis should be continued for 4-6 weeks with LM WH (level of evidence: high) or fondaparinux (level of evidence: low)	**B**	Hip_surgery; Post_operative; Treatment_duration	None	FDP
			Patients undergoing hip fracture surgery:<br><br>LMWH (initiated and dosed according to the manufacturer's recommendations) (level of evidence: high), fondaparinux (level of evidence: high), adjusted dose VKA (INR range 2-3) (level of evidence: high) or LDUH (level of evidence: high).	**A**	Hip_surgery	None	LMWH, FDP

			Patients undergoing elective knee surgery: LMWH (initiated and dosed according to the manufacturer's recommendations) (level of evidence: high), warfarin (although less effective) (level of evidence: high), rivaroxaban (level of evidence: high), apixaban (level of evidence: high), dabigatran (level of evidence: high) and fondaparinux (level of evidence: high).	**A**	Knee_surgery	LMWH over warfarin	LMWH, FDP
			Patients undergoing arthroscopic knee surgery: LMWH starting before or after surgery (level of evidence: moderate) or IPC in the presence of contraindications to LMWH are recommended (level of evidence: low) until full ambulation.	**B**	Knee_surgery	None	LMWH
Falck-Ytter et al. (27)	**2012**	**High**	In patients undergoing hip fracture surgery (HFS), we recommend use of one of the following rather than no antithrombotic prophylaxis for a minimum of 10 to 14 days: LMWH, fondaparinux, LDUH, adjusted-dose VKA, aspirin (all Grade 1B) , or an IPCD (Grade 1C)	**B**	Hip_surgery; Treatment_duration	None	LMWH, FDP
			In patients undergoing THA or TKA, irrespective of the concomitant use of an IPCD or length of treatment, we suggest the use of LMWH in preference to the other agents we have recommended as alternatives: fondaparinux, apixaban, dabigatran, rivaroxaban, LDUH (all Grade 2B) , adjusted-dose VKA, or aspirin (all Grade 2C)	**B**	Hip_surgery; Knee_surgery	LMWH over FDP, LDUH, VKA, or aspirin	LMWH, FDP
			In patients undergoing total hip arthroplasty (THA) or total knee arthroplasty (TKA), we recommend use of one of the following for a minimum of 10 to 14 days rather than no antithrombotic prophylaxis: low-molecular-weight heparin (LMWH), fondaparinux, apixaban, dabigatran, rivaroxaban, low-dose unfractionated heparin (LDUH), adjusted-dose vitamin K antagonist (VKA), aspirin (all Grade 1B) , or an intermittent pneumatic compression device (IPCD) (Grade 1C) .	**B**	Hip_sugery; Knee_surgery; Treatment_duration	None	LMWH, FDP

			For patients undergoing major orthopedic surgery (THA, TKA, HFS) and receiving LMWH as thromboprophylaxis, we recommend starting either 12 h or more preoperatively or 12 h or more postoperatively rather than within 4 h or less preoperatively or 4 h or less postoperatively (Grade 1B) .	**B**	Major_surgery; Hip_surgery; Knee_surgery; Post_operative; Pre_operative	None	LMWH
			In patients undergoing THA or TKA, irrespective of the concomitant use of an IPCD or length of treatment, we suggest the use of LMWH in preference to the other agents we have recommended as alternatives: fondaparinux, apixaban, dabigatran, rivaroxaban, LDUH (all Grade 2B) , adjusted-dose VKA, or aspirin (all Grade 2C)	**B**	Hip_surgery; Knee_surgery	LMWH over FDP, apixaban, dabigatran, rivaroxaban, LDUH, VKA, aspirin	LMWH, FDP

References

1. Antithrombotics: indications and management. Edinburgh, Scotland: Scottish Intercollegiate Guidelines Network (SIGN). 2013.

2. Chan W, Rey E, Kent N, Group' ViPGW, Chan W, Kent N, et al. Venous thromboembolism and antithrombotic therapy in pregnancy. J Obstet Gynaecol Can. 2014;36(6):527-53.

3. Venous Thromboembolism Guideline Team. Venous thromboembolism (VTE): Guidelines for Clincial Care Ambulatory. Ann Arbor (MI): University of Michigan, 2014 May. Report No.

4. Cardiovascular Disease Educational and Research Trust, European Venous Forum, North American Thrombosis Forum, International Union of Angiology, Union Internationale du Phlebologie. Prevention and treatment of venous thromboembolism: international consensus statement (guidelines according to scientific evidence). Clin Appl Thromb Hemost. 2013;19(2):116-225.

5. Bates SM, Greer IA, Middeldorp S, Veenstra DL, Prabulos AM, Vandvik PO, et al. VTE, thrombophilia, antithrombotic therapy, and pregnancy: Antithrombotic Therapy and Prevention of Thrombosis, 9th ed: American College of Chest Physicians Evidence-Based Clinical Practice Guidelines. Chest. 2012;141:e691S-e736S.

6. James A, Committee on Practice Bulletins- Obstetrics. Practice bulletin no. 123: thromboembolism in pregnancy. ObstetGynecol. 2011;118(3):718-29.

7. Farge D, Bounameaux H, Brenner B, Cajfinger F, Debourdeau P, Khorana AA, et al. International clinical practice guidelines including guidance for direct oral anticoagulants in the treatment and prophylaxis of venous thromboembolism in patients with cancer. The Lancet Oncology. 2016;17(10):e452-e66.

8. Carrier M, Lazo-Langer A, Shivakumar S, Tagalakis V, Gross P, Blais N, et al. Clinical challenges in patients with cancer-associated thrombosis: Canadian expert consensus recommendations. Curr Oncol. 2015;22(1):49-59.

9. Kearon C, Akl EA, Ornelas J, Blaivas A, Jimenez D, Bounameaux H, et al. Antithrombotic therapy for VTE disease: CHEST guideline and expert panel report. Chest. 2016;149(2):315-52.

10. Streiff M, Holstrom B, Ashrani A, Brockenstedt P, Chesney C, Eby C, et al. Cancer-Associated Venous Thromboembolic Disease, Version 1.2015. J Natl Compr Canc Netw. 2015;13(9):1079-95.

11. Keeling D, Baglin T, Tait C, Watson H, Perry D, Baglin C, et al. Guidelines on oral anticoagulation with warfarin - fourth edition. Br J Haematol. 2011;154(3):311-24.

12. Lyman GH, Khorana AA, Kuderer NM, Lee AY, Arcelus JI, Balaban EP, et al. Venous thromboembolism prophylaxis and treatment in patients with cancer: American Society of Clinical Oncology clinical practice guideline update. J Clin Oncol. 2013;31:2189-204.

13. Liu D, Peterson E, Dooner J, Baerlocher M, Zypchen L, Gagnon J, et al. Diagnosis and management of iliofemoral deep vein thrombosis: clinical practice guideline CMAJ. 2015;187(17):1288-96.

14. Watson HGK. Guideline on aspects of cancer-related venous thrombosis. British Journal of Haematology. 2015;170:640-8.

15. Easaw J, Shea-Budgell M, Wu C, Czaykowski P, Kassis J, Kuehl B, et al. Canadian consensus recommendations on the management of venous thromboembolism in patients with cancer. Part 2: treatment. Current Oncology. 2015;22:144-55.

16. Debourdeau P, Farge D, Beckers M, Baglin C, Bauersachs RM, Brenner B, et al. International clinical practice guidelines for the treatment and prophylaxis of thrombosis associated with central venous catheters in patients with cancer. Journal of thrombosis and haemostasis : JTH. 2013;11:71-80.

17. Mandala M, Falanga A, Roila F, ESMO Guidelines Working Group. Management of venous thromboembolism (VTE) in cancer patients: ESMO Clinical Practice Guidelines. Ann Oncol. 2011;22(Suppl6):vi85-vi92.

18. Linkins LA, Dans AL, Moores LK, Bona R, Davidson BL, Schulman S, et al. Treatment and prevention of heparin-induced thrombocytopenia: Antithrombotic Therapy and Prevention of Thrombosis, 9th ed: American College of Chest Physicians Evidence-Based Clinical Practice Guidelines. Chest. 2012;141:e495S-e530S.

19. Holbrook A, Schulman S, Witt DM, Vandvik PO, Fish J, Kovacs MJ, et al. Evidence-based management of anticoagulant therapy: Antithrombotic Therapy and Prevention of Thrombosis, 9th ed: American College of Chest Physicians Evidence-Based Clinical Practice Guidelines. Chest. 2012;141:e152S-e84S.

20. Whitlock RP, Sun JC, Fremes SE, Rubens FD, Teoh KH, Physicians American College of C. Antithrombotic and thrombolytic therapy for valvular disease: Antithrombotic Therapy and Prevention of Thrombosis, 9th ed: American College of Chest Physicians Evidence-Based Clinical Practice Guidelines. Chest. 2012;141:e576S-e600S.

21. Douketis J, Spyropoulos A, Spencer F, Mayr M, Jaffer A, Eckman M, et al. Perioperative management of antithrombotic therapy. Antithrombotic therapy and prevention of thrombosis, 9th ed: American College of Chest Physicians evidence-based clinical practice guidelines. Chest. 2012;141(2 Suppl):e326S-e50S.

22. Urbanek T, Krasinski Z, Kostrubiec M, Sydor W, Wysocki P, Antoniewicz A, et al. Venous thromboembolism prophylaxis in cancer patients - guidelines focus on surgical patients. Acta Angiologica. 2016;22(3):71-102.

23. Easaw JC, Shea-Budgell MA, Wu CM, Czaykowski PM, Kassis J, Kuehl B, et al. Canadian consensus recommendations on the management of venous thromboembolism in patients with cancer. Part 1: prophylaxis. Current Oncology. 2015;22:133-43.

24. Siragusa S, Armani U, Carpenedo M, Falanga A, Fulfaro F, Imberti D, et al. Prevention of venous thromboembolism in patients with cancer: Guidelines of the Italian Society for Haemostasis and Thrombosis (SISET). Thrombosis Research. 2012;129:e171-e6.

25. Kahn SR, Lim W, Dunn AS, Cushman M, Dentali F, Akl EA, et al. Prevention of VTE in nonsurgical patients: Antithrombotic Therapy and Prevention of Thrombosis, 9th ed: American College of Chest Physicians Evidence-Based Clinical Practice Guidelines. Chest. 2012;141:e195S-e226S.

26. Gould MK, Garcia DA, Wren SM, Karanicolas PJ, Arcelus JI, Heit JA, et al. Prevention of VTE in nonorthopedic surgical patients: Antithrombotic Therapy and Prevention of Thrombosis, 9th ed: American College of Chest Physicians Evidence-Based Clinical Practice Guidelines. Chest. 2012;141:e227S-e77S.

27. Falck-Ytter Y, Francis CW, Johanson NA, Curley C, Dahl OE, Schulman S, et al. Prevention of VTE in orthopedic surgery patients: Antithrombotic Therapy and Prevention of Thrombosis, 9th ed: American College of Chest Physicians Evidence-Based Clinical Practice Guidelines. Chest. 2012;141:e278S-e325S.
