# Supplementary material for: A systematic review of clinical practice guidelines on the use of low molecular weight heparin and fondaparinux for the treatment and prevention of venous thromboembolism: Implications for research and policy decision-making
Source: PLoS One. 2018 Nov 9;13(11):e0207410. doi: 10.1371/journal.pone.0207410 (PMC6226206; doi:10.1371/journal.pone.0207410)
Supplement: S4 Appendix — (PDF) [file pone.0207410.s004.pdf]

S4 Appendix. Summary of treatment and prevention-related recommendations by the level of evidence underpinning them and quality of CPG from which they originated.

As illustrated in **Figure 1** there was no apparent correlation between the quality of evidence underpinning recommendations for the treatment of VTE with LMWH or FDP and the quality of CPGs from which they originated. Briefly, while all recommendations for patients who fail treatment with warfarin (**Figure 1C**) were based on evidence levels B and C, all CPGs reporting recommendations for this indication were judged to be of moderate or high overall quality. No recommendations for the treatment of VTE in patients without cancer (**Figure 1A)** were based level D evidence; however, the largest proportion of level C recommendations for this indication was reported by high-quality CPGs. All guidelines reporting recommendations for the treatment of VTE in pregnant and/or lactating women (**Figure 1D**) that were based on level D evidence were judged to be of low overall quality, whereas all CPGs reporting level A recommendations for this indication were judged to be of high overall quality. For the treatment of VTE in patients with cancer (**Figure1B)**, all recommendations reported by low-quality CPGs were based on evidence level A or B. In contrast, high-quality CPGs reporting recommendations for this indication were based on all levels of evidence. No figure is shown for the treatment of VTE in patients who cannot tolerate warfarin or in whom it is contraindicated, as only one recommendation (Level B evidence, reported by a low-quality CPG) was identified.

Figure 1. Number of recommendations for treatment indications by level of evidence and CPG quality

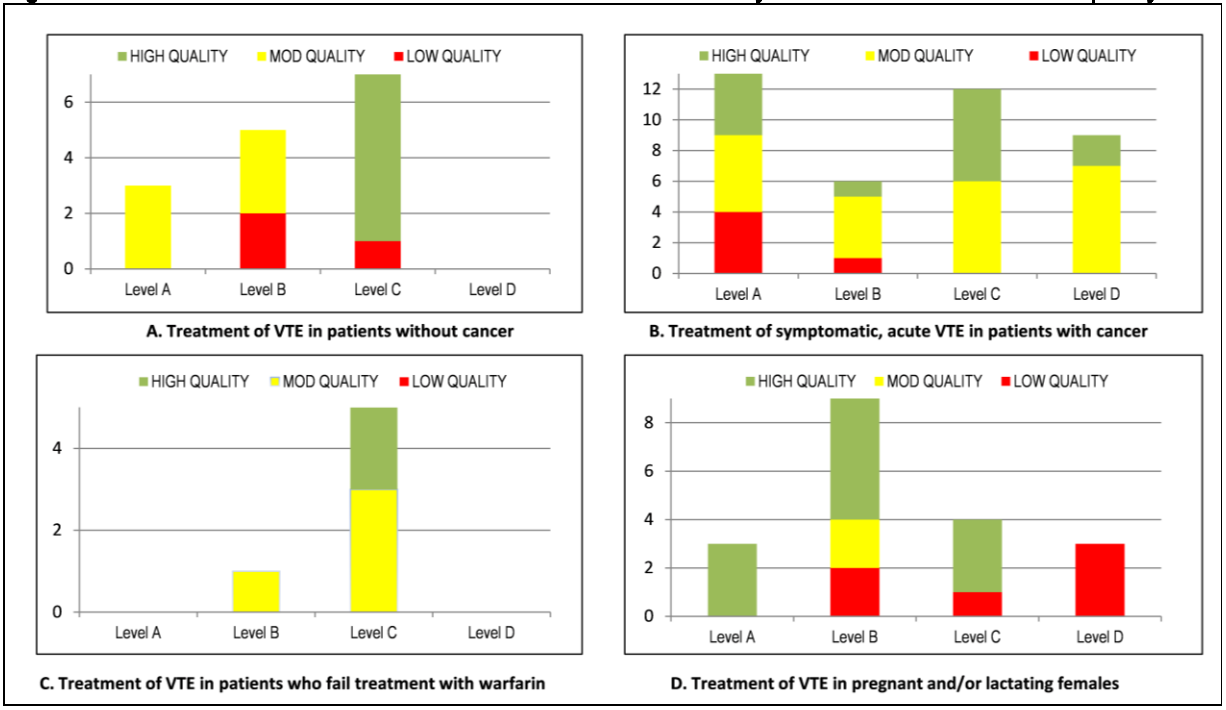

Like treatment-related indications, there was no apparent correlation between the quality of evidence underpinning recommendations for the use of LMWH or FDP in preventing VTE and the quality of CPGs from which they were extracted (**Figure 2)**. All recommendations for the post-operative prophylaxis of VTE in patients undergoing hip or knee surgery that were based on evidence level A were reported by moderate-quality CPGs (**Figure 2A**). In comparison, all recommendations for peri-operative bridging in patients needing long-term warfarin (**Figure 2D**) were based on evidence Level B and were reported by CPGs judged to be of moderate overall quality. All high-quality CPGs reporting recommendations for this indication were based on evidence Level C. For the prevention of VTE in patients with cancer, only CPGs judged to be of low-quality reported any recommendation based on evidence Level D (**Figure 2C**). Recommendations for the prevention of VTE in patients undergoing non-orthopedic surgery (**Figure 2B**) and the prevention of VTE in patients with cancer (**Figure 2C**) were reported by low, moderate, and high-quality CPGs were informed by all levels of evidence.

Figure 2. Number of prevention-related recommendations for each indication by level of evidence and CPG quality

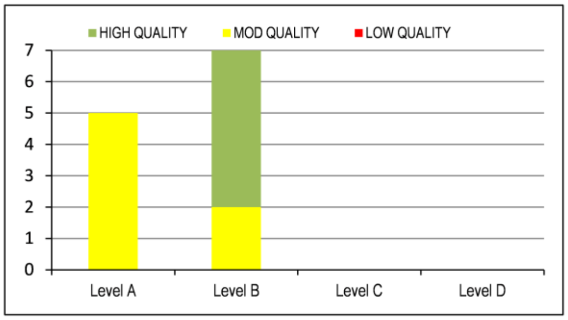

A. Post-operative prophylaxis of VTE (hip or knee); cannot use warfarin

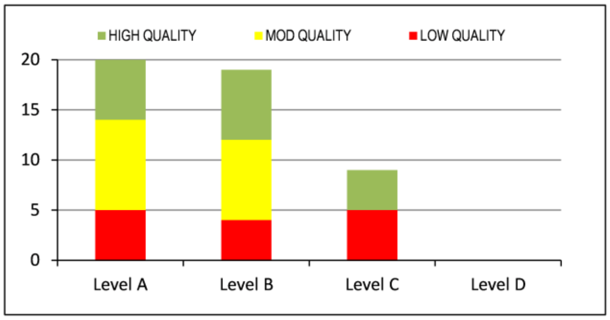

B. Prophylaxis of VTE in patients undergoing non-orthopedic surgery

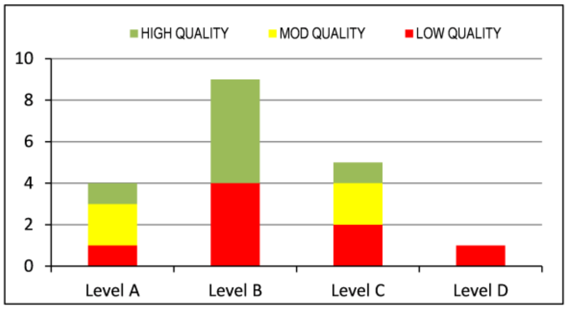

C. Prophylaxis of VTE in patients with cancer

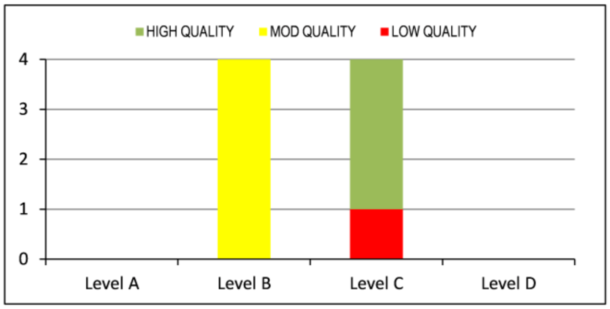

D. Peri-operative bridging of long-term warfarin
