# Supplementary material for: A systematic review of clinical practice guidelines on the use of low molecular weight heparin and fondaparinux for the treatment and prevention of venous thromboembolism: Implications for research and policy decision-making
Source: PLoS One. 2018 Nov 9;13(11):e0207410. doi: 10.1371/journal.pone.0207410 (PMC6226206; doi:10.1371/journal.pone.0207410)
Supplement: S1 Table — (PDF) [file pone.0207410.s005.pdf]

S1 Table. Evidence assessment scales used by all included clinical practice guidelines.

Last Name of First Author (Year)	Grading System	Strength of Recommendation	Level of Evidence
**Debourdeau *et al.* (2013)** [1]	Grading of Recommendations Assessment Development and Evaluation (GRADE) [2]	**Strong (Grade 1)**: The panel is confident that the desirable effects of adherence to a recommendation outweigh the undesirable effects<br>**Weak (Grade 2)**: The panel concludes that the desirable effects of adherence to a recommendation probably outweigh the undesirable effects, but is not confident<br>**Best clinical practice (Guidance):** In the absence of any clear scientific evidence and because of undetermined balance between desirable and undesirable effects, judgment was based on the professional experience and consensus of the international experts within the working group	**High (A):** Further research is very unlikely to change our confidence in the estimate of effect<br>**Moderate (B):** Further research is likely to have an important impact on our confidence in the estimate of effect and may change the estimate<br>**Low (C):** Further research is very likely to have an important impact on our confidence in the estimate of effect and is likely to change the estimate<br>**Very low (D):** Any estimate of effect is very uncertain
**Farge *et al.* (2016)** [3]	Grading of Recommendations Assessment Development and Evaluation (GRADE)[2]	**Strong (Grade 1)**: The panel is confident that the desirable effects of adherence to a recommendation outweigh the undesirable effects<br>**Weak (Grade 2)**: The panel concludes that the desirable effects of adherence to a recommendation probably outweigh the undesirable effects, but is not confident<br>**Best clinical practice (Guidance):** In the absence of any clear scientific evidence and because of undetermined balance between desirable and undesirable effects, judgment was based on the professional experience and consensus of the international experts within the working group	**High (A):** Further research is very unlikely to change our confidence in the estimate of effect<br>**Moderate (B):** Further research is likely to have an important impact on our confidence in the estimate of effect and may change the estimate<br>**Low (C):** Further research is very likely to have an important impact on our confidence in the estimate of effect<br>and is likely to change the estimate<br>**Very low (D):** Any estimate of effect is very uncertain
**Keeling *et al.* (2011)** [4]	Grading of Recommendations Assessment, Development, and Evaluation (GRADE) [2]	**1- strong:** clinicians are very certain that benefits do, or do not, outweigh risks and burdens.<br>**2- weak:** clinicians believe that benefits and risks and burdens are finely balanced, or appreciable uncertainty exists about the magnitude of benefits and risks	**A- high:** further research is very unlikely to change our confidence in the estimate of effect<br>**B- moderate:** further research is likely to have an important impact on our confidence in the estimate of effect and may change the estimate<br>**C- low:** further research is very likely to have an important impact on our confidence in the estimate of effect and is likely to change the estimate<br>**D- very low:** any estimate of effect is very uncertain, the objective criteria for assigning the quality of evidence shown in the table below should be used

Last Name of First Author (Year)	Grading System	Strength of Recommendation	Level of Evidence
**Watson** ***et al.*** **(2015)** [5]	Grading of Recommendations Assessment Development and Evaluation (GRADE)[2]	**1-Strong:** clinicians are very certain that benefits do, or do not, outweigh risks and burdens.<br>**2- Weak:** clinicians believe that benefits and risks and burdens are finely balanced, or appreciable uncertainty exists about the magnitude of benefits and risks	**A- High:** further research is very unlikely to change our confidence in the estimate of effect<br>**B- Moderate:** further research is likely to have an important impact on our confidence in the estimate of effect and may change the estimate<br>**C- Low:** further research is very likely to have an important impact on our confidence in the estimate of effect and is likely to change the estimate<br>**D- Very Low:** any estimate of effect is very uncertain
**Whitlock** ***et al.*** **(2012)** [6]	American College of Chest Physicians (ACCP) modified approach to Grading of Recommendations Assessment, Development and Evaluation (GRADE) [7]	**1: Strong**- experts are very certain that benefits do or do not outweigh risks, burden, and costs<br>**2: Weak**- experts are less certain that of the magnitude of the benefits and risks, burden, and costs and their relative impacts	**A**: high-quality evidence- randomized controlled trials and high-quality observational studies with large, consistent, effects<br>**B**: moderate-quality evidence<br>**C**: low quality or very low-quality evidence
**Bates** ***et al.*** **(2012)** [8]	American College of Chest Physicians (ACCP) modified approach to Grading of Recommendations Assessment, Development and Evaluation (GRADE) [7]	**1: Strong**- experts are very certain that benefits do or do not outweigh risks, burden, and costs<br>**2: Weak**- experts are less certain that of the magnitude of the benefits and risks, burden, and costs and their relative impacts	**A**: high-quality evidence- randomized controlled trials and high-quality observational studies with large, consistent, effects<br>**B**: moderate-quality evidence<br>**C**: low quality or very low-quality evidence
**Douketis** ***et al.*** **(2012)** [9]	American College of Chest Physicians (ACCP) modified approach to Grading of Recommendations Assessment, Development and Evaluation (GRADE) [7]	**1- strong**: experts are very certain that benefits do or do not outweigh risks, burden, and costs<br>**2- weak:** experts are less certain that of the magnitude of the benefits and risks, burden, and costs and their relative impacts	**A**: high-quality evidence- randomized controlled trials and high-quality observational studies with large, consistent, effects<br>**B**: moderate-quality evidence<br>**C:** low quality or very low-quality evidence
**Falck-Ytter** ***et al.*** **(2012)** [10]	American College of Chest Physicians (ACCP) modified approach to Grading of Recommendations Assessment, Development and Evaluation (GRADE) [7]	**1: Strong**- experts are very certain that benefits do or do not outweigh risks, burden, and costs<br>**2: Weak**- experts are less certain that of the magnitude of the benefits and risks, burden, and costs and their relative impacts	**A**: high-quality evidence- randomized controlled trials and high-quality observational studies with large, consistent, effects<br>**B**: moderate-quality evidence<br>**C**: low quality or very low-quality evidence

Last Name of First Author (Year)	Grading System	Strength of Recommendation	Level of Evidence
**Gould *et al.* (2012)** [11]	American College of Chest Physicians (ACCP) modified approach to Grading of Recommendations Assessment, Development and Evaluation (GRADE) [7]	**1: Strong**- experts are very certain that benefits do or do not outweigh risks, burden, and costs<br>**2: Weak**- experts are less certain that of the magnitude of the benefits and risks, burden, and costs and their relative impacts	**A**: high-quality evidence- randomized controlled trials and high-quality observational studies with large, consistent, effects<br>**B**: moderate-quality evidence<br>**C**: low quality or very low-quality evidence
**Holbrook *et al.* (2012) [12]**	American College of Chest Physicians (ACCP) modified approach to Grading of Recommendations Assessment, Development and Evaluation (GRADE) [7]	**1: Strong**- experts are very certain that benefits do or do not outweigh risks, burden, and costs<br>**2: Weak**- experts are less certain that of the magnitude of the benefits and risks, burden, and costs and their relative impacts	**A**: high-quality evidence- randomized controlled trials and high-quality observational studies with large, consistent, effects<br>**B**: moderate-quality evidence<br>**C**: low quality or very low-quality evidence
**Kahn *et al.* (2012)** [13]	American College of Chest Physicians (ACCP) modified approach to Grading of Recommendations Assessment, Development and Evaluation (GRADE) [7]	**1: Strong**- experts are very certain that benefits do or do not outweigh risks, burden, and costs<br>**2: Weak**- experts are less certain that of the magnitude of the benefits and risks, burden, and costs and their relative impacts	**A**: high-quality evidence- randomized controlled trials and high-quality observational studies with large, consistent, effects<br>**B**: moderate-quality evidence<br>**C**: low quality or very low-quality evidence
**Kearon *et al.* (2012)** [14]	American College of Chest Physicians (ACCP) modified approach to Grading of Recommendations Assessment, Development and Evaluation (GRADE) [7]	**1: Strong**- experts are very certain that benefits do or do not outweigh risks, burden, and costs<br>**2: Weak**- experts are less certain that of the magnitude of the benefits and risks, burden, and costs and their relative impacts	**A**: high-quality evidence- randomized controlled trials and high-quality observational studies with large, consistent, effects<br>**B**: moderate-quality evidence<br>**C**: low quality or very low-quality evidence
**Linkins *et al.* (2012)** [15]	American College of Chest Physicians (ACCP) modified approach to Grading of Recommendations Assessment, Development and Evaluation (GRADE) [7]	**1: Strong**- experts are very certain that benefits do or do not outweigh risks, burden, and costs<br>**2: Weak**- experts are less certain that of the magnitude of the benefits and risks, burden, and costs and their relative impacts	**A**: high-quality evidence- randomized controlled trials and high-quality observational studies with large, consistent, effects<br>**B**: moderate-quality evidence<br>**C**: low quality or very low-quality evidence

Last Name of First Author (Year)	Grading System	Strength of Recommendation	Level of Evidence
**Chan** ***et al*****.** **(2014)** [16]	Adapted from the Evaluation of Evidence criteria described in the Canadian Task Force on Preventative Health Care [17]	**A-** good evidence to recommend the clinical preventive action<br>**B-** fair evidence to recommend the clinical preventive action<br>**C-** evidence is conflicting and does not allow to make a recommendation for or against use of the clinical preventive action; however, other factors may influence decision-making<br>**D-** fair evidence to recommend against the clinical preventive action<br>**E-** good evidence to recommend against the clinical preventive action<br>**L-** insufficient evidence (in quantity or quality) to make a recommendation; however, other factors may influence decision-making	**I-** at least one properly randomized controlled trial<br>**II-1-** well-designed controlled trials without randomization<br>**II-2-** well-designed cohort (prospective or retrospective) or case–control studies, preferably from more than one centre or research group<br>**II-3**- comparisons between times or places with or without the intervention or dramatic results in uncontrolled experiments<br>**III-** opinions of respected authorities, based on clinical experience, descriptive studies, or reports of expert committees
**Liu** ***et al.*** **(2015)** [18]	American Heart Association clinical practice methodology Jacobs et al (2013) [19] and the Grading of Recommendations Assessment, Development, and Evaluation (GRADE) system [20-22]	**Strong-** most patients should receive the recommended treatment<br>**Weak-** different choices will be appropriate for different patients; management decision should be made in concert with the patient's values and preferences<br><br>**I-** benefit >>> risk, procedure or treatment should be performed or administered<br>**IIa-** benefit >> risk- additional studies with focused objectives are needed; it is reasonable to perform the procedure or administer the treatment<br>**IIb-** benefit ≥ risk- additional studies with broad objectives are needed, and additional registry data would be helpful; procedure or treatment may be considered<br>**III-** no benefit or **III**- harm: procedure or treatment should not be performed or administered because it is not helpful and may be harmful	**A-** multiple randomized controlled trials or meta-analyses; multiple populations evaluated<br>**B-** a single randomized controlled trial or non-randomized studies; limited populations evaluated<br>**C-** consensus of experts only, case studies or standard of care; very limited populations evaluated

Last Name of First Author (Year)	Grading System	Strength of Recommendation	Level of Evidence
**Easaw *et al.* (2015)** [23]	University of Oxford Centre for Evidence-Based Medicine (CEBM) Levels of Evidence (March 2009) [24]	**A:** consistent level 1 studies<br>**B:** consistent level 2 or 3 studies or extrapolations from level 1 studies<br>**C:** level 4 studies or extrapolations from level 2 or 3 studies<br>**D:** level 5 or troublingly inconsistent or inconclusive studies of any level	**1a:** systematic review (with homogeneity) of randomized controlled trials<br>**1b**: individual randomized controlled trials (with narrow confidence interval)<br>**1c**: all or none<br><br>**2a:** Systematic review (with homogeneity) of cohort studies<br>**2b**: Individual cohort study (including low quality randomized controlled trials)<br>**2c:** "Outcomes" research; ecological studies<br>**3a:** Systematic reviews with homogeneity of case-control studies<br>**3b:** Individual case-control study<br>**4:** Case-series (and poor quality cohort and case-control studies)<br>**5:** Expert opinion without explicit critical appraisal or based on physiology, bench research or "first principles"
**Easaw *et al.* (2015)** [25]	University of Oxford Centre for Evidence-Based Medicine (CEBM) Levels of Evidence (March 2009) [24]	**A:** consistent level 1 studies<br>**B:** consistent level 2 or 3 studies or extrapolations from level 1 studies<br>**C:** level 4 studies or extrapolations from level 2 or 3 studies<br>**D:** inconsistent or inconclusive studies of any level	**1:** a systematic review of homogenous randomized controlled trials or a single randomized controlled trial with a narrow confidence interval<br>**2:** a systematic review of homogenous cohort studies, or an individual cohort study or a low-quality randomized controlled trials<br>**3:** a systematic review of case–control studies or an individual case–control study<br>**4:** case series and poor-quality cohort and case–control studies<br>**5:** expert opinion without explicit critical appraisal
**James *et al.* (2011, re-affirmed 2017)** [26]	Method outlined by the United States Preventative Services Task Force [27]	**A**: based on good and consistent scientific evidence<br>**B:** based on limited or inconsistent scientific evidence<br>**C:** based primarily on consensus and expert opinion	**I**- at least one properly designed randomized controlled trial<br>**II-**1: well-designed controlled trials without randomization<br>**II-2:** well-designed cohort or case-control analytic studies, preferably from more than one center or research group<br>**II-3:** multiple time-series with or without the intervention. Could include 'dramatic' results in uncontrolled experiments also could be regarded as this type of evidence<br>**III:** opinions of respected authorities, based on clinical experience, descriptive studies, or reports of expert committees

Last Name of First Author (Year)	Grading System	Strength of Recommendation	Level of Evidence
**Siragusa *et al.* (2012)** [28]	Modified from: Scottish Intercollegiate Guidelines Network (SIGN) 50 Grading System [29] and the National Institute for Clinical Excellence (NICE) system [30]	**A -** At least one systematic reviews of randomized controlled trials or a single randomized controlled trial of level 1++ directly relevant for the target population, or level 1+ studies directly relevant for the target population yet with consistent results<br>**B -** Level 2++ studies directly relevant for the target population, or indirect evidence from level 1++ o 1+ studies<br>**C -** Level 2+ studies directly relevant for the target population, or indirect evidence from level 2++ studies<br>**D -** Level 3 or 4 directly relevant for the target population, or indirect evidence from level 2+ studies	**1++** High quality meta-analyses, systematic reviews of RCTs, or RCTs with a very low risk of bias<br>**1+** Well-conducted meta-analyses, systematic reviews, or RCTs with a low risk of bias<br>**1-** Meta-analyses, systematic reviews, or RCTs with a high risk of bias<br>**2++** High quality systematic reviews of case control or cohort or studies<br>High quality case control or cohort studies with a very low risk of confounding or bias and a high probability that the relationship is causal<br>**2+** Well-conducted case control or cohort studies with a low risk of confounding or bias and a moderate probability that the relationship is causal<br>**2-** Case control or cohort studies with a high risk of confounding or bias and a significant risk that the relationship is not causal<br>**3** Non-analytic studies, e.g. case reports, case series<br>**4** Expert opinion
**Streiff *et al.* (2011)** [31]	NCCN Categories of Evidence and Consensus [32]	**1-** uniform NCCN consensus<br>**2A-** uniform NCCN consensus<br>**2B-** non- uniform NCCN consensus (but no major disagreement)<br>**3-** major disagreement	**1-** high-level evidence (e.g., randomized controlled trials)<br>**2A-** lower level evidence<br>**2B-** lower level evidence<br>**3-** any level of evidence

Last Name of First Author (Year)	Grading System	Strength of Recommendation	Level of Evidence
**Mandala *et al.* (2011)** [33]	The American Society of Clinical Oncology [34]	**A-** evidence of type I or consistent findings from multiple studies of types II, III, IV<br>**B-** evidence of types II, III, or IV and findings are generally consistent<br>**C-** evidence of types II, III, or IV but findings are inconsistent<br>**D-** little or no systematic empirical evidence	**I-** meta-analysis of multiple, well-designed, controlled studies<br>- randomised trials with low false positive and low false-negative errors<br>**II-** at least one well-designed experimental study<br>- randomised trials with high false-positive and/or negative errors (low power)<br>**III-** well-designed, quasi-experimental studies such as non-randomised, controlled single group, pre-post, cohort, time, or matched case–control studies<br>**IV-** evidence is from well-designed, non-experimental studies such as comparative and correlational descriptive and case studies<br>**V-** evidence from case reports and clinical examples
**Greenberg *et al.* (2014) [35]**	Not reported	**I-** generally should be performed<br>**II-** may be reasonable to perform<br>**III-** generally should not be performed	**A-** randomized controlled trials<br>**B-** controlled trials with no randomization<br>**C-** observational studies<br>**D-** opinion of expert panel
**Lyman *et al.* (2015) [36]**	Not reported	**Strong:** high confidence the recommendation reflects best practice, based on:<br>1: strong evidence for a true net effect<br>2: consistent results with no or minor exceptions<br>3: minor or no concerns about study quality<br>4: the extent of panelists agreement<br>5: other considerations may also warrant a strong recommendation<br>**Moderate:** moderate confidence the recommendation reflects best practice, based on:<br>1: good evidence for a true net effect<br>2: consistent results, with minor and/or few exceptions<br>3: minor and/or few concerns about study quality<br>4: the extent of panellists' agreement<br>5: other considerations may also warrant a moderate recommendation<br>**Weak:** some confidence that the recommendation offers the best current guidance for practice, based on:<br>1: limited evidence for a true net effect<br>2: consistent results, but with important exceptions<br>3: concerns about study quality	**High:** High confidence that available evidence reflects true magnitude and direct of net effect. Further research is very unlikely to change either the magnitude or direction of this net effect<br><br>**Intermediate:** Moderate confidence that the available evidence reflects the true magnitude and direction of the net effect. Further research is unlikely to alter the direction of the net effect but might alter the magnitude of the net effect<br><br>**Low:** Low confidence that the available evidence reflects the true magnitude and direction of the net effect. Further research may change either the magnitude and direction of the net effect

Last Name of First Author (Year)	Grading System	Strength of Recommendation	Level of Evidence
		4: the extent of panelists' agreement<br>5: other considerations may also warrant a weak recommendation	
**Carrier *et al.* (2015) [37]**	Not reported	**1.** Strongly agree ("strongly recommend")<br>**2.** Somewhat agree ("recommend")<br>**3.** Neutral ("recommend")<br>**4.** Somewhat disagree ("recommend")<br>**5.** Strongly disagree ("suggest")	**Ia**- systematic review of randomized controlled trials<br>**Ib-** individual randomized controlled trials with narrow confidence intervals<br>**IIa-** systematic reviews of cohort studies<br>**IIb**- individual cohort studies or low-quality randomized controlled trials<br>**IIIa**- systematic reviews of case–control studies<br>**IIIb-** individual case–control studies<br>**IV-** case series<br>**V-** expert opinion or formal consensus
**Nicolaides *et al.* (2013)** [38]	Three main reference articles were used as guidance [39-41]	**Not reported**	**High-** randomized controlled trials with consistent results, or systematic reviews that were directly applicable to the target population.<br>**Moderate-** randomized controlled trials with less consistent results, limited power or other methodological problems, which were directly applicable to the target population<br>- randomized controlled trials extrapolated to the target population from different group of patients<br>**Low-** well-conducted observational studies with consistent results that were directly applicable to the target population
**The Scottish Intercollegiate Guidelines Network (SIGN) (2014)** [16]	Grading of Recommendations Assessment Development and Evaluation (GRADE)[2, 29]	**A:** At least one meta-analysis, systematic review, or RCT rated as 1++, and directly applicable to the target population; or A body of evidence consisting principally of studies rated as 1+, directly applicable to the target population, and demonstrating overall consistency of results<br>**B:** A body of evidence including studies rated as 2++, directly applicable to the target population, and demonstrating overall consistency of results; or Extrapolated evidence from studies rated as 1++ or 1+<br>**C:** A body of evidence including studies rated as 2+, directly applicable to the target population and demonstrating overall consistency of results; or Extrapolated evidence from studies rated as 2++<br>**D:** Evidence level 3 or 4; or Extrapolated evidence from studies	**1++** High quality meta-analyses, systematic reviews of RCTs, or RCTs with a very low risk of bias<br>**1+** High quality meta-analyses, systematic reviews, or RCTs with a low risk of bias<br>**1-** Meta-analyses, systematic reviews, or RCTs with a high risk of bias<br>**2++** High quality systematic reviews of case control or cohort studies High quality case control or cohort studies with a very low risk of confounding or bias and a high probability that the relationship is causal<br>**2+** Well conducted case control or cohort studies with a low risk of confounding or bias and a moderate probability that the relationship is causal<br>**2-** Case control or cohort studies with a high risk of confounding or

Last Name of First Author (Year)	Grading System	Strength of Recommendation	Level of Evidence
		rated as 2+<br><br>**GOOD PRACTICE POINTS**<br>Recommended best practice based on the clinical experience of the guideline development group	bias and a significant risk that the relationship is not causal<br>**3** Non-analytic studies, eg case reports, case series<br>**4** Expert opinion
**Urbanek et al. (2016)** [42]	Grading of Recommendations Assessment Development and Evaluation (GRADE) [2]	1A — strong recommendation<br>1B — strong recommendation<br>1C — strong recommendation<br>2A — weak recommendation<br>2B — weak recommendation<br>2C — weak recommendation	1A- high-quality evidence according to EBM*<br>1B- moderate-quality evidence according to EBM<br>1C- low- or very low-quality scientific evidence<br>2A- high-quality evidence according to EBM (further studies probably will not have any significant influence on changes in suggested treatment method)<br>2B- moderate-quality evidence according to EBM (further studies may have significant influence on changes in suggested treatment method)<br>2C- low- or very low-quality scientific evidence (further studies probably will have significant influence on changes in suggested treatment method)

S1 Table References

1. Debourdeau P, Farge D, Beckers M, Baglin C, Bauersachs RM, Brenner B, et al. International clinical practice guidelines for the treatment and prophylaxis of thrombosis associated with central venous catheters in patients with cancer. Journal of thrombosis and haemostasis : JTH. 2013;11:71-80.
2. The GRADE Working Group. GRADE 2018 [cited 2018]. Available from: http://www.gradeworkinggroup.org/.
3. Farge D, Bounameaux H, Brenner B, Cajfinger F, Debourdeau P, Khorana AA, et al. International clinical practice guidelines including guidance for direct oral anticoagulants in the treatment and prophylaxis of venous thromboembolism in patients with cancer. The Lancet Oncology. 2016;17(10):e452-e66.
4. Keeling D, Baglin T, Tait C, Watson H, Perry D, Baglin C, et al. Guidelines on oral anticoagulation with warfarin - fourth edition. Br J Haematol. 2011;154(3):311-24.
5. Watson HGK. Guideline on aspects of cancer-related venous thrombosis. British Journal of Haematology. 2015;170:640-8.
6. Whitlock RP, Sun JC, Fremes SE, Rubens FD, Teoh KH, Physicians American College of C. Antithrombotic and thrombolytic therapy for valvular disease: Antithrombotic Therapy and Prevention of Thrombosis, 9th ed: American College of Chest Physicians Evidence-Based Clinical Practice Guidelines. Chest. 2012;141:e576S-e600S.
7. Guyatt G, Gutterman D, Baumann MH, Addrizzo-Harris D, Hylek EM, Phillips B, et al. Grading strength of recommendations and quality of evidence in clinical guidelines: report from an american college of chest physicians task force. Chest. 2006;129(1):174-81. Epub 2006/01/21. doi: 10.1378/chest.129.1.174. PubMed PMID: 16424429.
8. Bates SM, Greer IA, Middeldorp S, Veenstra DL, Prabulos AM, Vandvik PO, et al. VTE, thrombophilia, antithrombotic therapy, and pregnancy: Antithrombotic Therapy and Prevention of Thrombosis, 9th ed: American College of Chest Physicians Evidence-Based Clinical Practice Guidelines. Chest. 2012;141:e691S-e736S.
9. Douketis J, Spyropoulos A, Spencer F, Mayr M, Jaffer A, Eckman M, et al. Perioperative management of antithrombotic therapy. Antithrombotic therapy and prevention of thrombosis, 9th ed: American College of Chest Physicians evidence-based clinical practice guidelines. Chest. 2012;141(2 Suppl):e326S-e50S.
10. Falck-Ytter Y, Francis CW, Johanson NA, Curley C, Dahl OE, Schulman S, et al. Prevention of VTE in orthopedic surgery patients: Antithrombotic Therapy and Prevention of Thrombosis, 9th ed: American College of Chest Physicians Evidence-Based Clinical Practice Guidelines. Chest. 2012;141:e278S-e325S.
11. Gould MK, Garcia DA, Wren SM, Karanicolas PJ, Arcelus JI, Heit JA, et al. Prevention of VTE in nonorthopedic surgical patients: Antithrombotic Therapy and Prevention of Thrombosis, 9th ed: American College of Chest Physicians Evidence-Based Clinical Practice Guidelines. Chest. 2012;141:e227S-e77S.
12. Holbrook A, Schulman S, Witt DM, Vandvik PO, Fish J, Kovacs MJ, et al. Evidence-based management of anticoagulant therapy: Antithrombotic Therapy and Prevention of Thrombosis, 9th ed: American College of Chest Physicians Evidence-Based Clinical Practice Guidelines. Chest. 2012;141:e152S-e84S.

13. Kahn SR, Lim W, Dunn AS, Cushman M, Dentali F, Akl EA, et al. Prevention of VTE in nonsurgical patients: Antithrombotic Therapy and Prevention of Thrombosis, 9th ed: American College of Chest Physicians Evidence-Based Clinical Practice Guidelines. Chest. 2012;141:e195S-e226S.
14. Kearon C, Akl EA, Ornelas J, Blaivas A, Jimenez D, Bounameaux H, et al. Antithrombotic therapy for VTE disease: CHEST guideline and expert panel report. Chest. 2016;149(2):315-52.
15. Linkins LA, Dans AL, Moores LK, Bona R, Davidson BL, Schulman S, et al. Treatment and prevention of heparin-induced thrombocytopenia: Antithrombotic Therapy and Prevention of Thrombosis, 9th ed: American College of Chest Physicians Evidence-Based Clinical Practice Guidelines. Chest. 2012;141:e495S-e530S.
16. Chan W, Rey E, Kent N, Group' ViPGW, Chan W, Kent N, et al. Venous thromboembolism and antithrombotic therapy in pregnancy. J Obstet Gynaecol Can. 2014;36(6):527-53.
17. New grades for recommendations from the Canadian Task Force on Preventive Health Care. Cmaj. 2003;169(3):207-8. Epub 2003/08/06. PubMed PMID: 12900479; PubMed Central PMCID: PMCPMC167122.
18. Liu D, Peterson E, Dooner J, Baerlocher M, Zypchen L, Gagnon J, et al. Diagnosis and management of iliofemoral deep vein thrombosis: clinical practice guideline CMAJ. 2015;187(17):1288-96.
19. Jacobs A, Kushner F, Ettinger S, Anderson J, Ohman M, Albert N, et al. ACCF/AHA clinical practice guideline methodology summit report: a report of the American College of Cardiology Foundation/American Heart Association Task Force on Practice Guidelines. Circulation. 2013;January 15:268-310.
20. Brozek JL, Akl EA, Alonso-Coello P, Lang D, Jaeschke R, Williams JW, et al. Grading quality of evidence and strength of recommendations in clinical practice guidelines. Part 1 of 3. An overview of the GRADE approach and grading quality of evidence about interventions. Allergy. 2009;64(5):669-77. Epub 2009/02/13. doi: 10.1111/j.1398-9995.2009.01973.x. PubMed PMID: 19210357.
21. Brozek JL, Akl EA, Compalati E, Kreis J, Terracciano L, Fiocchi A, et al. Grading quality of evidence and strength of recommendations in clinical practice guidelines part 3 of 3. The GRADE approach to developing recommendations. Allergy. 2011;66(5):588-95. Epub 2011/01/19. doi: 10.1111/j.1398-9995.2010.02530.x. PubMed PMID: 21241318.
22. The British Society for Haematology. Proposing and writing a new BSH Guideline England, UK: The British Society for Haematology; 2017 [cited 2018]. Available from: https://b-s-h.org.uk/guidelines/proposing-and-writing-a-new-bsh-guideline/.
23. Easaw J, Shea-Budgell M, Wu C, Czaykowski P, Kassis J, Kuehl B, et al. Canadian consensus recommendations on the management of venous thromboembolism in patients with cancer. Part 2: treatment. Current Oncology. 2015;22:144-55.
24. Centre for Evidence-Based Medicine. Oxford Centre for Evidence-based Medicine- Levels of Evidence (March 2009) Oxford, UK: Centre for Evidence-Based Medicine; 2009 [cited 2018]. Available from: https://www.cebm.net/2009/06/oxford-centre-evidence-based-medicine-levels-evidence-march-2009/.
25. Easaw JC, Shea-Budgell MA, Wu CM, Czaykowski PM, Kassis J, Kuehl B, et al. Canadian consensus recommendations on the management of venous thromboembolism in patients with cancer. Part 1: prophylaxis. Current Oncology. 2015;22:133-43.
26. James A, Committee on Practice Bulletins- Obstetrics. Practice bulletin no. 123: thromboembolism in pregnancy. ObstetGynecol. 2011;118(3):718-29.

27. U.S. Preventative Services Task Force. Standards for Guideline Development Rockville, MD: U.S. Preventative Services Task Force Program Office; 2016 [cited 2018]. Available from: https://www.uspreventiveservicestaskforce.org/Page/Name/standards-for-guideline-development.
28. Siragusa S, Armani U, Carpenedo M, Falanga A, Fulfaro F, Imberti D, et al. Prevention of venous thromboembolism in patients with cancer: Guidelines of the Italian Society for Haemostasis and Thrombosis (SISET). Thrombosis Research. 2012;129:e171-e6.
29. Scottish Intercollegiate Guidelines Network. Applying the GRADE methodology to SIGN guidelines: core principles Edinburgh: Scottish Intercollegiate Guidelines Network, Healthcare Improvement Scotland, 2010.
30. National Institute for Health and Care Excellence. The guidelines manual London, UK: National Institute for Health and Care Excellence; 2012 [cited 2018]. Available from: https://www.nice.org.uk/process/pmg6/chapter/reviewing-the-evidence.
31. Streiff M, Holstrom B, Ashrani A, Brockenstedt P, Chesney C, Eby C, et al. Cancer-Associated Venous Thromboembolic Disease, Version 1.2015. J Natl Compr Canc Netw. 2015;13(9):1079-95.
32. National Comprehensive Cancer Network. NCCN Guidelines & Clinical Resources Washington, PA National Comprehensive Cancer Network; 2018 [cited 2018]. Available from: https://www.nccn.org/professionals/physician_gls/categories_of_consensus.aspx.
33. Mandala M, Falanga A, Roila F, ESMO Guidelines Working Group. Management of venous thromboembolism (VTE) in cancer patients: ESMO Clinical Practice Guidelines. Ann Oncol. 2011;22(Suppl6):vi85-vi92.
34. American Society of Clinical Oncology. Guidelines, Tools, & Resources Alexandria, VA: American Society of Clinical Oncology; 2018 [cited 2018]. Available from: https://www.asco.org/practice-guidelines/quality-guidelines/guidelines.
35. Venous Thromboembolism Guideline Team. Venous thromboembolism (VTE): Guidelines for Clincial Care Ambulatory. Ann Arbor (MI): University of Michigan, 2014 May. Report No.
36. Lyman GH, Khorana AA, Kuderer NM, Lee AY, Arcelus JI, Balaban EP, et al. Venous thromboembolism prophylaxis and treatment in patients with cancer: American Society of Clinical Oncology clinical practice guideline update. J Clin Oncol. 2013;31:2189-204.
37. Carrier M, Lazo-Langer A, Shivakumar S, Tagalakis V, Gross P, Blais N, et al. Clinical challenges in patients with cancer-associated thrombosis: Canadian expert consensus recommendations. Curr Oncol. 2015;22(1):49-59.
38. Cardiovascular Disease Educational and Research Trust, European Venous Forum, North American Thrombosis Forum, International Union of Angiology, Union Internationale du Phlebologie. Prevention and treatment of venous thromboembolism: international consensus statement (guidelines according to scientific evidence). Clin Appl Thromb Hemost. 2013;19(2):116-225.
39. McAlister FA, Straus SE, Guyatt GH, Haynes RB. Users' guides to the medical literature: XX. Integrating research evidence with the care of the individual patient. Evidence-Based Medicine Working Group. Jama. 2000;283(21):2829-36. Epub 2000/06/06. PubMed PMID: 10838653.
40. McAlister FA, Clark HD, van Walraven C, Straus SE, Lawson FM, Moher D, et al. The medical review article revisited: has the science improved? Annals of internal medicine. 1999;131(12):947-51. Epub 1999/12/28. PubMed PMID: 10610646.
41. Moher D, Cook DJ, Eastwood S, Olkin I, Rennie D, Stroup DF. Improving the quality of reports of meta-analyses of randomised controlled trials: the QUOROM statement. Quality of Reporting of Meta-analyses. Lancet (London, England). 1999;354(9193):1896-900. Epub 1999/12/10. PubMed PMID: 10584742.

42. Urbanek T, Krasinski Z, Kostrubiec M, Sydor W, Wysocki P, Antoniewicz A, et al. Venous thromboembolism prophylaxis in cancer patients - guidelines focus on surgical patients. Acta Angiologica. 2016;22(3):71-102.
43. Antithrombotics: indications and management. Edinburgh, Scotland: Scottish Intercollegiate Guidelines Network (SIGN). 2013.
44. Trust A. Seven-point AGREE II Score Calculator. agreetrust.org; 2017.
