# Supplementary material for: A systematic review of clinical practice guidelines on the use of low molecular weight heparin and fondaparinux for the treatment and prevention of venous thromboembolism: Implications for research and policy decision-making
Source: PLoS One. 2018 Nov 9;13(11):e0207410. doi: 10.1371/journal.pone.0207410 (PMC6226206; doi:10.1371/journal.pone.0207410)
Supplement: S2 Table — (PDF) [file pone.0207410.s006.pdf]

S2 Table. General characteristics of included clinical practice guidelines.

Title (Publication Year)	Affiliated Organization(s)	Location of Origin	Evidence appraisal system	Indications addressed
VTE, thrombophilia, antithrombotic therapy, and pregnancy: Antithrombotic Therapy and Prevention of Thrombosis, 9th ed: American College of Chest Physicians Evidence-Based Clinical Practice Guidelines [8]	ACCP	United States	ACCP modified approach to GRADE	T5
Clinical challenges in patients with cancer-associated thrombosis: Canadian expert consensus recommendations [37]	Not reported	Canada	Not reported	T2, T4, P4
Venous thromboembolism and antithrombotic therapy in pregnancy [16]	SOGC	Canada	Adapted from the classification of recommendations criteria described in the CTFPHC	T5
International clinical practice guidelines for the treatment and prophylaxis of thrombosis associated with central venous catheters in patients with cancer [1]	GFTC; AMC; UMCG; INCa	France, The Netherlands	GRADE	T2, P4
Perioperative management of antithrombotic therapy. Antithrombotic therapy and prevention of thrombosis, 9th ed: American College of Chest Physicians evidence-based clinical practice guidelines [9]	ACCP	United States	ACCP modified approach to GRADE	P5
Canadian consensus recommendations on the management of venous thromboembolism in patients with cancer. Part 1: prophylaxis [25]	Not reported	Canada	CEBM Levels of Evidence (March 2009)	P3, P4
Canadian consensus recommendations on the management of venous thromboembolism in patients with cancer. Part 2: treatment [23]	Not reported	Canada	CEBM Levels of Evidence (March 2009)	T2, P4
Prevention of VTE in orthopedic surgery patients: Antithrombotic Therapy and Prevention of Thrombosis, 9th ed: American College of Chest Physicians Evidence-Based Clinical Practice Guidelines [10]	ACCP	United States	ACCP modified approach to GRADE	P2
International clinical practice guidelines for the treatment and prophylaxis of venous thromboembolism in patients with cancer [3]	GFTC; AMC; UMCG; INCa	France, The Netherlands	GRADE	T2, T5,P3,P4
Prevention of VTE in nonorthopedic surgical patients: Antithrombotic Therapy and Prevention of Thrombosis, 9th ed: American College of Chest Physicians Evidence-Based Clinical Practice Guidelines [11]	ACCP	United States	ACCP modified approach to GRADE	P3
Venous thromboembolism (VTE) [35]	UMHS	United States	Not reported	T1,T2,T3,T5
Evidence-based management of anticoagulant therapy: Antithrombotic Therapy and Prevention of Thrombosis, 9th ed: American College of Chest Physicians Evidence-Based Clinical Practice Guidelines [12]	ACCP	United States	ACCP modified approach to GRADE	T1
Practice bulletin no. 123: thromboembolism in pregnancy [26]	ACOG	United States	ACCP modified approach to GRADE	T5
Prevention of VTE in nonsurgical patients: Antithrombotic Therapy and Prevention of Thrombosis, 9th ed: American College of Chest Physicians Evidence-Based Clinical Practice Guidelines [13]	ACCP	United States	ACCP modified approach to GRADE	P4
Antithrombotic therapy for VTE disease: Antithrombotic Therapy and Prevention of Thrombosis, 9th ed: American College of Chest Physicians Evidence-Based Clinical Practice Guidelines [14]	ACCP	United States	ACCP modified approach to GRADE	T1,T2
Guidelines on oral anticoagulation with warfarin - fourth edition [4]	BSH	United Kingdom	GRADE	T1,T2,P5
Treatment and prevention of heparin-induced thrombocytopenia: Antithrombotic Therapy and Prevention of Thrombosis, 9th ed: American College of Chest Physicians Evidence-Based Clinical Practice Guidelines [15]	ACCP	United States	ACCP modified approach to GRADE	T1,P3

Title (Publication Year)	Affiliated Organization(s)	Location of Origin	Evidence appraisal system	Indications addressed
Diagnosis and management of iliofemoral deep vein thrombosis: clinical practice guideline Interdisciplinary Expert Panel on Iliofemoral Deep Vein Thrombosis [18]	CIRA	Canada	American Heart Association clinical practice methodology and GRADE system	T1,T2
Venous thromboembolism prophylaxis and treatment in patients with cancer: american society of clinical oncology clinical practice guideline update 2014 [36]	ASCO	United States	Not reported	T2,P3,P4
Management of venous thromboembolism (VTE) in cancer patients: ESMO clinical practice guidelines [33]	ESMO	Europe	Adapted from various approaches used by: AHRQ, the Cochrane Collaboration, SIGN; USPSTF; GRADE	T2,P3,P4
Prevention and treatment of venous thromboembolism: International consensus statement (Guidelines according to scientific evidence) [38]	CDER; EVF; NATF; IUA; and UIP	Czech Republic, France, North America, and the	Not reported	T1,T2,T5,P2 ,P3,P5
Antithrombotics: indications and management [43]	HEI, Scottish Health Council, SHTG, and SIGN, and SMC	United Kingdom	GRADE	T5,P3
Prevention of venous thromboembolism in patients with cancer: Guidelines of the Italian Society for Haemostasis and Thrombosis (SISET)1 [28]	SISET	Italy	Modified from: SIGN 50 Grading System and NICE system	P3,P4
Venous thromboembolic disease: Clinical practice guidelines in oncology [31]	NCCN	United States	NCCN Categories of Evidence and Consensus	T1,T2,P3,P4
Venous thromboembolism prophylaxis in cancer patients - guidelines focus on surgical patients [42]	Polish Society of Phlebotomy and the PTCN	Poland	GRADE	P3
Guideline on aspects of cancer-related venous thrombosis [5]	BCSH	United Kingdom	GRADE	T2,P3,P4
Antithrombotic and thrombolytic therapy for valvular disease: Antithrombotic Therapy and Prevention of Thrombosis, 9th ed: American College of Chest Physicians Evidence-Based Clinical Practice Guidelines [6]	ACCP	United States	ACCP modified approach to GRADE	T1,P5

Indication codes**: **T1: Treatment of DVT in patients without cancer; ***T2***: Treatment of symptomatic, acute, VTE in patients with cancer; ***T3***: Treatment of DVT in patients in whom treatment with warfarin is either not tolerated or contraindicated; ***T4***: Treatment of DVT in patients who fail treatment with warfarin; ***T5***: Treatment of DVT in pregnant and/or lactating women; ***P1***: Post-operative prophylaxis of DVT in patients undergoing hip or knee surgery and cannot use warfarin; ***P2***: Post-operative prophylaxis of VTE in patients undergoing surgery of the lower limbs; ***P3***: Prevention of VTE in patients undergoing non-orthopedic surgery; ***P4***: Prevention of VTE in patients with cancer; ***P5***: Peri-operative bridging for patients who require long-term warfarin and must discontinue due to surgery.

Abbreviations: American College of Chest Physicians ***(ACCP);*** American College of Obstetricians and Gynecologists ***(ACOG);*** Agency for Healthcare Research and Quality ***(AHRQ);*** Academic Medical Center ***(AMC);*** The American Society of Clinical Oncology ***(ASCO);*** British Committee for Standards in Haematology ***(BCSH);*** British Society for Haematology ***(BSH);*** Cardiovascular Disease Educational and Research Trust ***(CDER);*** University of Oxford Centre for Evidence-Based Medicine ***(CEBM);*** Canadian Interventional Radiology Association ***(CIRA);*** Canadian Task Force on Preventive Health Care ***(CTFPHC);*** European Society for Medical Oncology ***(ESMO);*** European Venous Forum ***(EVF);*** Groupe Francophone Thrombose et Cancer ***(GFTC);*** Recommendations Assessment Development and Evaluation***(GRADE);*** The Healthcare Environmental Inspectorate ***(HEI);*** French Institute of Cancer ***(INCa);*** International Union of Angiology ***(IUA);*** North American Thrombosis Forum ***(NATF);*** National Comprehensive Cancer Network ***(NCCN);*** National Institute for Clinical Excellence ***(NICE);*** Polish Society for Vascular Surgery (**PTCN**); Scottish Health Technologies Group ***(SHTG);*** Scottish Intercollegiate Guidelines Network ***(SIGN);*** Italian Society for Haemostasis and Thrombosis ***(SISET);*** Scottish Medicine Consortium ***(SMC);*** The Society of Obstetricians and Gynaecologists ***(SOGC);*** The Union Internationale du Phlebologie ***(UIP);*** University Medical Center Groningen ***(UMCG);*** University of Michigan Health System ***(UMHS);*** United States Preventive Services Task Force ***(USPSTF)***

S3 Table References

1. Bates SM, Greer IA, Middeldorp S, Veenstra DL, Prabulos AM, Vandvik PO, et al. VTE, thrombophilia, antithrombotic therapy, and pregnancy: Antithrombotic Therapy and Prevention of Thrombosis, 9th ed: American College of Chest Physicians Evidence-Based Clinical Practice Guidelines. Chest. 2012;141:e691S-e736S.

2. Carrier M, Lazo-Langer A, Shivakumar S, Tagalakis V, Gross P, Blais N, et al. Clinical challenges in patients with cancer-associated thrombosis: Canadian expert consensus recommendations. Curr Oncol. 2015;22(1):49-59.

3. Chan W, Rey E, Kent N, Group' ViPGW, Chan W, Kent N, et al. Venous thromboembolism and antithrombotic therapy in pregnancy. J Obstet Gynaecol Can. 2014;36(6):527-53.

4. Debourdeau P, Farge D, Beckers M, Baglin C, Bauersachs RM, Brenner B, et al. International clinical practice guidelines for the treatment and prophylaxis of thrombosis associated with central venous catheters in patients with cancer. Journal of thrombosis and haemostasis : JTH. 2013;11:71-80.

5. Douketis J, Spyropoulos A, Spencer F, Mayr M, Jaffer A, Eckman M, et al. Perioperative management of antithrombotic therapy. Antithrombotic therapy and prevention of thrombosis, 9th ed: American College of Chest Physicians evidence-based clinical practice guidelines. Chest. 2012;141(2 Suppl):e326S-e50S.

6. Easaw JC, Shea-Budgell MA, Wu CM, Czaykowski PM, Kassis J, Kuehl B, et al. Canadian consensus recommendations on the management of venous thromboembolism in patients with cancer. Part 1: prophylaxis. Current Oncology. 2015;22:133-43.

7. Easaw J, Shea-Budgell M, Wu C, Czaykowski P, Kassis J, Kuehl B, et al. Canadian consensus recommendations on the management of venous thromboembolism in patients with cancer. Part 2: treatment. Current Oncology. 2015;22:144-55.

8. Falck-Ytter Y, Francis CW, Johanson NA, Curley C, Dahl OE, Schulman S, et al. Prevention of VTE in orthopedic surgery patients: Antithrombotic Therapy and Prevention of Thrombosis, 9th ed: American College of Chest Physicians Evidence-Based Clinical Practice Guidelines. Chest. 2012;141:e278S-e325S.

9. Farge D, Bounameaux H, Brenner B, Cajfinger F, Debourdeau P, Khorana AA, et al. International clinical practice guidelines including guidance for direct oral anticoagulants in the treatment and prophylaxis of venous thromboembolism in patients with cancer. The Lancet Oncology. 2016;17(10):e452-e66.

10. Gould MK, Garcia DA, Wren SM, Karanicolas PJ, Arcelus JI, Heit JA, et al. Prevention of VTE in nonorthopedic surgical patients: Antithrombotic Therapy and Prevention of Thrombosis, 9th ed: American College of Chest Physicians Evidence-Based Clinical Practice Guidelines. Chest. 2012;141:e227S-e77S.

11. Venous Thromboembolism Guideline Team. Venous thromboembolism (VTE): Guidelines for Clincial Care Ambulatory. Ann Arbor (MI): University of Michigan, 2014 May. Report No.

12. Holbrook A, Schulman S, Witt DM, Vandvik PO, Fish J, Kovacs MJ, et al. Evidence-based management of anticoagulant therapy: Antithrombotic Therapy and Prevention of Thrombosis, 9th ed: American College of Chest Physicians Evidence-Based Clinical Practice Guidelines. Chest. 2012;141:e152S-e84S.

13. James A, Committee on Practice Bulletins- Obstetrics. Practice bulletin no. 123: thromboembolism in pregnancy. ObstetGynecol. 2011;118(3):718-29.

14. Kahn SR, Lim W, Dunn AS, Cushman M, Dentali F, Akl EA, et al. Prevention of VTE in nonsurgical patients: Antithrombotic Therapy and Prevention of Thrombosis, 9th ed: American College of Chest Physicians Evidence-Based Clinical Practice Guidelines. Chest. 2012;141:e195S-e226S.

15. Kearon C, Akl EA, Ornelas J, Blaivas A, Jimenez D, Bounameaux H, et al. Antithrombotic therapy for VTE disease: CHEST guideline and expert panel report. Chest. 2016;149(2):315-52.

16. Keeling D, Baglin T, Tait C, Watson H, Perry D, Baglin C, et al. Guidelines on oral anticoagulation with warfarin - fourth edition. Br J Haematol. 2011;154(3):311-24.

17. Linkins LA, Dans AL, Moores LK, Bona R, Davidson BL, Schulman S, et al. Treatment and prevention of heparin-induced thrombocytopenia: Antithrombotic Therapy and Prevention of Thrombosis, 9th ed: American College of Chest Physicians Evidence-Based Clinical Practice Guidelines. Chest. 2012;141:e495S-e530S.

18. Liu D, Peterson E, Dooner J, Baerlocher M, Zypchen L, Gagnon J, et al. Diagnosis and management of iliofemoral deep vein thrombosis: clinical practice guideline CMAJ. 2015;187(17):1288-96.

19. Lyman GH, Khorana AA, Kuderer NM, Lee AY, Arcelus JI, Balaban EP, et al. Venous thromboembolism prophylaxis and treatment in patients with cancer: American Society of Clinical Oncology clinical practice guideline update. J Clin Oncol. 2013;31:2189-204.

20. Mandala M, Falanga A, Roila F, ESMO Guidelines Working Group. Management of venous thromboembolism (VTE) in cancer patients: ESMO Clinical Practice Guidelines. Ann Oncol. 2011;22(Suppl6):vi85-vi92.

21. Cardiovascular Disease Educational and Research Trust, European Venous Forum, North American Thrombosis Forum, International Union of Angiology, Union Internationale du Phlebologie. Prevention and treatment of venous thromboembolism: international consensus statement (guidelines according to scientific evidence). Clin Appl Thromb Hemost. 2013;19(2):116-225.

22. Antithrombotics: indications and management. Edinburgh, Scotland: Scottish Intercollegiate Guidelines Network (SIGN). 2013.

23. Siragusa S, Armani U, Carpenedo M, Falanga A, Fulfaro F, Imberti D, et al. Prevention of venous thromboembolism in patients with cancer: Guidelines of the Italian Society for Haemostasis and Thrombosis (SISET). Thrombosis Research. 2012;129:e171-e6.

24. Streiff M, Holstrom B, Ashrani A, Brockenstedt P, Chesney C, Eby C, et al. Cancer-Associated Venous Thromboembolic Disease, Version 1.2015. J Natl Compr Canc Netw. 2015;13(9):1079-95.

25. Urbanek T, Krasinski Z, Kostrubiec M, Sydor W, Wysocki P, Antoniewicz A, et al. Venous thromboembolism prophylaxis in cancer patients - guidelines focus on surgical patients. Acta Angiologica. 2016;22(3):71-102.

26. Watson HGK. Guideline on aspects of cancer-related venous thrombosis. British Journal of Haematology. 2015;170:640-8.

27. Whitlock RP, Sun JC, Fremes SE, Rubens FD, Teoh KH, Physicians American College of C. Antithrombotic and thrombolytic therapy for valvular disease: Antithrombotic Therapy and Prevention of Thrombosis, 9th ed: American College of Chest Physicians Evidence-Based Clinical Practice Guidelines. Chest. 2012;141:e576S-e600S.
