# Supplementary material for: A systematic review of clinical practice guidelines on the use of low molecular weight heparin and fondaparinux for the treatment and prevention of venous thromboembolism: Implications for research and policy decision-making
Source: PLoS One. 2018 Nov 9;13(11):e0207410. doi: 10.1371/journal.pone.0207410 (PMC6226206; doi:10.1371/journal.pone.0207410)
Supplement: S3 Table — (PDF) [file pone.0207410.s007.pdf]

S3 Table. Mean (SD) AGREE II scores by domain and overall guideline quality assessment results.

Guideline (Author, YEAR)	Domain Score, % (Average SD)*¥						Overall Guideline Quality
	Scope and Purpose	Stakeholder Involvement	Rigour of Development	Clarity of Presentation	Applicability	Editorial Independence	
Scottish Intercollegiate Guidelines Network (2013) [43]	89 (0.47)	81 (0.24)	94 (0.18)	94 (0.47)	96 (0.35)	100 (0.00)	High
Bates (2012)[8]	94 (0.47)	94 (0.47)	81 (0.71)	100 (0.00)	48 (0.53)	96 (0.35)	High
Whitlock (2012)[6]	72 (0.94)	83 (0.94)	79 (0.53)	94 (0.00)	48 (0.53)	100 (0.00)	High
Linkins (2012)[15]	86 (1.18)	89 (0.94)	81 (0.35)	97 (0.24)	48 (0.53)	100 (0.00)	High
Kearon (2016)[14]	92 (0.71)	75 (0.24)	86 (0.44)	97 (0.24)	15 (0.53)	71 (0.35)	High
Fakck-Ytter (2012)[10]	83 (0.00)	89 (0.94)	84 (0.44)	94 (0.00)	44 (0.88)	100 (0.00)	High
Gould (2012)[11]	92 (0.71)	94 (0.47)	85 (0.35)	94 (0.00)	50 (0.71)	100 (0.00)	High
Kahn (2012)[13]	92 (0.71)	94 (0.47)	84 (0.44)	97 (0.24)	48 (0.53)	100 (0.00)	High
Holbrook (2012)[12]	86 (0.71)	94 (0.47)	83 (0.53)	92 (0.24)	50 (0.35)	100 (0.00)	High
Douketis (2012)[9]	89 (0.47)	97 (0.24)	84 (0.44)	94 (0.00)	46 (0.71)	100 (0.00)	High
Lyman (2013)[36]	86 (0.71)	83 (0.47)	84 (0.62)	97 (0.24)	19 (0.53)	67 (0.71)	High
Farge (2016)[3]	69 (0.71)	67 (0.00)	72 (0.44)	78 (0.47)	10 (0.18)	33 (0.00)	High
Siragusa (2012)[28]	72 (0.47)	33 (0.47)	69 (0.71)	69 (0.71)	4 (0.35)	25 (1.41)	High
Debourdeau (2013)[1]	64 (0.24)	50 (0.00)	69 (0.71)	69 (0.71)	2 (0.18)	33 (0.00)	High
Streiff (2015)[31]	69 (0.71)	69 (0.71)	53 (0.80)	89 (0.47)	8 (0.00)	96 (0.35)	Moderate
Liu (2015) [18]	72 (0.47)	72 (0.47)	40 (0.88)	97 (0.24)	8 (0.71)	71 (0.35)	Moderate

Guideline (Author, YEAR)	Domain Score, % (Average SD)*¥						Overall Guideline Quality
	Scope and Purpose	Stakeholder Involvement	Rigour of Development	Clarity of Presentation	Applicability	Editorial Independence	
Easaw (2015) [23]	72 (0.47)	56 (0.00)	51 (0.48)	94 (0.47)	4 (0.47)	46 (0.35)	Moderate
Cardiovascular Disease Educational and Research Trust (2013) [38]	64 (0.24)	28 (0.47)	51 (0.62)	81 (0.24)	19 (0.53)	50 (0.00)	Moderate
Carrier (2015) [37]	58 (1.18)	50 (0.94)	53 (0.80)	86 (0.24)	2 (0.18)	79 (0.35)	Moderate
Easaw (2015) [25]	83 (0.00)	56 (0.00)	42 (0.53)	97 (0.24)	4 (0.35)	46 (0.35)	Low
James (2017)[26]	69 (0.24)	25 (0.71)	23 (0.71)	69 (0.71)	4 (0.35)	0 (0.00)	Low
Watson (2015)[5]	67 (0.00)	58 (0.71)	42 (0.71)	78 (0.94)	4 (0.94)	0 (0.00)	Low
Mandala (2011)[33]	75 (0.71)	28 (0.00)	36 (0.62)	89 (0.47)	2 (0.18)	21 (0.35)	Low
Keeling (2011)[4]	69 (0.71)	53 (0.71)	36 (0.80)	94 (0.47)	2 (0.18)	0 (0.00)	Low
Chan (2014)[16]	83 (0.00)	50 (0.47)	33 (0.53)	92 (0.71)	4 (0.71)	4 (0.35)	Low
Venous Thromboembolism Guideline Team (University of Michigan) (2014) [35]	61 (0.94)	28 (0.47)	34 (0.44)	86 (0.71)	10 (0.53)	8 (0.00)	Low
Urbanek (2016) [42]	67 (0.94)	31 (0.24)	25 (0.71)	89 (0.47)	0 (0.00)	4 (0.35)	Low

* Calculations were made using the Seven-point AGREE II Score Calculator[44]
¥ Domain scores of ≥ 60% are coloured in green (domains were adequately addressed) and scores of < 60% (domains not adequately addressed) are coloured in red
Overall Guideline Quality: High = at least three of six AGREE II domains (including Domain 3) scored earned scores of >60%; **Moderate** = three or more AGREE II domains earned scores of >60%, except for Domain 3 OR at least two AGREE II domains earned scores of >60%, except for Domain 3, which earned a score of at least 50%; **Low** = any guideline not meeting the criteria for high or moderate quality.

S4 Table References

1. Antithrombotics: indications and management. Edinburgh, Scotland: Scottish Intercollegiate Guidelines Network (SIGN). 2013.

2. Bates SM, Greer IA, Middeldorp S, Veenstra DL, Prabulos AM, Vandvik PO, et al. VTE, thrombophilia, antithrombotic therapy, and pregnancy: Antithrombotic Therapy and Prevention of Thrombosis, 9th ed: American College of Chest Physicians Evidence-Based Clinical Practice Guidelines. Chest. 2012;141:e691S-e736S.

3. Whitlock RP, Sun JC, Fremes SE, Rubens FD, Teoh KH, Physicians American College of C. Antithrombotic and thrombolytic therapy for valvular disease: Antithrombotic Therapy and Prevention of Thrombosis, 9th ed: American College of Chest Physicians Evidence-Based Clinical Practice Guidelines. Chest. 2012;141:e576S-e600S.

4. Linkins LA, Dans AL, Moores LK, Bona R, Davidson BL, Schulman S, et al. Treatment and prevention of heparin-induced thrombocytopenia: Antithrombotic Therapy and Prevention of Thrombosis, 9th ed: American College of Chest Physicians Evidence-Based Clinical Practice Guidelines. Chest. 2012;141:e495S-e530S.

5. Kearon C, Akl EA, Ornelas J, Blaivas A, Jimenez D, Bounameaux H, et al. Antithrombotic therapy for VTE disease: CHEST guideline and expert panel report. Chest. 2016;149(2):315-52.

6. Falck-Ytter Y, Francis CW, Johanson NA, Curley C, Dahl OE, Schulman S, et al. Prevention of VTE in orthopedic surgery patients: Antithrombotic Therapy and Prevention of Thrombosis, 9th ed: American College of Chest Physicians Evidence-Based Clinical Practice Guidelines. Chest. 2012;141:e278S-e325S.

7. Gould MK, Garcia DA, Wren SM, Karanicolas PJ, Arcelus JI, Heit JA, et al. Prevention of VTE in nonorthopedic surgical patients: Antithrombotic Therapy and Prevention of Thrombosis, 9th ed: American College of Chest Physicians Evidence-Based Clinical Practice Guidelines. Chest. 2012;141:e227S-e77S.

8. Kahn SR, Lim W, Dunn AS, Cushman M, Dentali F, Akl EA, et al. Prevention of VTE in nonsurgical patients: Antithrombotic Therapy and Prevention of Thrombosis, 9th ed: American College of Chest Physicians Evidence-Based Clinical Practice Guidelines. Chest. 2012;141:e195S-e226S.

9. Holbrook A, Schulman S, Witt DM, Vandvik PO, Fish J, Kovacs MJ, et al. Evidence-based management of anticoagulant therapy: Antithrombotic Therapy and Prevention of Thrombosis, 9th ed: American College of Chest Physicians Evidence-Based Clinical Practice Guidelines. Chest. 2012;141:e152S-e84S.

10. Douketis J, Spyropoulos A, Spencer F, Mayr M, Jaffer A, Eckman M, et al. Perioperative management of antithrombotic therapy. Antithrombotic therapy and prevention of thrombosis, 9th ed: American College of Chest Physicians evidence-based clinical practice guidelines. Chest. 2012;141(2 Suppl):e326S-e50S.

11. Lyman GH, Khorana AA, Kuderer NM, Lee AY, Arcelus JI, Balaban EP, et al. Venous thromboembolism prophylaxis and treatment in patients with cancer: American Society of Clinical Oncology clinical practice guideline update. J Clin Oncol. 2013;31:2189-204.

12. Farge D, Bounameaux H, Brenner B, Cajfinger F, Debourdeau P, Khorana AA, et al. International clinical practice guidelines including guidance for direct oral anticoagulants in the treatment and prophylaxis of venous thromboembolism in patients with cancer. The Lancet Oncology. 2016;17(10):e452-e66.

13. Siragusa S, Armani U, Carpenedo M, Falanga A, Fulfaro F, Imberti D, et al. Prevention of venous thromboembolism in patients with cancer: Guidelines of the Italian Society for Haemostasis and Thrombosis (SISET). Thrombosis Research. 2012;129:e171-e6.

14. Debourdeau P, Farge D, Beckers M, Baglin C, Bauersachs RM, Brenner B, et al. International clinical practice guidelines for the treatment and prophylaxis of thrombosis associated with central venous catheters in patients with cancer. Journal of thrombosis and haemostasis : JTH. 2013;11:71-80.

15. Streiff M, Holstrom B, Ashrani A, Brockenstedt P, Chesney C, Eby C, et al. Cancer-Associated Venous Thromboembolic Disease, Version 1.2015. J Natl Compr Canc Netw. 2015;13(9):1079-95.

16. Liu D, Peterson E, Dooner J, Baerlocher M, Zypchen L, Gagnon J, et al. Diagnosis and management of iliofemoral deep vein thrombosis: clinical practice guideline CMAJ. 2015;187(17):1288-96.

17. Easaw J, Shea-Budgell M, Wu C, Czaykowski P, Kassis J, Kuehl B, et al. Canadian consensus recommendations on the management of venous thromboembolism in patients with cancer. Part 2: treatment. Current Oncology. 2015;22:144-55.

18. Cardiovascular Disease Educational and Research Trust, European Venous Forum, North American Thrombosis Forum, International Union of Angiology, Union Internationale du Phlebologie. Prevention and treatment of venous thromboembolism: international consensus statement (guidelines according to scientific evidence). Clin Appl Thromb Hemost. 2013;19(2):116-225.

19. Carrier M, Lazo-Langer A, Shivakumar S, Tagalakis V, Gross P, Blais N, et al. Clinical challenges in patients with cancer-associated thrombosis: Canadian expert consensus recommendations. Curr Oncol. 2015;22(1):49-59.

20. Easaw JC, Shea-Budgell MA, Wu CM, Czaykowski PM, Kassis J, Kuehl B, et al. Canadian consensus recommendations on the management of venous thromboembolism in patients with cancer. Part 1: prophylaxis. Current Oncology. 2015;22:133-43.

21. James A, Committee on Practice Bulletins- Obstetrics. Practice bulletin no. 123: thromboembolism in pregnancy. ObstetGynecol. 2011;118(3):718-29.

22. Watson HGK. Guideline on aspects of cancer-related venous thrombosis. British Journal of Haematology. 2015;170:640-8.

23. Mandala M, Falanga A, Roila F, ESMO Guidelines Working Group. Management of venous thromboembolism (VTE) in cancer patients: ESMO Clinical Practice Guidelines. Ann Oncol. 2011;22(Suppl6):vi85-vi92.

24. Keeling D, Baglin T, Tait C, Watson H, Perry D, Baglin C, et al. Guidelines on oral anticoagulation with warfarin - fourth edition. Br J Haematol. 2011;154(3):311-24.

25. Chan W, Rey E, Kent N, Group' ViPGW, Chan W, Kent N, et al. Venous thromboembolism and antithrombotic therapy in pregnancy. J Obstet Gynaecol Can. 2014;36(6):527-53.

26. Venous Thromboembolism Guideline Team. Venous thromboembolism (VTE): Guidelines for Clincial Care Ambulatory. Ann Arbor (MI): University of Michigan, 2014 May. Report No.

27. Urbanek T, Krasinski Z, Kostrubiec M, Sydor W, Wysocki P, Antoniewicz A, et al. Venous thromboembolism prophylaxis in cancer patients - guidelines focus on surgical patients. Acta Angiologica. 2016;22(3):71-102.

28. Trust A. Seven-point AGREE II Score Calculator. agreetrust.org; 2017.
