# Supplementary material for: A systematic review of clinical practice guidelines on the use of low molecular weight heparin and fondaparinux for the treatment and prevention of venous thromboembolism: Implications for research and policy decision-making
Source: PLoS One. 2018 Nov 9;13(11):e0207410. doi: 10.1371/journal.pone.0207410 (PMC6226206; doi:10.1371/journal.pone.0207410)
Supplement: S4 Table — (PDF) [file pone.0207410.s008.pdf]

S4 Table. Number of recommendations, the quality of CPGs reporting them, and the frequency with which each drug class and individual LMWH agents were discussed, by indication.

Indication<br>(# RECs)	# RECs by evidence level	# times LMWH and FDP mentioned (%)	CPG quality<br># (%)
**Treatment of:**			
Symptomatic, acute, VTE in cancer patients (k=40)	Level A: 13 (33)<br>Level B: 6 (15)<br>Level C: 12 (30)<br>Level D: 9 (23)	LMWH: 36 (73)<br>dalteparin: 3 (6)<br>enoxaparin: 2 (4)<br>tinzaparin: 4 (8)<br>FDP: 4 (8)	High: 4 (33)<br>Moderate: 5 (42)<br>Low: 3 (25)
DVT in pregnant and/or lactating females (k= 19)	Level A: 3 (16)<br>Level B: 9 (47)<br>Level C: 4 (21)<br>Level D: 3 (16)	LMWH: 17 (89)<br>FDP: 2 (11)	High: 2 (29)<br>Moderate: 1 (14)<br>Low: 3 (43)
DVT in non-cancer patients (k=15)	Level A: 3 (20)<br>Level B: 5 (33)<br>Level C: 7 (47)<br>Level D: 0	LMWH: 13 (72)<br>FDP: 5 (28)	High: 4 (57)<br>Moderate: 2 (29)<br>Low: 1 (14)
DVT in patients who failed treatment with warfarin (k=6)	Level A: 1 (17)<br>Level B: 0<br>Level C: 5 (83)<br>Level D: 0	LMWH: 6 (67)<br>FDP: 3 (33)	High: 2 (50)<br>Moderate: 2 (50)<br>Low: 0
DVT in patients who cannot tolerate warfarin or it is contraindicated (k=1)	Level A: 0<br>Level B: 1 (100)<br>Level C: 0<br>Level D: 0	LMWH: 1 (100)<br>FDP: 0	High: 0<br>Moderate: 0<br>Low: 1 (100)
**Prevention of:**			
VTE for patients undergoing non-orthopedic surgery (k=48)	Level A: 20 (42)<br>Level B: 19 (40)<br>Level C: 9 (19)<br>Level D: 0	LMWH: 46 (77)<br>dalteparin: 1 (2)<br>enoxaparin: 1 (2)<br>bevimarin: 1 (2)<br>FDP: 11 (18)	High: 5 (63)<br>Moderate: 1 (13)<br>Low: 2 (25)
VTE in cancer patients (k=19)	Level A: 4 (21)<br>Level B: 9 (47)<br>Level C: 5 (26)<br>Level D: 1 (5)	LMWH: 17 (68)<br>dalteparin: 2 (8)<br>enoxaparin: 3 (12)<br>tinzaparin: 1 (4)<br>nadroparin: 1 (4)<br>FDP: 1 (4)	High: 3 (33)<br>Moderate: 3 (33)<br>Low: 3 (33)
Post-operative VTE for patients undergoing orthopedic surgery of the lower limbs (k=12)	Level A: 5 (42)<br>Level B: 7 (58)<br>Level C: 0<br>Level D: 0	LMWH: 11 (58)<br>FDP: 8 (42)	High: 1 (50)<br>Moderate: 1 (50)<br>Low: 0
**Peri-operative bridging for:**			
Patients who require long-term warfarin and must discontinue due to surgery (k=8)	Level A: 4 (50)<br>Level B: 4 (50)<br>C: 0<br>D: 0	LMWH: 8 (100)<br>FDP: 0	High: 2 (50)<br>Moderate: 1 (25)<br>Low: 1 (25)

Abbreviations: *RECs: recommendations; CPG= clinical practice guideline, LMWH= low molecular weight heparin, FDP= fondaparinux*
Evidence level A: high-quality evidence; B: moderate-quality evidence; C: low quality evidence; D: expert opinion/consensus
NB: We did not identify any recommendations for the prevention of post-operative DVT in patients undergoing hip or knee surgery and cannot use warfarin
